# Molecular basis of *Arabidopsis* ABCC2 in plant detoxification

**DOI:** 10.1038/s41421-026-00919-z

**Published:** 2026-08-04

**Authors:** Jiangqing Dong, Tai-Li Yang, Xin-He Yu, Ke-Xin Hu, Lin-Po Xu, Yuan-Guang Jiang, Hong-Yan Lin, Guang-Fu Yang

**Affiliations:** 1 State Key Laboratory of Green Pesticide, Central China Normal University, Wuhan, Hubei China; 2 Hubei Shizhen Laboratory, Hubei University of Chinese Medicine, Wuhan, Hubei China; 3 School of Basic Medical Sciences, Hubei University of Chinese Medicine, Wuhan, Hubei China

**Keywords:** Plant molecular biology, Cryoelectron microscopy

**Dear Editor**,

The detoxification of phytotoxic compounds is a prerequisite for plant survival. ATP-binding cassette family C (ABCC) transporters play a pivotal role in the export of toxic compounds into vacuoles, a critical step in detoxification^1^. *Arabidopsis thaliana* ABCC2 (*At*ABCC2) is responsible for the efflux of glutathione conjugates of pesticides, such as atrazine and metolachlor, into the vacuoles^2–5^. Additionally, *At*ABCC2 can export arsenic-phytochelatin conjugates into vacuoles, resulting in increased arsenic tolerance^6,7^. Despite its critical role in plant detoxification, the biochemical and structural mechanisms underlying the function of *At*ABCC2 remain incompletely understood.

To address this gap, we present cryo-electron microscopy (cryo-EM) structures of *At*ABCC2 in four states: apo, substrate bound, closed, and dimeric. Structural analysis revealed a unique architecture, distinguished by the atypical localization of its transmembrane domain 0 (TMD0) domain. Moreover, biochemical studies revealed that the TMD0 domain is critical for coordinating transport channel closure. The molecular basis of atrazine export by *At*ABCC2 was also determined. Additionally, the plant-specific dimerization of *At*ABCC2 was demonstrated to be mediated by TMD2 and nucleotide-binding domain 2 (NBD2) rather than by the TMD0 domain. Notably, we found that although dimeric *At*ABCC2 represented a physiological form, its dimerization resulted in reduced substrate export activity. These findings provide new insights into the detoxification mechanism of *At*ABCC2 and highlight the potential for using ABCC transporters to develop herbicide-resistant crops.

Full-length *At*ABCC2 was recombinantly overexpressed in *Spodoptera frugiperda* 9 (Sf9) insect cells, and then purified by size-exclusion chromatography (SEC) in digitonin, which displayed two peaks corresponding to the dimer and monomer (Supplementary Fig. S1a–c). Wild-type (WT) *At*ABCC2 displayed basal ATPase activity, whereas the hydrolysis-deficient variant (E1404Q) exhibited significantly reduced ATPase activity (Supplementary Fig. S1d). As expected, the atrazine-glutathione adduct (AZ-GS) and other flavone and flavonol glucosides stimulated ATPase activity (Supplementary Fig. S1e, f). The herbicide tolerance of *At*ABCC2-overexpressing (OE) *Arabidopsis* was explored (Supplementary Fig. S1g–k). As expected, *At*ABCC2-OE *Arabidopsis* plants displayed longer roots than Col-0 plants at 0.5–1.0 μM atrazine.

Plant *At*ABCCs display relatively low TMD homology with ABCC members of other species (Supplementary Fig. S2). To elucidate the functional mechanism of plant ABCC transporters, we solved the cryo-EM structures of *At*ABCC2 in the apo, AZ-GS-bound, and closed states at 2.9 Å, 3.4 Å, and 2.9 Å, respectively (Fig. 1a, b; Supplementary Figs. S3–S6 and Table S1). *At*ABCC2 was composed of the TMD0, TMD1, TMD2, NBD1, and NBD2 domains (Fig. 1b; Supplementary Figs. S3, S6a). The TMD0 domain was an extra helix bundle at the N-terminus and was not detected in other reported plant ABC transporters (Supplementary Fig. S7a). It was linked to TMD1 through a lasso linker^8–10^, which was disordered in *At*ABCC2 structures. The regulatory R domain observed in mammalian ABCC2 was disordered in *At*ABCC2 structures, a region exhibiting low sequence conservation (Supplementary Fig. S2). TMD1, NBD1, TMD2, and NBD2 were linked sequentially and formed two half modules of the full transporter core.

**Fig. 1:**
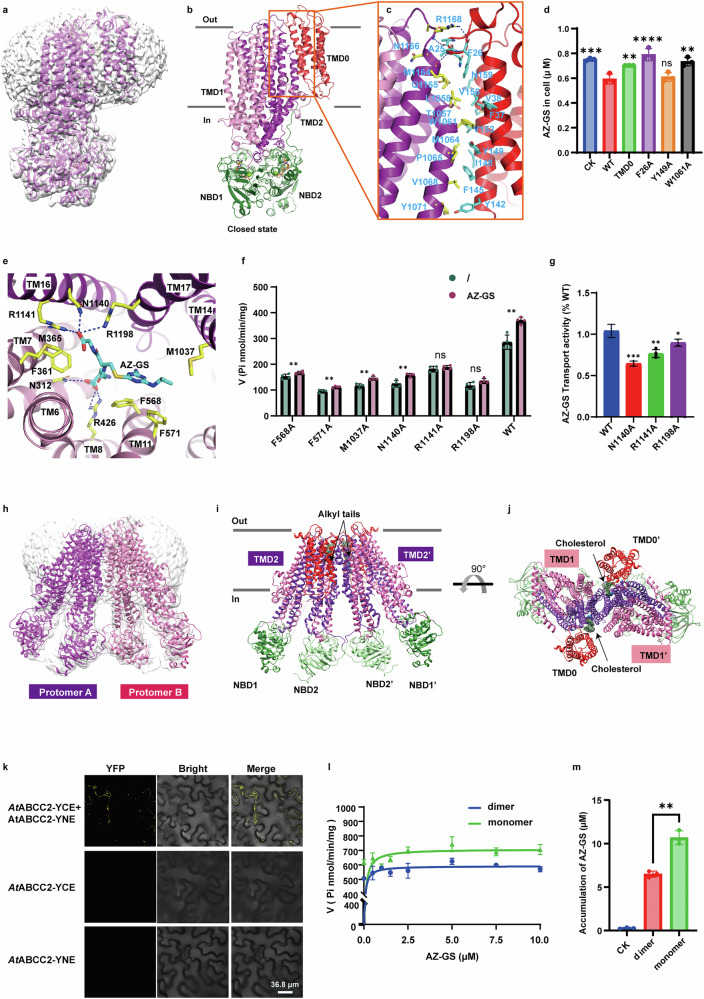
Structural and biochemical characterization of *At*ABCC2. **a** Cryo-EM map of *At*ABCC2 in closed state. **b** Cartoon representation of closed *At*ABCC2. **c** The interactions between TMD0 and TMD2 in closed state. Interacting residues are shown as sticks. Dashed lines indicate hydrogen bonds. **d** Cell-based AZ-GS transport activity of binding residue mutations. Data are shown as mean ± S.D. *n* = 3. Two-tailed Student’s *t*-test: *P < 0.05, **P < 0.01, ***P < 0.001, ****P < 0.0001. ns, not significant. **e** Binding of AZ-GS in *At*ABCC2. **f** ATPase activity of binding mutations stimulated by AZ-GS. **g** Liposome-based AZ-GS transport activity of mutations. *n* = 3. **h** Cryo-EM map of *At*ABCC2 in dimeric state. Two protomers are colored differently. **i**, **j** Cartoon representation of the dimeric *At*ABCC2. TMD0 and TMD0’, red; TMD1 and TMD1’, pink; TMD2 and TMD2’, purple; NBD1 and NBD1’, deep green; NBD2 and NBD2’, light green. The membrane is indicated with gray lines. Cholesterol molecules, green; alkyl tails, blue. **k** BiFC results to detect the fluorescence of intact YFP formed by dimeric *At*ABCC2. Scale bar, 36.8 μm. **l** ATPase activity of monomer and dimer stimu**l**ated by AZ-GS. **m** AZ-GS export activity of monomer and dimer using liposome-based transport assay. *n* = 3.

Unlike in human and yeast ABCCs^11,12^, in *At*ABCC2, TMD0 was located adjacent to TMD2, and these domains displayed a sandwich-like architecture together with TMD1 (Fig. 1b; Supplementary Figs. S3, S7b–g), which is consistent with published *At*ABCC2 structures^13^. Extensive interactions between TMD0 and the transporter core were observed in different states (Fig. 1c; Supplementary Fig. S8a, b). In particular, in the closed state (ATP-bound prehydrolysis state, without a substrate), the conformational coupling between TMD0 and the transporter core was tightened (Supplementary Fig. S8c, d). Hydrogen bonds formed between R1168 and T19, V22, or A23 were involved in the interactions. Residues, including F26, Y149, W1061, M1064, and M1164, mediated the hydrophobic interactions (Fig. 1c; Supplementary Fig. S8a, b). Intriguingly, many critical residues, including Thr19, Phe26, Tyr142, Trp1061, Met1164, and Arg1168, were conserved in only *At*ABCC1 and *At*ABCC2, not in *At*ABCC3, which was consistent with the distinct location of TMD0 in *At*ABCC2 (Supplementary Fig. S8e).

Mutations disrupting this interface abolished the stimulatory effect of AZ-GS on ATPase activity (Supplementary Fig. S8f, g). The AZ-GS transport activities of representative mutants were measured using a cell-based assay because of the low expression level of the protein when it was recombinantly expressed. Reduced transport of the F26A, W1061A, and TMD0 deletion mutants was observed (Fig. 1d; Supplementary Fig. S8h–j). To compare the WT and the TMD0‑deletion mutant, 200 ns molecular dynamics (MD) simulations were performed. The WT consistently exhibited a tendency for closing of the transport channel during the simulations (Supplementary Video S1), whereas the TMD0 deletion mutant showed unstable and less efficient channel closure (Supplementary Video S2). A close-up view of the transmembrane helices revealed that the transport channel of the WT maintained a more compact architecture than that of the deletion mutant (Supplementary Fig. S8k). These results indicate that TMD0 coordinates the closure of the transport channel and is essential for proper function.

Conformational changes induced by AZ-GS binding resulted in a narrower translocation cavity, similar to that induced by bovine MRP1 binding to leukotriene C_4_^14^ (Supplementary Fig. S9a, b). AZ-GS was deeply buried in the transmembrane pocket between TMD1 and TMD2. AZ-GS binding involves a positively charged area that coordinates the GSH moiety and a hydrophobic region that makes van der Waals interactions with the atrazine component (Fig. 1e; Supplementary Fig. S9c). The GSH moiety forms hydrogen bonds with residues N312 (TM6), R426 (TM8), N1140 and R1141 (TM16), and R1198 (TM17). Residues F361 (TM7), M365 (TM7), F568 (TM11), F571 (TM11), and M1037 (TM14) form hydrophobic interactions with AZ-GS. Surprisingly, sequence alignment revealed that critical residues mediating polar contacts with AZ-GS (N312, R426, N1140, R1141, and R1198) were not conserved in *At*ABCC3 (Supplementary Fig. S9d). Additionally, the binding site of DNP‑GS only partially overlaps with that of AZ‑GS (Fig. 1e; Supplementary Fig. S9e). On one side of the GSH moiety in DNP-GS, N1140, R1141, and R1198 are involved in coordination, similar to AZ-GS binding. In contrast, the opposite side lacks polar contacts with N312 and R426. Furthermore, additional residues within TM14 and TM17 contribute to hydrophobic interactions with DNP‑GS. *At*Atm3 (*At*ABCB25) also mediates the export of glutathione derivatives^15^. Its structure in complex with GSSG suggests the symmetric binding of GSSG, indicating a distinct transport mechanism (Supplementary Fig. S9f).

The critical roles of the binding residues were further validated using mutational analyses. Compared with the WT, all the mutants displayed impaired ATPase activity (Fig. 1f). Among them, R1141A and R1198A could not be significantly stimulated by AZ-GS. With respect to AZ-GS transport, the proteoliposome-based transport assays revealed that N1140A, R1141A, and R1198A all modestly affected the transport activity (Fig. 1g). Among them, N1140A led to the most marked impairment of transport ability. These results were consistent with the roles of these residues in substrate binding and export.

From the SEC chromatogram, *At*ABCC2 adopted both monomeric and dimeric states in solution (Supplementary Fig. S1a, b). Two-dimensional (2D) class averaging of the particles revealed both monomeric and dimeric *At*ABCC2 particles from one dataset (Supplementary Fig. S4). Finally, the dimeric classes yielded a map of dimeric *At*ABCC2 at 3.4 Å resolution using C2 symmetry (Fig. 1h–j; Supplementary Table S1). The TM helices of TMD0, TMD1, TMD2, and NBD2 from the two protomers were well resolved (Supplementary Fig. S10a). NBD1 and NBD1’ from the two protomers displayed relatively weak cryo-EM density. The overall conformation of dimeric *At*ABCC2 is consistent with a recently reported structure, with a root‑mean‑square deviation (RMSD) of 1.1 Å for Cα atoms (Supplementary Fig. S7e). Unlike the dimerization mode found in yeast Ycf1p and hABCC1, the dimerization of *At*ABCC2 was mediated mainly by the TM helices of TMD2 (Fig. 1h–j; Supplementary Fig. S7f, g). *At*ABCC2 TMD0 was located away from the dimerization interface. In addition, NBD2 from the two protomers participated in dimerization, which was not detected in dimeric Ycf1p or hABCC1 (Fig. 1h–j; Supplementary Fig. S7f, g). Four cholesterol molecules and two alkyl tails of copurified lipids or detergents were present near the dimerization interface, influencing the interaction (Fig. 1i, j; Supplementary Fig. S10a).

Dimerization of *At*ABCC2 was mediated mainly by hydrophobic interactions between TMD2 and TMD2’ as well as polar contacts between NBD2 and NBD2’ (Supplementary Fig. S10b–e). Residues in TM12 and TM13, such as W919, M923, F934, W941, Y959, Y979, and W980, participated in the formation of hydrophobic interactions with the symmetry-related residues (Supplementary Fig. S10c, d). Additionally, the side chain of N1344 from one NBD2 formed hydrogen bonds with the main chain of N1229 and N1231 in the symmetric NBD2’ (Supplementary Fig. S10e). Sequence alignment of multiple ABCCs revealed that the interfacial residues were highly conserved in *At*ABCC1 but not in *At*ABCC3, indicating a distinct oligomerization state of *At*ABCC3 (Supplementary Fig. S10f, g). Another phenomenon observed in dimeric *At*ABCC2 was that the NBDs were closer even than they were in the AZ-GS-bound state, indicating an intermediate state before NBD dimerization (Supplementary Fig. S10h, i). We further compared the substrate binding pockets of the AZ-GS-bound and dimeric states. The side chains of the residues mediating AZ-GS binding did not exhibit significant conformational changes (Supplementary Fig. S10j). These analyses suggest that substrate binding in the pocket is not significantly influenced by dimerization.

To further investigate whether the dimeric state exists in plant cells, we used the bimolecular fluorescence complementation (BiFC) method with split yellow fluorescent protein (YFP). The N-terminal and C-terminal parts of YFP (YNE and YCE, respectively) were fused to the C-terminus of *At*ABCC2 and coexpressed in tobacco leaves. The results confirmed that the complex formed by *At*ABCC2 linked to the YNE and YCE brought the two fluorescent protein fragments close enough to eventually form a luminescent YFP (Fig. 1k). The TMD0 deletion mutant of *At*ABCC2 displayed similar results, which is consistent with the finding that TMD0 does not participate in dimer formation (Supplementary Fig. S11a). The recombinantly expressed *At*ABCC5 was shown to be a monomer by gel filtration (Supplementary Fig. S11b). As a negative control, YNE- and YCE-linked *At*ABCC5 were not close enough; therefore, no fluorescence could be detected (Supplementary Fig. S11c).

Considering that dimeric structures of ABC transporters have been revealed in humans and yeast and that their dimeric states display unique functional features, it is necessary to investigate the function of dimeric *At*ABCC2. Therefore, we separated the mixed protein using SEC and obtained pure monomeric and dimeric proteins (Supplementary Fig. S1b). Both monomers and dimers were subsequently used to measure ATPase activity under substrate stimulation (Fig. 1l). The stimulatory effect of AZ-GS could be observed for both the monomer and the dimer. We further measured the AZ-GS transport activity. Proteoliposome transport assays revealed that the substrate transport activity of the monomer was qualitatively greater than that of the dimer under the assay conditions (Fig. 1m).

To better understand how dimerization affects protein behavior, we performed 200-ns MD simulations on the dimer. The results revealed that NBD1, which does not participate in dimer formation, exhibited greater flexibility and tended to close the transport passage (Supplementary Fig. S11d and Video S3). In contrast, NBD2, which is responsible for dimer formation, remained in a more constrained state that impeded movement during the transport cycle (Supplementary Fig. S11d and Video S3). These observations suggest that the restricted motion of NBD2 likely serves as the key factor limiting the transport activity of the dimer. These results collectively suggested that dimeric *At*ABCC2 is present in plant cells and represents a functional form in *Arabidopsis* that mediates pesticide efflux, such as monomers, but that its transport activity might be suppressed by dimerization.

In this study, we elucidated the molecular mechanism of detoxification by plant ABCC transporters on the basis of extensive biochemical studies and cryo-EM structures (Supplementary Fig. S11e). These results suggest that *At*ABCC2 has a unique arrangement of domains. TMD0 was found to play a critical role in coordinating transport channel closure. Additionally, *At*ABCC2 adopted a unique dimerization mode, which potentially restricted the motion of the NBD and thereby possibly contributed to reducing transport activity (Supplementary Fig. S11e). Given that we could not fully exclude the possibility of dimer–monomer interconversion in proteoliposomes under the assay conditions, the conclusion that the monomer exhibits higher transport activity than the dimer should be interpreted qualitatively, and within the constraints of this in vitro system. Further research will be needed to determine the transport function of each form in vivo and how dynamic oligomeric exchange occurring in the native membrane environment may be regulated. Our results also confirmed that overexpression of *At*ABCC2 in *Arabidopsis* increased the herbicide resistance of plants. The AZ-GS bound state reveals the binding details for AZ-GS in the pocket and therefore reveals the molecular basis for pesticide export.

As we finalized our work, we noted the publication of another study primarily focusing on the cryo-EM structures of *At*ABCC2^13^. Our work not only provides structural insights into the function of *At*ABCC2 but also used a series of cell-based and liposome-based transport assays complemented by MD simulations to characterize the functional roles of both TMD0 and the dimeric state. First, we demonstrated that the uniquely positioned TMD0 domain not only is a structural feature but also plays an active and essential role in driving transport-channel closure. This functional validation assigns a precise mechanistic purpose to the distinct localization of TMD0. Moreover, we confirm that the observed dimerization occurs *in planta*. Comparative activity measurements revealed that the dimer exhibited reduced transport activity under the assay conditions, indicating that the restricted motion of the NBDs within the dimer was linked to a dampened functional output. Structural comparison between AZ‑GS and DNP‑GS binding demonstrated that the substrate‑binding pocket has the capacity to accommodate structurally distinct ligands. Thus, our work provides a functional context for the existing structural framework and offers a mechanism-centered perspective on the regulation of plant ABCC transporters.

## Supplementary information

Supplementary Methods, Figures and Table

Related Manuscript File

Related Manuscript File

Related Manuscript File

Related Manuscript File

Supplementary Movie 1

Supplementary Movie 2

Supplementary Movie 3

## Data Availability

The cryo-EM maps and coordinates have been deposited in the EMDB and PDB under accession codes EMD-63864 and 9U56 (apo), EMD-63865 and 9U57 (dimeric), EMD-65915 and 9WET (closed), and EMD-63866 and 9U58 (AZ-GS bound).
