# Supplementary material for: Molecular basis of Arabidopsis ABCC2 in plant detoxification
Source: Cell Discov. 2026 Aug 4;12:57. doi: 10.1038/s41421-026-00919-z (PMC13439234; doi:10.1038/s41421-026-00919-z)
Supplement: Supplementary file 1 [file 41421_2026_919_MOESM1_ESM.pdf]

Materials and Methods

Protein expression and purification

The codon-optimized gene encoding full-length Arabidopsis ABCC2 (Uniprot: Q42093) was synthesized (General Biology, Anhui, China) and cloned into a modified pFastBac vector harboring a C-terminal eGFP tag and an 8×His tag or the pCAGGS vector with twin strep-flag tag. Site-directed mutagenesis was performed using PCR amplification with mutagenic primers followed by DpnI digestion and transformation. The primers were synthesized by General Biology, and the sequences are listed in Supplementary information. The plasmids were then transformed into DH10Bac cells to generate recombinant bacmid. Purified bacmids were transfected into Sf9 cells and the resulting baculovirus was amplified to P2 for protein expression. The P2 baculovirus was added at 2% v/v to Sf9 cells at a density of 2.0-3.0 million cells/mL. For the transient expression in HEK293F cells, 2 mg mutant plasmids were incubated with PEI at a 1:2 ratio for 15 minutes, then added to 800 ml cells and cultured for 8-12 hrs at 37°C. 10 mM sodium butyrate was added to the culture after 8-12 hrs and expression was allowed to proceed for approximately 48 hours at 30°C. Cells were harvested after 48-72 hrs expression at 27°C for sf9, flash frozen in liquid nitrogen, and stored at -80 °C.

For protein purification, cells were thawed and solubilized in buffer containing 150 mM NaCl, 20 mM HEPES pH 7.4, 10% glycerol, and the protease cocktail inhibitors (Sigma). The cells were lysed using sonication, and then proteins were extracted from cell membrane using 1% (w/v) n-Dodecyl-β-D-Maltopyranoside (DDM, ExceedBio) for two hours. After centrifugation at 45,000 rpm for 60 min, the supernatant was collected and mixed with GFP nanobody-conjugated Sepharose 4 Fast Flow resin (Cytiva) for 1 hour, while proteins expressed by transient transfection were mixed with Strep resin (IBA lifesciences). Resin was washed with buffer containing 150 mM NaCl, 20 mM HEPES pH 7.4, 10% glycerol, and 0.02% DDM. TEV protease was added to the resin at about 0.35 mg/mL and incubated for four hours at 4 °C to remove tag. The eluate containing target protein was collected and concentrated using a 100 kDa filter to approximately 0.5 mL and further purified by size exclusion chromatography at 4°C using a Superose 6 10/300 GL column (Cytiva) equilibrated with buffer containing 0.06% digitonin, 150 mM NaCl, 20 mM HEPES pH 7.4, and 2 mM DTT. Peak fractions containing *At*ABCC2 were pooled and concentrated for further biochemical studies or

cryo-EM analysis.

ATPase activity assay

ATPase activity was measured using a Malachite Green Phosphate Detection Kit (Beyotime). The purified 1.5 μg *At*ABCC2 and mutants was added to initiate the reaction with a mixture of 10 mM $MgCl_2$, 0.05% digitonin, 20 mM Hepes 7.4, 150 mM NaCl and ATP. The control assay did not contain protein, which could be used to eliminate phosphate released by itself. The reaction mixture contained 40 μL, and was incubated in 37°C for 40 min, then reacted with Malachite green and molybate at room temperature for 30 min. The release of phosphate was measured by monitoring the absorption at 630 nm using Synergy H1 microplate reader (Biotek, Winooski, VT). Rate of ATP hydrolysis was calculated from the standard curve, and then fitted by nonlinear regression to the Michaelis-Menten equation to calculate K_m and V_{max} values using GraphPad Prism 9.5. For the substrate-stimulated ATPase activity, 3 mM ATP was added in the reaction.

Identification of *At*ABCC2 by LC-MS/MS

Following SDS-PAGE separation, 5 mm gel strips were excised, rinsed with ddH_2O, and destained in 50% acetonitrile in 40 mM NH_4HCO_3. Gels were air dried to remove solvent, reduced with 25 mM dithiothreitol for 1 h at 55°C, and alkylated with 25 mM iodoacetamide in NH_4HCO_3 for 30 min at room temperature in the dark. After cleanup, samples were digested separately with trypsin (Promega) at 37°C for 14 h. Peptides were extracted using 65% acetonitrile and 5% formic acid in ddH_2O, then vacuum dried. Samples were desalted on Oasis HLB cartridges (Waters) following the manufacturer's protocol, eluted with 80% acetonitrile in ddH_2O, and dried to completeness.

The LC-MS/MS analysis was performed through modification of previously reported methods[1]. The peptides sample was loaded onto a reversed-phase μ-precolumn (particle size 3 μm; Dionex/Thermo Scientific) and peptide separation was performed using an analytical column (C18; particle size 2 μm; Thermo Scientific, Germany) at a flow rate of 0.3 μL/min. 0.1% Formic acid /H_2O (mobile phase A) and 80% Acetonitrile /0.1% Formic acid /20% H_2O (mobile phase B) were used for separation. Gradient elution mode was applied for a better separation. The gradient was set as follows: 0-3 min, 10%-12% B; 3-18 min, 12%-20% B; 18-48 min, 20%-35% B; 48-61 min, 35%-45% B; 61-70 min, 99%B. Eluting peptides were electrosprayed (2.25 kV; transfer capillary temperature 275°C) in positive ion mode into an Orbitrap Exploris480 tandem

mass spectrometer equipped with a Nanospray. Total acquisition time 70 min according to the gradient. FT full MS (m/z 350 to 1200; resolution 60000 FWHM) and ACG Target 300, RF Lens 50%, Mass width ± 10 ppm, Isolation Window 1.6 m/z, HCD Collision Energies 30, Exclusion duration 45 s. Peak lists were extracted from fragment ion spectra using the pFind.

Cryo-EM sample preparation and data collection

To prepare apo and closed *At*ABCC2, immediately following gel filtration, the protein was concentrated to about 10 mg/mL and 4 μL protein sample was applied to freshly glow-discharged QUANTIFOIL (R1.2/1.3, 300 mesh) Au grids. 5 mM $MgCl_2$ and 10 mM ATP (pH 7.4) were added and incubated at 37°C for 10 min to obtain closed state of *At*ABCC2. The grids were blotted with filter paper for 5.0 s, with a blotting force 3, and wait 0 s. Then, the grids were plunged into liquid ethane cooled by liquid nitrogen using a Vitrobot Mark IV (FEI) under 100% humidity at 8°C.

To prepare AZ-GS-bound *At*ABCC2, the protein was concentrated to 8.4 mg/ml and incubated with 0.78 mM AZ-GS at 4°C about 60 min before applied to freshly glow-discharged QUANTIFOIL (R1.2/1.3, 300 mesh) Au grids. The grids were blotted with filter paper for 6.0 s, with a blotting force 3, and wait 0 s. Then, the grids were plunged into liquid ethane cooled by liquid nitrogen using a Vitrobot Mark IV (FEI) under 100% humidity at 8°C.

Image processing

Drift correction for the movies was performed using MotionCor2[2]. Then, contrast function (CTF) estimation was performed using patch CTF estimation in cryoSPARC[3]. Particles were picked using template picker and extracted with a box size of 400 pixels after inspection. Particles were sorted by iterative 2D classification and the initial model was generated using ab-initio reconstruction. Well-sorted particles were finally subjected to homogenous and non-uniform refinements to generate the final maps. Resolution was estimated using the Fourier shell correlation (FSC) 0.143 criterion. Local resolution map was also calculated using cryoSPARC.

Model building and refinement

The AlphaFold predicted model of *At*ABCC2 was used as the initial model[4]. Model building was carried out in Coot[5]. The real-space refinement of the model was carried out using Phenix[6] with Ramachandran restraint and further manual adjustment in Coot. Molprobity was used to evaluate the stereochemistry and geometry of the

structures[7]. Figures were prepared using UCSF Chimera[8] and PyMOL (http://pymol.org).

MD simulations

Simulations were conducted with the Schrödinger (version 2023) Desmond module[9], starting from cryo-EM structures. Protein and ligand were prepared using the Protein Preparation and LigPrep modules, respectively. The transmembrane region was embedded in a 1-palmitoyl-2-oleoylphosphatidylcholine (POPC) lipid bilayer. The system was solvated in an orthorhombic water box, maintaining a minimum distance of 10 Å between the protein and box edges, with water modeled using TIP4P and Na^+ ions added for charge neutralization. All simulations were performed under the OPLS4 force field in the NPT ensemble at 300 K and 1.01325 bar[10,11]. An initial 100 ps minimization was applied for relaxation, followed by triplicate 200 ns production runs for each system. RMSD analysis was carried out using VMD (version 1.9.3)[12].

Proteoliposome preparation

25 mg dry Soy Extract Polar lipids (Sigma-Aldrich) was dissolved in buffer with 20 mM HEPES pH 7.4 and 150 mM NaCl using ultrasound. Then the lipids were extruded 21 times through a 400-nm polycarbonate filter (Cytiva) to form large unilamellar vesicles (LUVs). 10% (v/v) Triton X-100 was added in to destabilize the liposomes at room temperature with gentle agitation for 1 hour. Then wild-type protein or mutants were added to insert into the detergent-destabilized liposomes at 4℃ with gentle agitation for 1 hour. The detergent was removed with 100 mg/mL Bio-beads (Bio-Rad) overnight. Finally, the proteoliposomes were collected by centrifuging at 267,000 xg (TLA-110, Optima MAX-XP, BECKMAN COULTER) for 15 min, and suspended to 100 mg/mL with buffer (containing 20 mM HEPES pH 7.4, 150 mM NaCl, 10 mM $MgCl_2$).

Substrate transport assay

To test the uptake of AZ-GS (Santa Cruz Biotechnology) by *At*ABCC2 in proteoliposome, the substrate in a final concentration of 100 μM was added in the reaction buffer (20 mM HEPES pH 7.4, 150 mM NaCl, 10 mM $MgCl_2$, 5 mM ATP). The reaction was started with 2.5 μl proteolipsomes for 30 min and terminated by diluting in 2 mL ending buffer (20 mM HEPES pH 7.4, 150 mM NaCl), and then centrifuged immediately at 4℃, 267,000×g, 15 min. The pellet was washed with ending buffer twice and then dried in room temperature. Liposomes without protein was used as control. 50 μL methanol was added in to dissolve the proteoliposomes and

subsequently vigorously vortex the aliquots. Then the mixture was centrifuged again and the supernatants were quantitatively analyzed with LC-MS/MS.

Cell-based substrate transport assay

To analyze and compare the ability of different mutants to transport AZ-GS, 10 ml cells with the same cell number (approximately 2 million cells/ml) were transiently transfected with *At*ABCC2, the mutant plasmids, and then expressed for 48 hours. About 1 ml of the cells was added with 100 μM AZ-GS and incubated for 20 min with gentle rotation. Then, the cells were washed twice with PBS. Finally, 150 μl of chromatographic methanol was used to shake and incubate the cell precipitate for 30 min to extract intracellular AZ-GS. After centrifugation at 13,500 rpm for 10 min twice, the supernatant was transferred to mass spectrometry vials for LC-MS quantification of the AZ-GS content. Each mutant was tested in three replicates.

The remaining cells were used for western blot analysis to check the amount of the target protein. Approximately 2 ml cells were resuspended in 300 μl buffer, with protease inhibitors added, along with 1% DDM and lysed by rotating at 4°C for 30 minutes. The lysate was then centrifuged at 13,500 rpm for 10 minutes to remove cell debris. 20 μl sample mixed with 5 × loading buffer (Yamei, Shanghai) was separated by a 10% SDS-PAGE gel. Then, the protein samples were transferred to a PVDF membrane (0.45 μm, Cytiva) and blocked for 20 mins with blocking buffer (Yamei, Shanghai). The target protein was analyzed by immunoblotting using an anti-flag antibody (1:15000, Proteintech) and a HRP-conjugated goat anti-mouse IgG (H&L) as the secondary antibody (1:15000, ShareBio), while the internal reference protein, GAPDH, was analyzed by immunoblotting using an anti-GAPDH antibody (1:40000, Proteintech) and a HRP-conjugated goat anti-rabbit IgG (H&L) as the secondary antibody (1:20000, ShareBio). Subsequently, the membrane was rinsed with PBST three times and imaged with an e-Blot.

Identification and quantification of AZ-GS by LC-ESI-MS

AZ-GS was identified using ultra-high-performance liquid chromatography coupled with electrospray ionization and Q Exactive Orbitrap mass spectrometry (UHPLC–ESI–QE Orbitrap-MS, Thermo Fisher Scientific). The LC analysis was performed with a Hypersil GOLD^{TM} C18 analytical column（2.1 by 100 mm, with an inner diameter of 1.9 μm, Thermo Scientific）kept at 40 °C. The volume injected was 10 μL with a 200 μL/min flow rate and 17 min gradient. The gradient profile started at 20% solvent

B, increased to 70% B over 3 min, maintained at 70% B for 3 min, ramped to 90% B over 4 min, held at 90% B for 3 min, decreased to 20% B within 0.1 min, and then equilibrated at 20% B for 3.9 min. Mobile phase A consisted of 0.1% (v/v) formic acid in LC-MS grade water (Thermo Fisher Scientific). Mobile Phase B: Methanol (Thermo Fisher Scientific). AZ-GS was identified using full-scan mass spectrometry under positive electrospray ionization (ESI+) conditions. Instrument parameters were set as follows: resolution, 70,000; automatic gain control (AGC), 3×10^6; maximum injection time (IT), 200 ms.

Quantitative analysis of AZ-GS was performed using liquid chromatography parallel reaction monitoring (LC–PRM). Ionization conditions included a spray voltage of 3.8 kV and a capillary temperature of 320 °C. The acquisition comprised an MS^1 full scan from m/z 100 to 1000 at a resolution of 70,000, followed by PRM scans targeting m/z 487.20770 at a resolution of 17,500. AGC for MS2 was set as 2×10^5. The maximum injection time for MS^2 was 100 ms, the isolation window was set at 3 m/z, and normalized collision energy (NCE) was 35. Raw data acquired using the PRM method were processed using Xcalibur software (Thermo Fisher Scientific). The peak area of the product ion at m/z 214.1119, chosen for its high signal-to-noise ratio, was used for relative quantification. The absolute amount of AZ-GS in samples was determined from a standard calibration curve prepared using various concentrations.

Herbicide resistance assay

To analyze whether *Arabidopsis thaliana* overexpressing *At*ABCC2 is tolerant to atrazine, pEGOEP2X35S-H-*At*ABCC2-GFP was introduced into *Agrobacterium* GV3101 and transformed into *Arabidopsis thalian*a Col-0. Seeds from transgenic lines with stable inheritance whose expression levels were validated via reverse transcription quantitative PCR (RT-qPCR) were used for subsequent experiments. *Arabidopsis* seeds (Col-0 and 2×35S:*At*ABCC2-GFP) were surface sterilized with 75% ethanol for 5 min, 0.5% Sodium hypochlorite for 5 min, subsequently washed with sterile water three times, and spotted vertically on Murashige-Skoog solid medium containing the indicated concentrations of atrazine. After cold treatment for 2 days to break seed dormancy, the seeds were vertically grown under a 16-hour light regime. Seedling morphology were photographed 10 days after germination. The root length was measured using ImageJ.

RT-qPCR

For RT-qPCR, total RNA was extracted from 10-day old *Arabidopsis* seedlings using

the the Super FastPure Cell RNA Isolation Kit (Vazyme). 1 μg total RNA was used to eliminate genomic DNA by PrimeScript™ RT reagent Kit with gDNA Eraser (TaKaRa), and subsequently reverse transcribed into total cDNA. cDNA was analyzed on a LightCycler 480 II apparatus (Roche) with the Taq Pro Universal SYBR qPCR Master Mix (Vazyme) according to the manufacturer's instructions. Expression levels were normalized to those of ACTIN2 gene. Primers used for RT-qPCR were *At*ABCC2-F (TTCAAGTCATGCACGATGCTC), *At*ABCC2-R (AAGTGGTGTGAGTCATCTTTGG), ACTIN2-F (ACTCTCCCGCTATGTATGTCGC), ACTIN2-R (AGAAACCCTCGTAGATTGGCAC).

BiFC assay

The cDNA of full-length *At*ABCC2, *At*ABCC2 (residues 233-1623) and *At*ABCC5 was cloned from *Arabidopsis thaliana,* and fused in vector 21OV302_pC1300-mVYNE and 21OV303_pC2300-mVYCE. For each ABCC protein, the control groups were co-transformed with the complementary YNE or YCE empty plasmid. GV3101 (pSoup-p19) cells transformed with different plasmids were grown on plate with 25 μg/mL Kanamycin and 25 μg/mL Rifampicin for 48-72 h and then cultured in LB. GV3101(pSoup-p19) with different plasmids were mixed at a final OD_{600} of 0.8, and incubated for 3 h at dark before being co-infiltrated into one-month-old *Nicotiana benthamiana* leaves. The infiltrated plants were incubated in the dark for 24 h and then transferred the plants back to normal growing conditions for 48 h. The yellow fluorescent protein signals were excited at 514 nm and detected within 524-574 nm using a confocal laser scanning microscope (TCS SP8, Leica) with a HC PC APO 63X/1.40 oil CS2 lens.

Reference

1. Yu X-H, Wen X, Dong J, *et al.* Hydroxylation of HPPD facilitates its PUB11-mediated ubiquitination and degradation in response to oxidative stress in Arabidopsis. *Plant Commun* 2025; **6**:101521.

2. Zheng SQ, Palovcak E, Armache J-P, Verba KA, Cheng Y, Agard DA. MotionCor2: anisotropic correction of beam-induced motion for improved cryo-electron microscopy. *Nat Methods* 2017; **14**:331–332.

3. Punjani A, Rubinstein JL, Fleet DJ, Brubaker MA. cryoSPARC: algorithms for rapid unsupervised cryo-EM structure determination. *Nat Methods* 2017; **14**:290–296.

4. Jumper J, Evans R, Pritzel A, *et al.* Highly accurate protein structure prediction with AlphaFold. *Nature* 2021; **596**:583–589.

5. Emsley P, Lohkamp B, Scott WG, Cowtan K. Features and development of Coot. *Acta Crystallogr D Biol Crystallogr* 2010; **66**:486–501.

6. Adams PD, Afonine P V, Bunkóczi G, *et al.* PHENIX: a comprehensive Python-based system for macromolecular structure solution. *Acta Crystallogr D Biol Crystallogr* 2010; **66**:213–221.

7. Chen VB, Arendall WB 3rd, Headd JJ, *et al.* MolProbity: all-atom structure validation for macromolecular crystallography. *Acta Crystallogr D Biol Crystallogr* 2010; **66**:12–21.

8. Pettersen EF, Goddard TD, Huang CC, *et al.* UCSF Chimera--a visualization system for exploratory research and analysis. *J Comput Chem* 2004; **25**:1605–1612.

9. Bowers KJ, Chow E, Xu H, *et al.* Scalable algorithms for molecular dynamics simulations on commodity clusters. In: *Proceedings of the 2006 ACM/IEEE Conference on Supercomputing*.; 2006:84-es.

10. Jorgensen WL, Maxwell DS, Tirado-Rives J. Development and Testing of the OPLS All-Atom Force Field on Conformational Energetics and Properties of Organic Liquids. *J Am Chem Soc* 1996; **118**:11225–11236.

11. Lu C, Wu C, Ghoreishi D, *et al.* OPLS4: Improving Force Field Accuracy on Challenging Regimes of Chemical Space. *J Chem Theory Comput* 2021; **17**:4291–4300.

12. Humphrey W, Dalke A, Schulten K. VMD: visual molecular dynamics. *J Mol Graph* 1996; **14**:27-28,33-38.

Supplementary figures

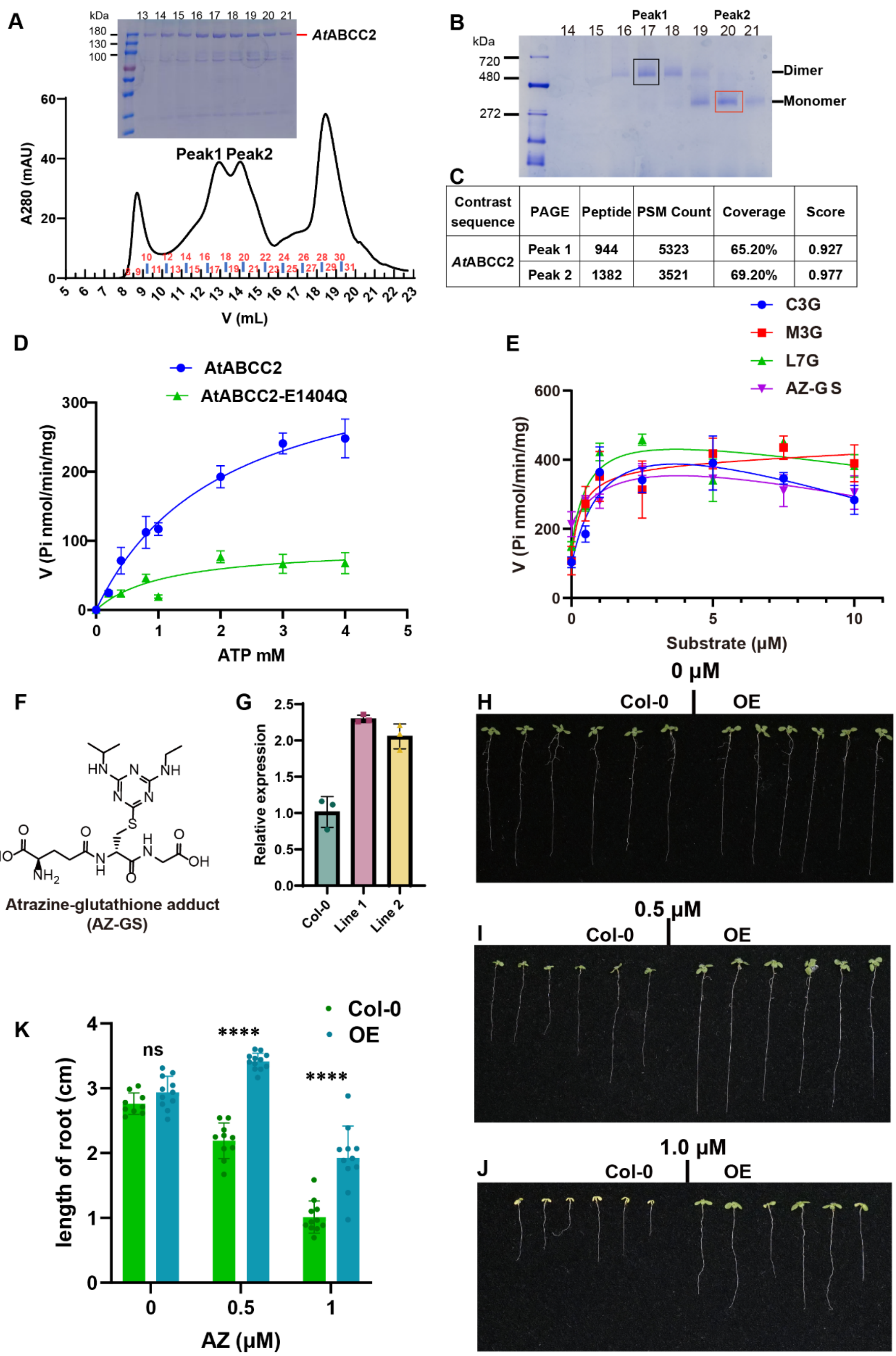

Supplementary Fig. S1 Biochemical characterization of purified *At*ABCC2. a Size-

exclusion chromatography profile and SDS-PAGE analysis of *At*ABCC2. Peak 1 and peak 2 correspond to dimer and monomer peak, respectively. **b** Blue native page analysis of peak 1 and peak 2 in **a**. Fraction numbers are indicated. The black rectangle indicates the pure dimer fraction and the red rectangle indicates the pure monomer fraction, which are used for following analyses. **c** Mass spectrometry analysis for peak 1 and peak 2 proteins. Both peaks correspond to *At*ABCC2. **d** The ATPase activities of wild type (WT) and E1404Q mutant of *At*ABCC2. **e** The substrate-stimulated ATPase activity of *At*ABCC2 upon the addition of cyanidin 3-O-glucoside (C3G), malvidin 3-O-glucoside (M3G), luteolin 7-O-glucoside (L7G), and atrazine-glutathione conjugate (AZ-GS). All data points represent means (n=3)±standard deviation (SD). **f** Chemical structures of atrazine-glutathione adduct. **g** RT-qPCR results to detect the expression level of OE lines. Compared to Col-0, the OE lines displayed about two-fold increase of expression. **h-j** Herbicide resistance of Col-0 and *At*ABCC2-overexpression (OE) *Arabidopsis*. Root lengths for the plants were compared. Concentrations of atrazine treating the plants are indicated above. **k** Quantitative measurement of the root length. Data are shown as mean±S.D. Two-tailed Student's t test: ****p < 0.0001, ns, not significant.

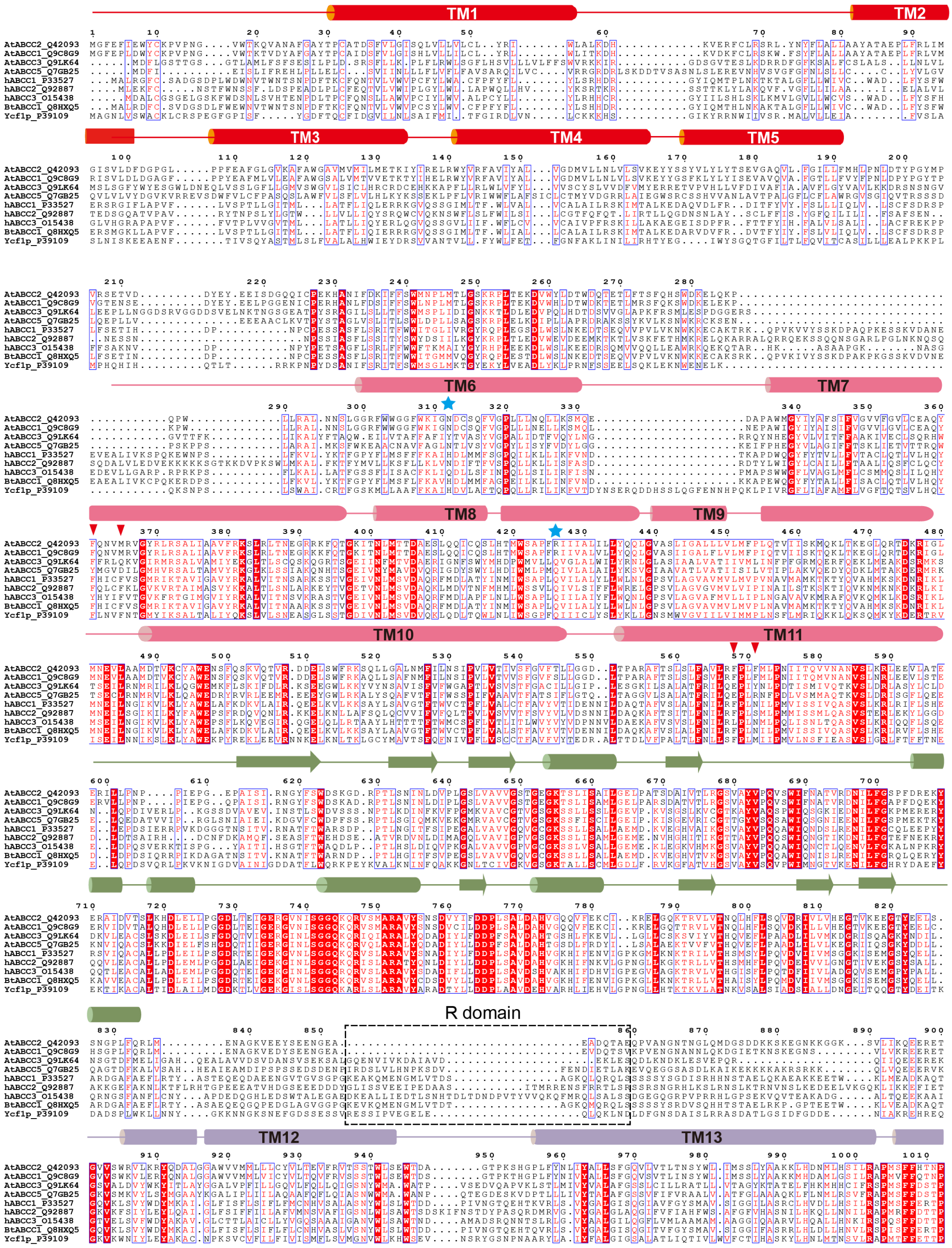
TM1
TM2
TM3
TM4
TM5
TM6
TM7
TM8
TM9
TM10
TM11
R domain
TM12
TM13
AtABCC2_Q42093
AtABCC1_Q9C8G9
AtABCC3_Q9LK64
AtABCC5_Q7GB25
hABCC1_P33527
hABCC2_Q92887
hABCC3_O15438
BtABCC1_Q8HXQ5
Ycf1p_P39109

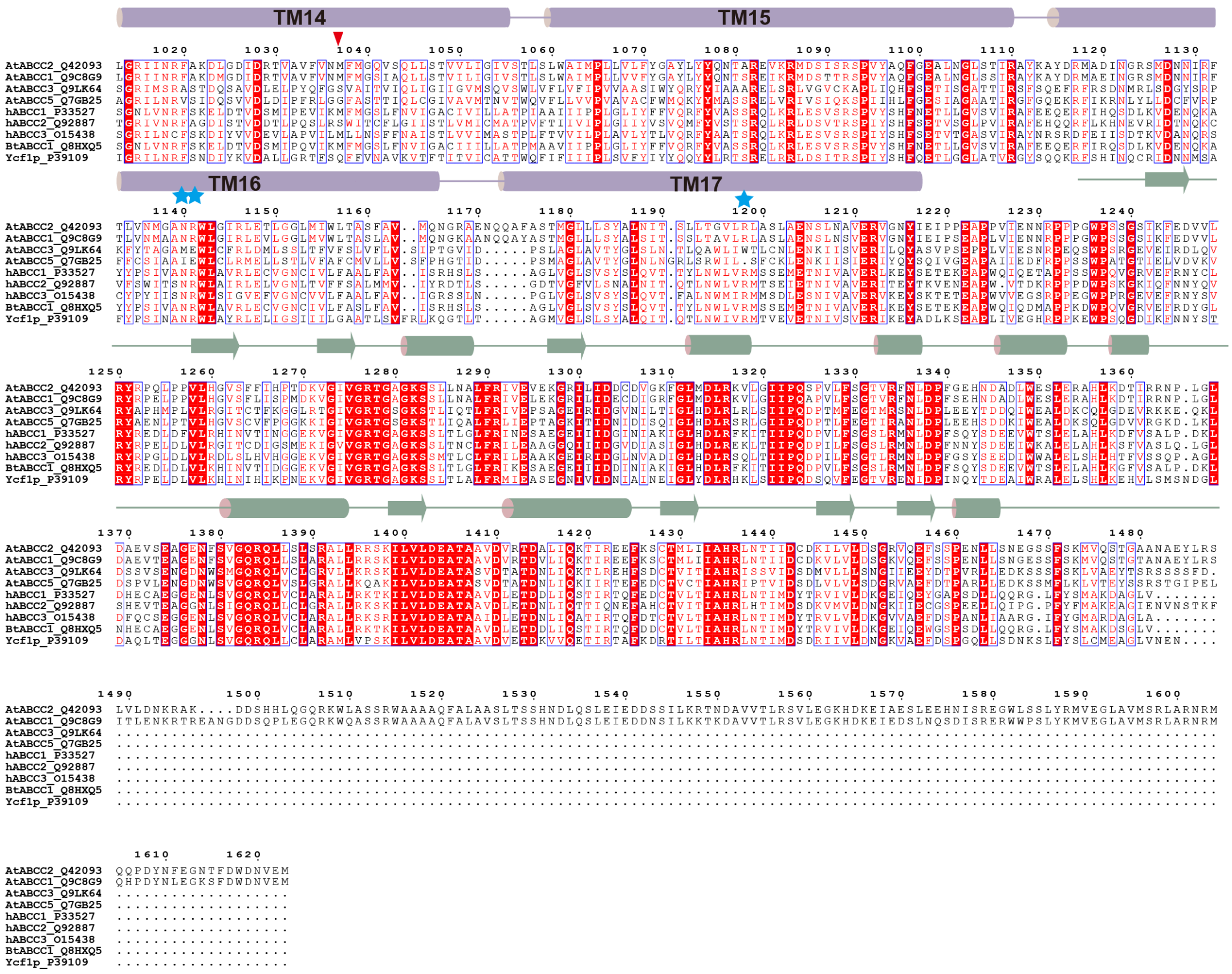

Supplementary Fig. S2 Sequence alignment of ABCC transporters from human, yeast, bovine, and Arabidopsis. The secondary structures are indicated. Residues participating in polar interaction with substrate are indicated with blue stars. Residues mediating hydrophobic interactions with substrate are indicated with red triangles. The dashed rectangle indicates the putative R domain.

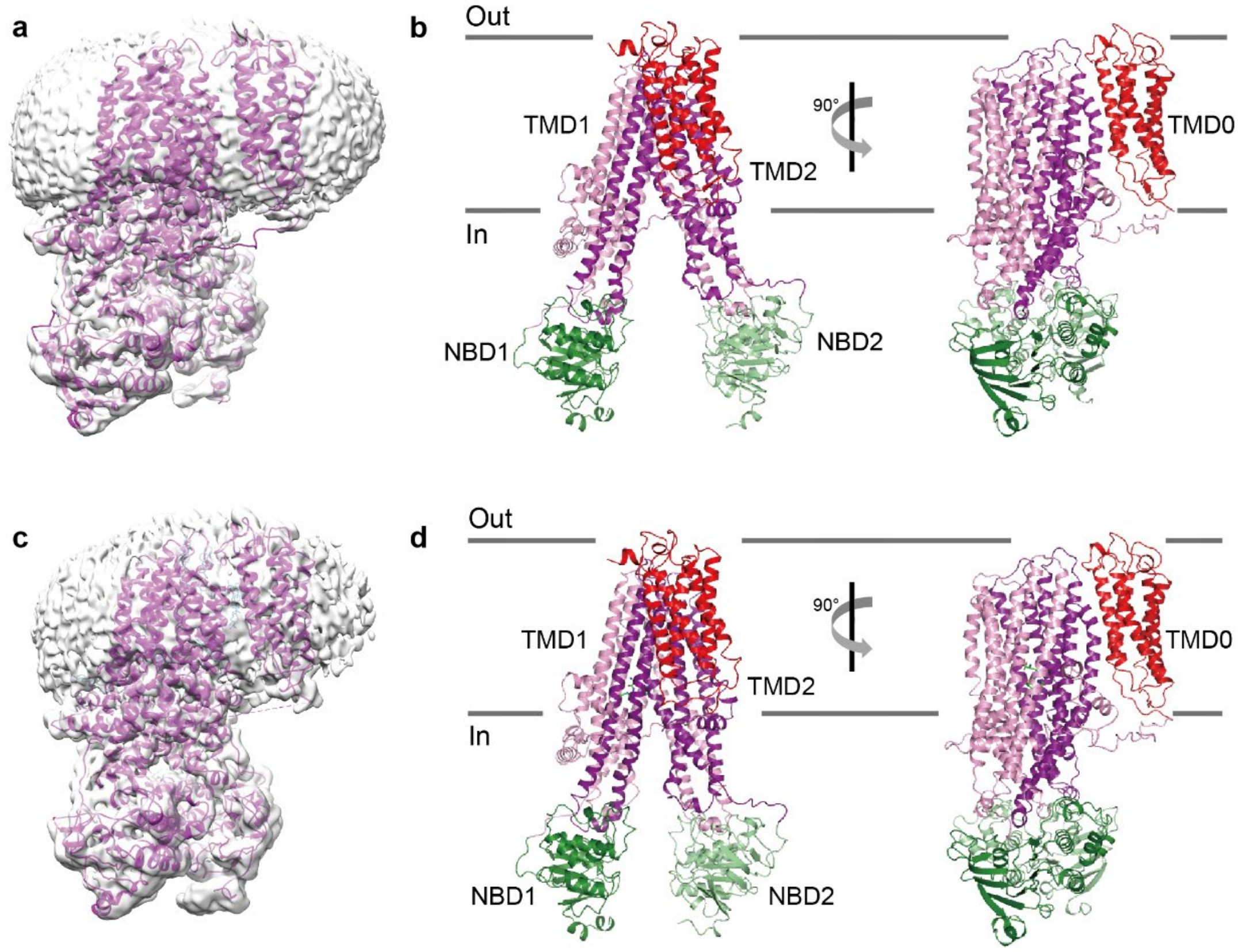

Supplementary Fig. S3 Structures of *At*ABCC2 in apo and AZ-GS bound states. **a** Cryo-EM map of *At*ABCC2 in apo state at 2.9 Å resolution. Continuous density can be observed for the different domains. **b** Cartoon representation of the apo *At*ABCC2. TMD0 is colored in red, TMD1 in pink, TMD2 in purple, NBD1 in deep green, and NBD2 in light green. The membrane is indicated with the gray lines. **c** Cryo-EM map of *At*ABCC2 in AZ-GS bound state at 3.4 Å resolution. **d** Cartoon representation of the AZ-GS bound *At*ABCC2.

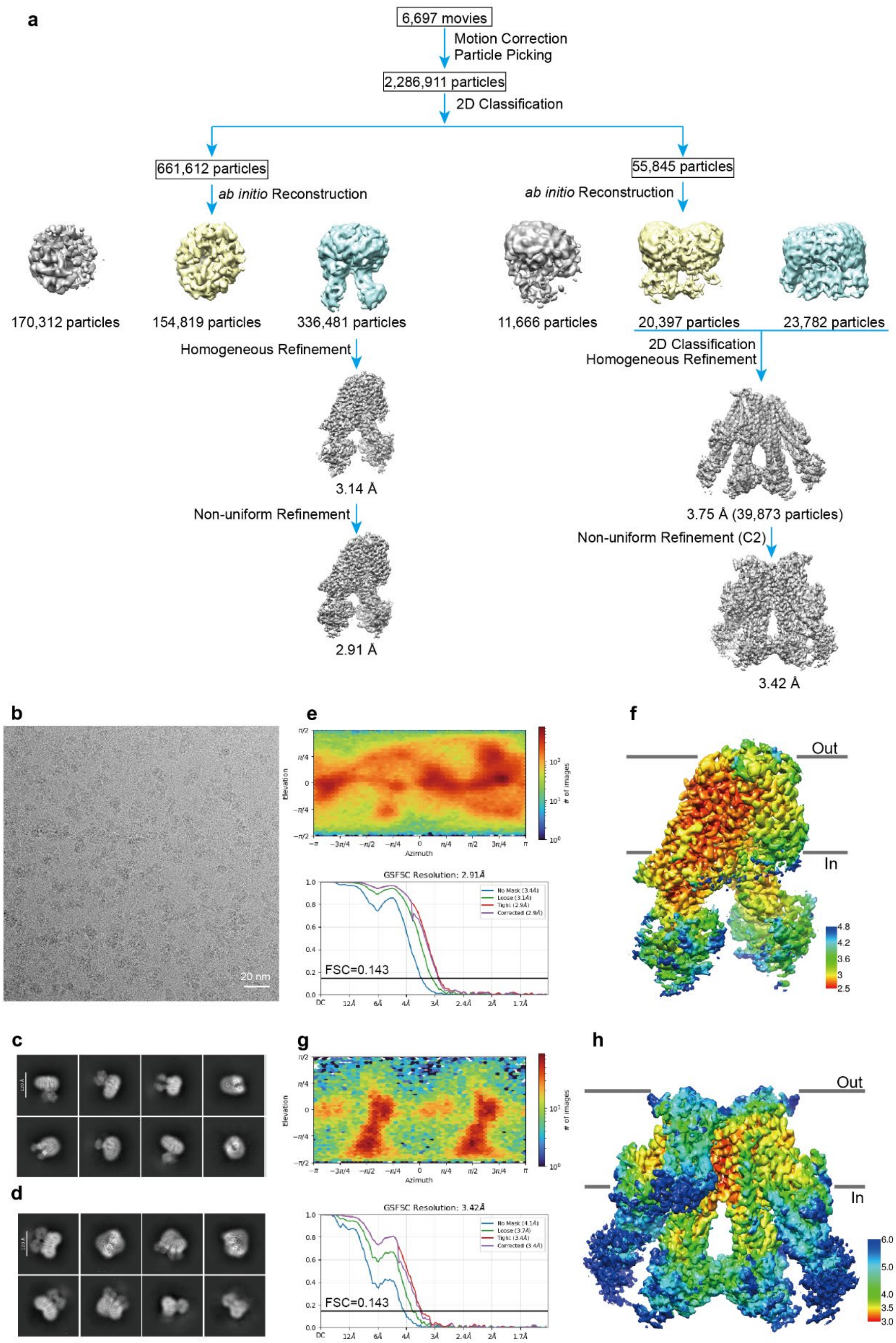

Supplementary Fig. S4 Cryo-EM analysis of apo and dimeric *At*ABCC2. a Flowchart of the cryo-EM data processing by cryoSPARC 3.1. **b** Representative raw micrograph. Scale bar is added. **c-d** Representative 2D averages of apo and dimeric

*At*ABCC2.**e** Fourier shell correlation (FSC) curves and Euler angle distributions of the classified particles used for the final 3D refinement of the apo *At*ABCC2. **f** Local resolution of the map for apo *At*ABCC2 estimated by cryoSPARC 3.1. **g** Fourier shell correlation (FSC) curves and Euler angle distributions of the classified particles used for the final 3D refinement of the dimeric *At*ABCC2. **h** Local resolution of the map for dimeric *At*ABCC2 estimated by cryoSPARC 3.1.

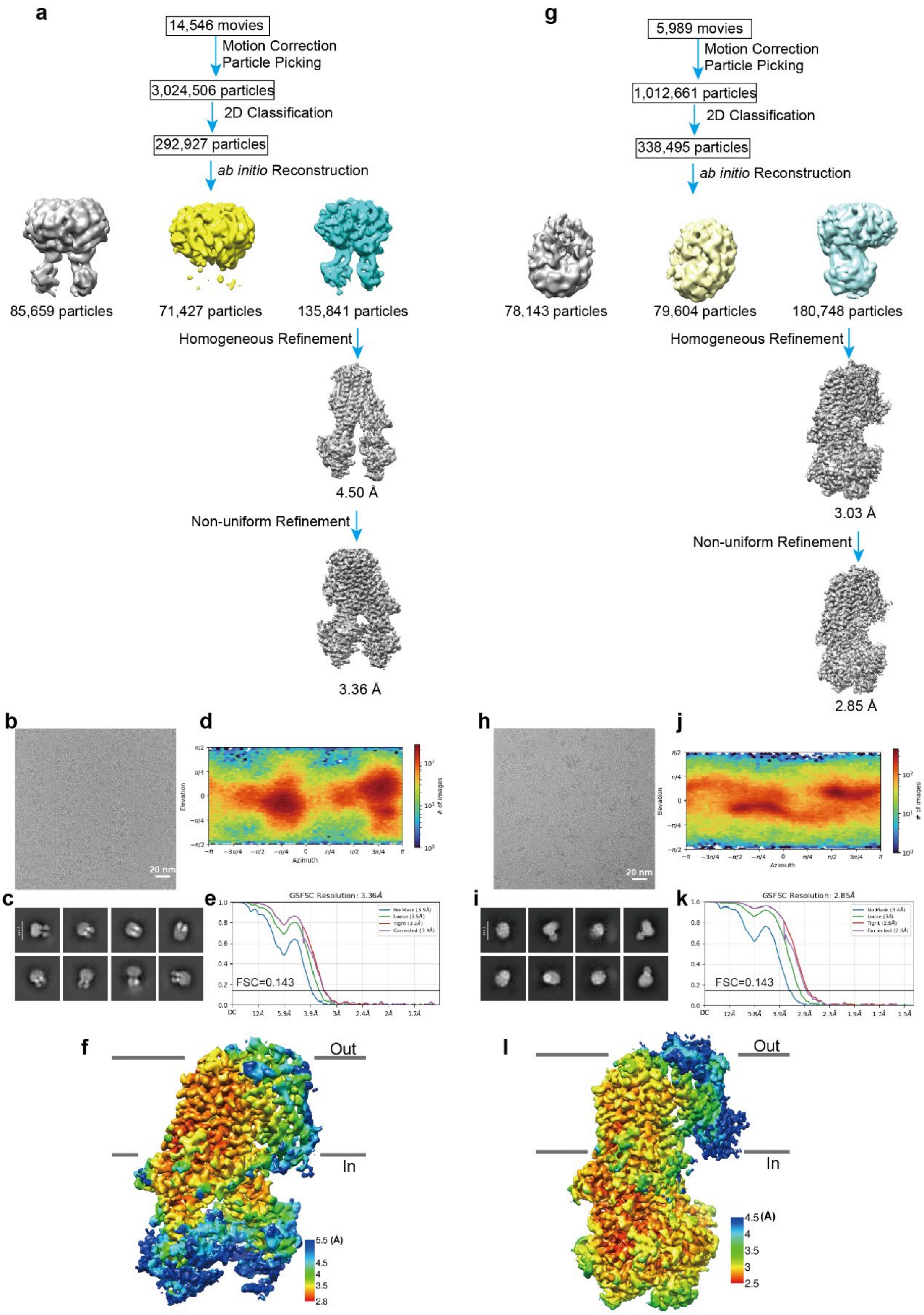

Supplementary Fig. S5 Cryo-EM analysis of AZ-GS bound and closed *At*ABCC2. **a** Flowchart of the cryo-EM data processing for AZ-GS bound *At*ABCC2 by cryoSPARC 3.1. **b** Representative raw micrograph for AZ-GS bound structure. Scale bar was added. **c** Representative 2D averages of AZ-GS bound *At*ABCC2. **d-e** Euler angle distributions of the classified particles (d) and Fourier shell correlation (FSC)

curves (e) used for the final 3D refinement of the AZ-GS bound *At*ABCC2. **f** Local resolution of the map for AZ-GS bound *At*ABCC2 estimated by cryoSPARC 3.1. **g** Flowchart of the cryo-EM data processing for closed *At*ABCC2 by cryoSPARC 3.1. **h** Representative raw micrograph for AZ-GS bound structure. Scale bar was added. **i** Representative 2D averages of closed *At*ABCC2. **j-k** Euler angle distributions of the classified particles (j) and Fourier shell correlation (FSC) curves (k) used for the final 3D refinement of the closed *At*ABCC2. **l** Local resolution of the map for closed *At*ABCC2 estimated by cryoSPARC 3.1.

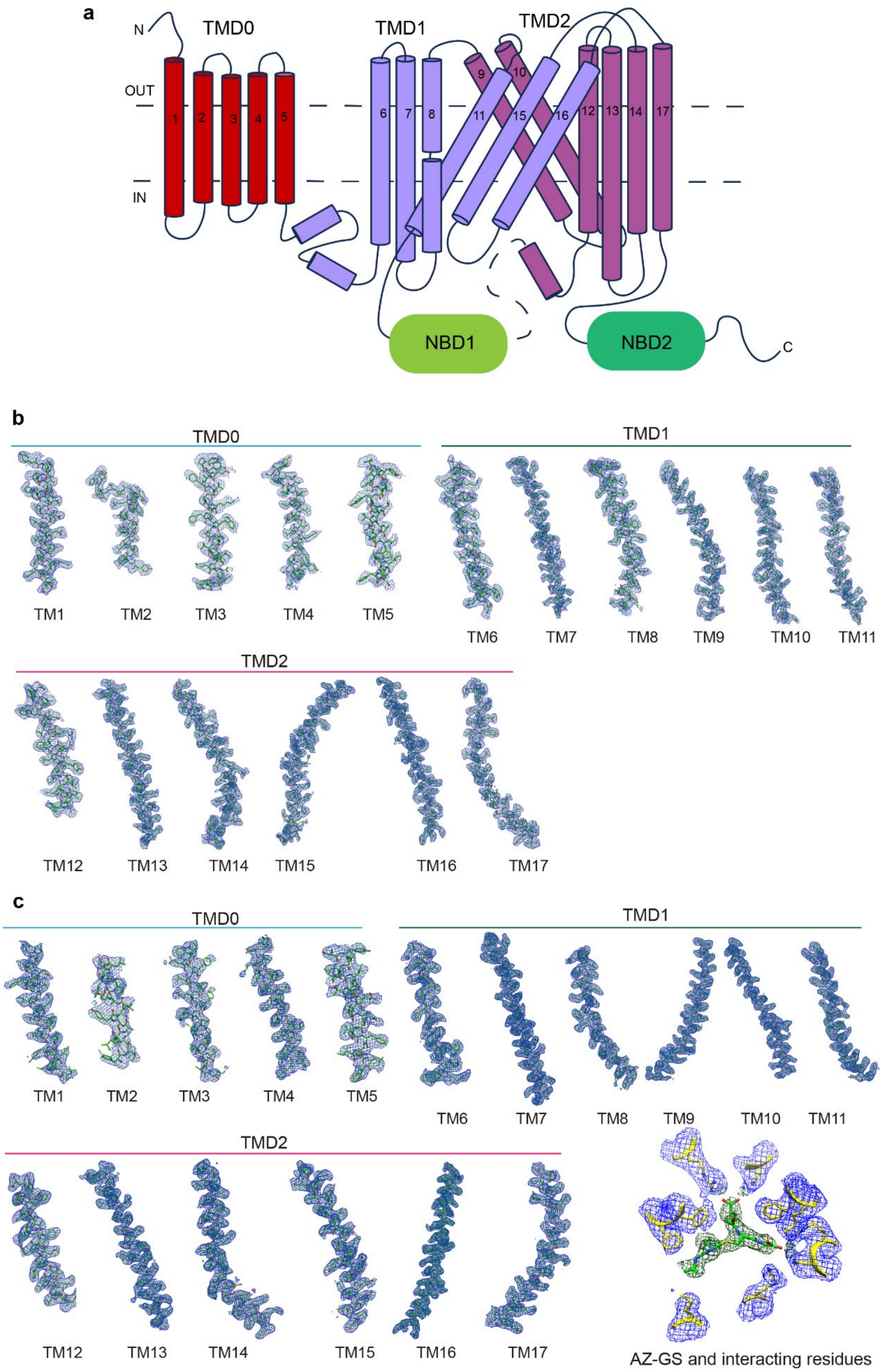
a
N
TMD0
TMD1
TMD2
OUT
IN
1
2
3
4
5
6
7
8
9
10
11
15
16
12
13
14
17
NBD1
NBD2
C
b
TMD0
TMD1
TM1
TM2
TM3
TM4
TM5
TM6
TM7
TM8
TM9
TM10
TM11
TMD2
TM12
TM13
TM14
TM15
TM16
TM17
c
TMD0
TMD1
TM1
TM2
TM3
TM4
TM5
TM6
TM7
TM8
TM9
TM10
TM11
TMD2
TM12
TM13
TM14
TM15
TM16
TM17
AZ-GS and interacting residues

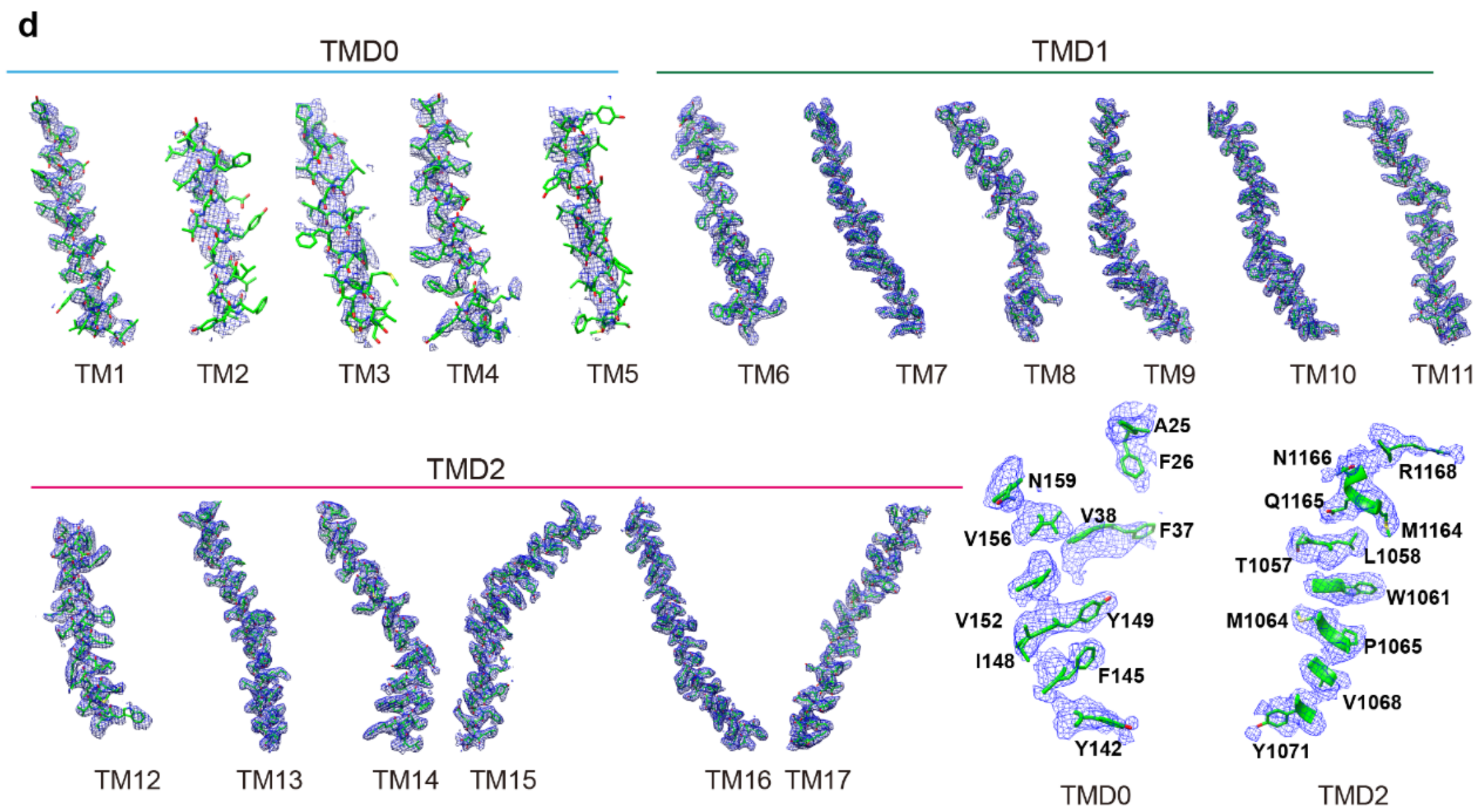

Supplementary Fig. S6 Structure of *At*ABCC2 in apo, AZ-GS bound, and closed states. **a** Topological diagram of *At*ABCC2. **b** Cryo-EM densities of representative transmembrane regions of apo *At*ABCC2. Contour levels are set at 5σ. All the density maps are carved at a distance of 2 Å. **c** Cryo-EM densities of representative transmembrane regions of AZ-GS bound *At*ABCC2. Densities for AZ-GS and its interacting residues were also included. **d** Cryo-EM densities of representative transmembrane regions of closed *At*ABCC2. The densities for residues in the TMD0 and TMD2 interface were also shown, and the contour level of their maps was set at 4σ.

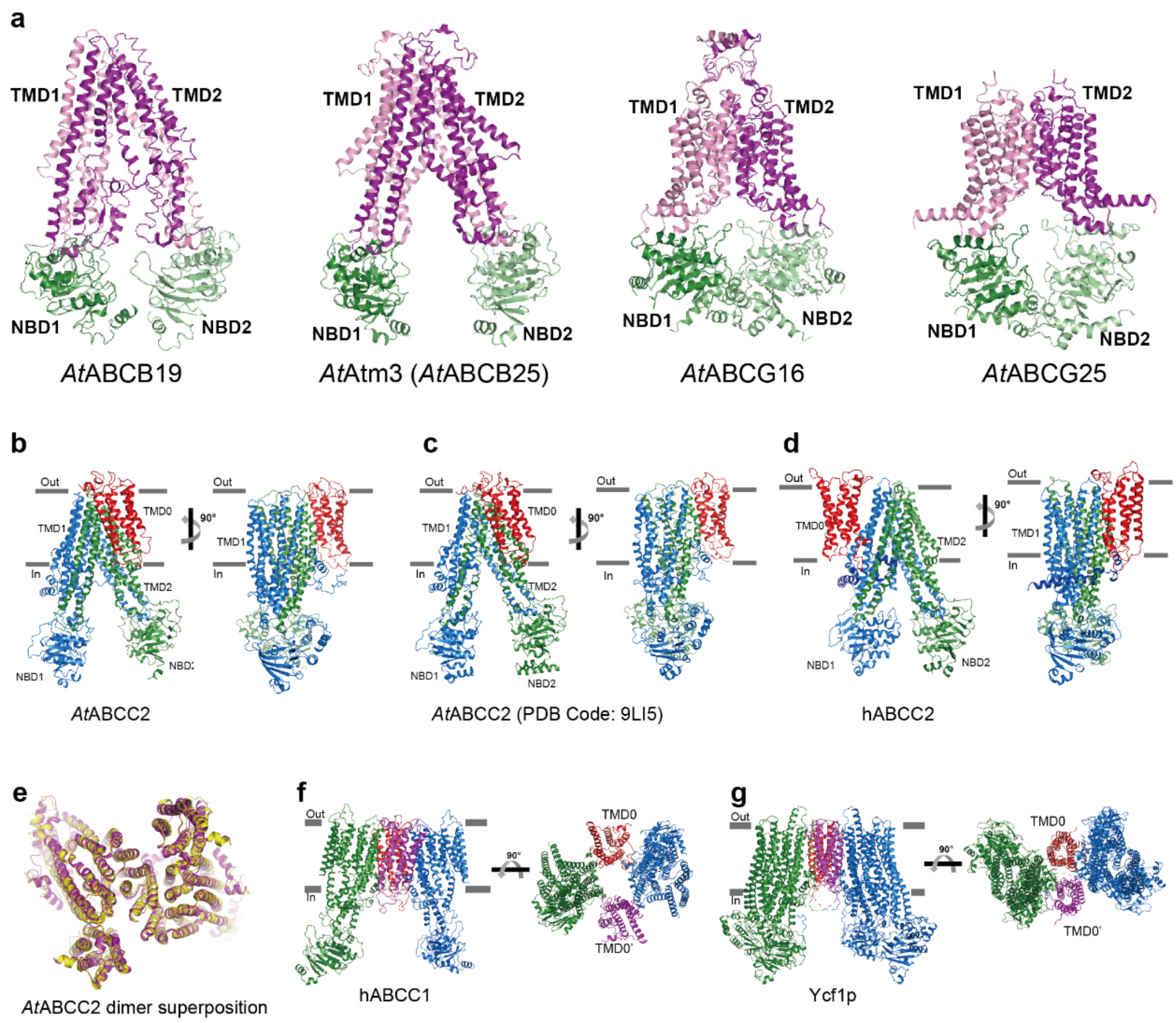

Supplementary Fig. S7 a Structural comparison of plant ABC transporters. Apo states of *At*ABCB19, *At*Atm3, *At*ABCG16, and *At*ABCG25 are compared. PDB codes are included. **b** Overall structure of *At*ABCC2 in this study. **c** The reported *At*ABCC2 structure (PDB Code: 9LI5). TMD0 is shown in red, TMD1-NBD1 in blue, and TMD2-NBD2 in green. For comparison, the structures are aligned first. **d** Overall structure of hABCC2. **e** Superposition of *At*ABCC2 dimer. Top view. Magenta, structure in this manuscript; yellow, reported *At*ABCC2 dimer (PDB Code: 9LI7). **f-g** Overall structure of hABCC1 dimer (f) and Ycf1p dimer (g). The membrane is indicated with the gray lines. Structural domains are indicated.

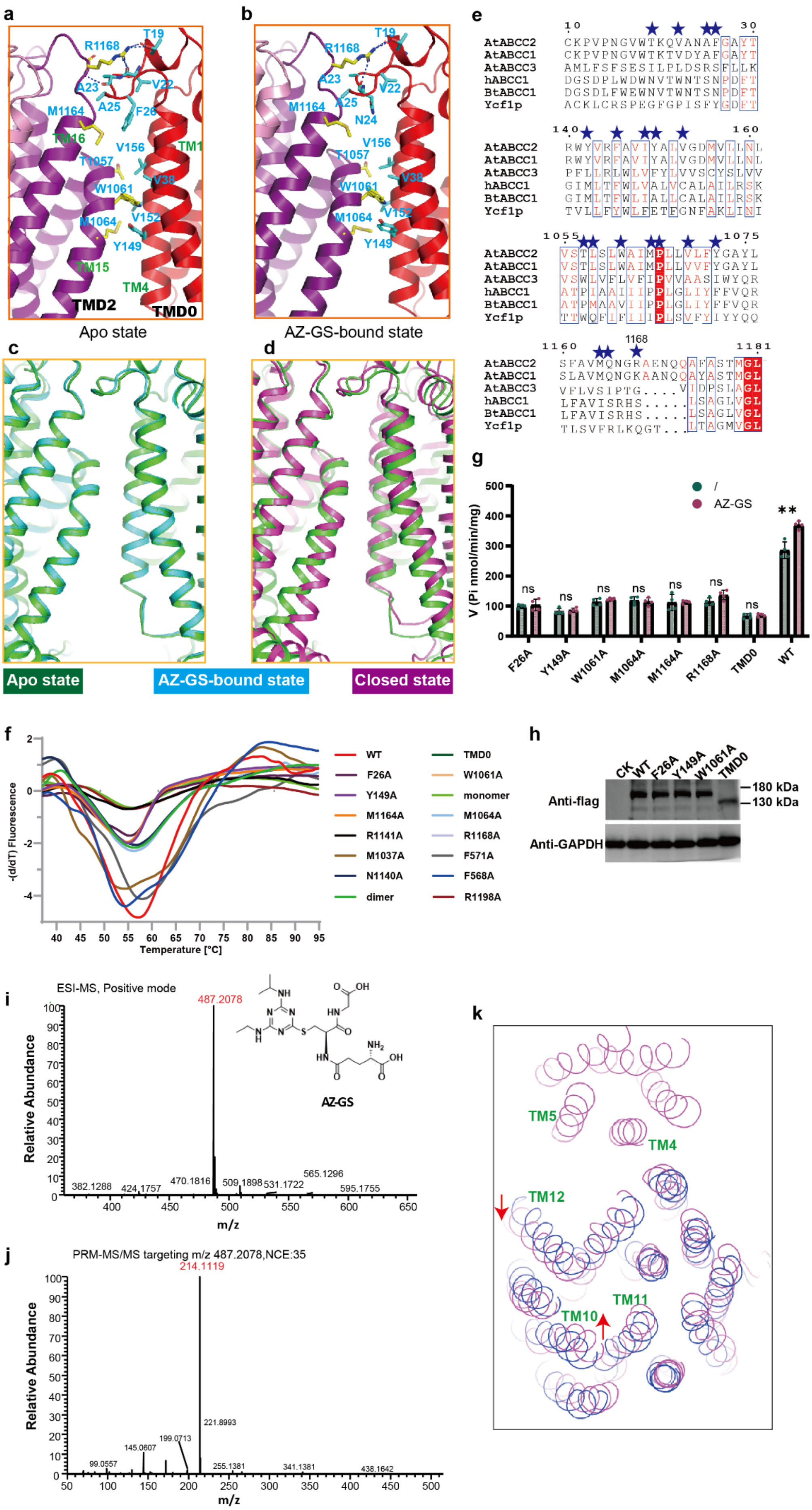

a
R1168
T19
A23
V22
A25
F26
M1164
TM16
V156
TM1
T1057
V38
W1061
V152
M1064
Y149
TM15
TM4
TMD2
TMD0
Apo state
b
AZ-GS-bound state
N24
e
AtABCC2
AtABCC1
AtABCC3
hABCC1
BtABCC1
Ycf1p
c
d
Apo state
AZ-GS-bound state
Closed state
g
V (Pi nmol/min/mg)
AZ-GS
ns
**
F26A
Y149A
W1061A
M1064A
M1164A
R1168A
TMD0
WT
f
-(dI/dT) Fluorescence
Temperature [°C]
WT
F26A
Y149A
M1164A
R1141A
M1037A
N1140A
dimer
TMD0
W1061A
monomer
M1064A
R1168A
F571A
F568A
R1198A
h
CK
Anti-flag
Anti-GAPDH
180 kDa
130 kDa
i
ESI-MS, Positive mode
487.2078
Relative Abundance
AZ-GS
382.1288
424.1757
470.1816
509.1898
531.1722
565.1296
595.1755
m/z
j
PRM-MS/MS targeting m/z 487.2078,NCE:35
214.1119
221.8993
199.0713
145.0607
99.0557
255.1381
341.1381
438.1642
k
TM5
TM4
TM12
TM11
TM10

Supplementary Fig. S8 The interface between TMD0 and TMD2 is crucial for function. **a-b** The interactions between TMD0 and TMD2 of *At*ABCC2 in different functional states. Interacting residues are shown as sticks. Dashed lines indicate hydrogen bonds. **c-d** Overall structural arrangement comparison of TMD0 and TMD2 of *At*ABCC2 in different functional states. **e** Sequence alignment of ABCC transporters from human, yeast, bovine (*Bt*), and *Arabidopsis (At)*. Key residues mediating the interaction between TMD0 and TMD2 are indicated with a star. **f** Melting temperature (T_m) value measurement for *At*ABCC2 and its mutants. **g** ATPase activity stimulated by AZ-GS of interaction residue mutations. **h** Expression level of the mutants detected by western blot. TMD0 indicates the TMD0 deletion mutant. The mutants and WT protein display comparable expression level. **i** Positive-ion ESI-MS spectrum of AZ–GS. The diagnostic ion at m/z 487.2078 is highlighted. **j** PRM-MS/MS spectrum of AZ–GS acquired in positive ion mode. Fragmentation of the precursor ion (m/z 487.2078; HCD, NCE 35) generated a dominant product ion at m/z 214.1119 (highlighted in red), which was selected as the quantifier ion for PRM-based quantification. **k** Comparison of the WT and TMD0 deletion mutant models after 200 ns simulations. After 200 ns simulation, the system reached stable. The last frame was used for comparison. The WT model was shown in purple, while the TMD0 deletion mutant shown in blue.

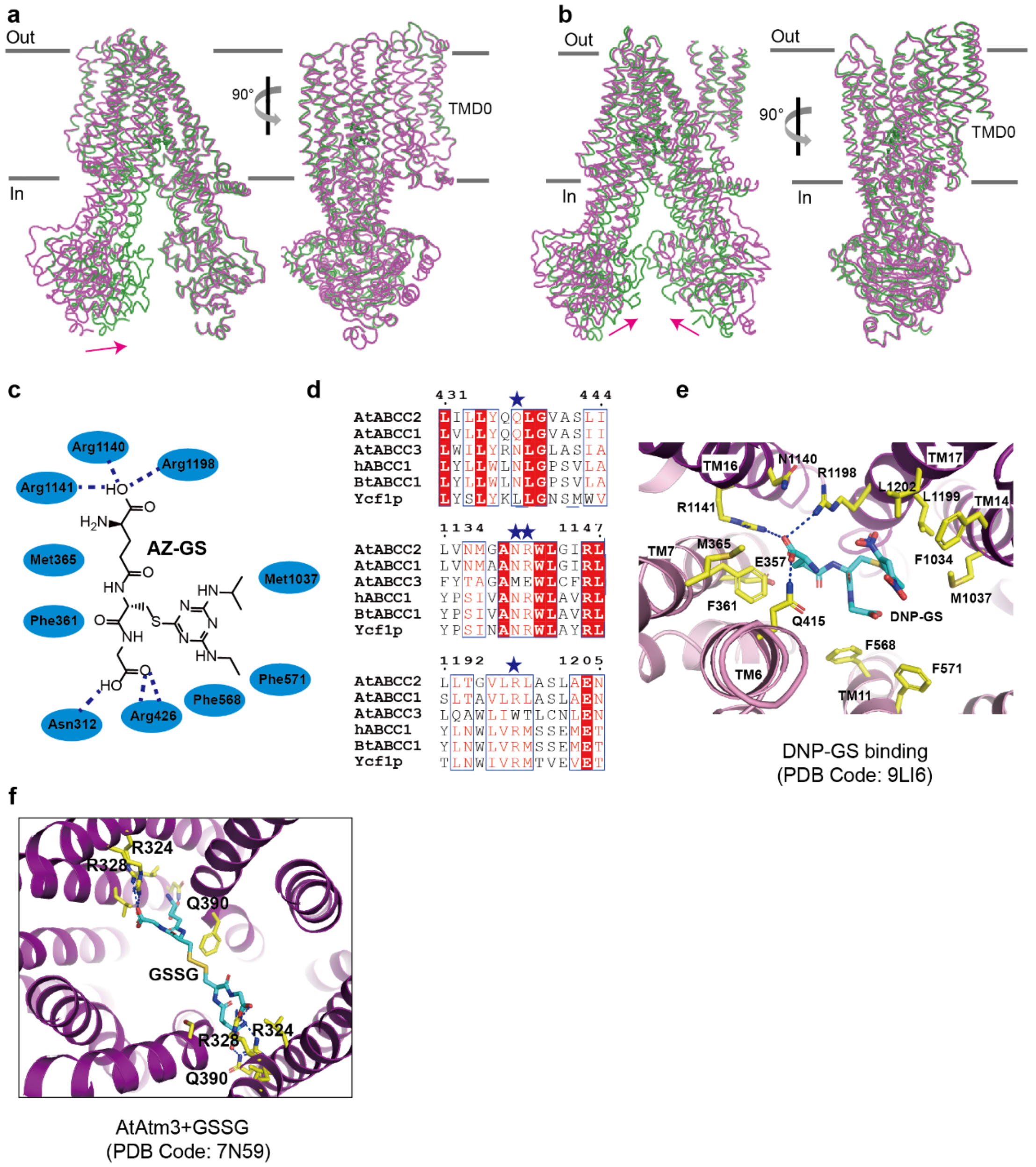

Supplementary Fig. S9 Structural comparison of apo (magenta) and substrate-bound (green) state of *At*ABCC2 (**a**) and bovine Mrp1 (**b**). The membrane is indicated with the gray lines. Red arrow indicates the translocation of NBD in substrate bound structures. AZ-GS was shown as ball-and-stick model. Bovine Mrp1 (PDB Code: 5UJA) was bound with leukotriene C4 (ball-and-stick model). **c** Schematic diagram of AZ-GS binding. **d** Sequence alignment of ABCC transporters from human, yeast, bovine, and *Arabidopsis*. Key residues mediating the interaction between protein and AZ-GS are indicated with stars. **e** Binding of DNP-GS in *At*ABCC2 (PDB Code: 9LI6). DNP-GS is shown in cyan, binding residues in yellow. Dashed lines indicate polar contacts. **f**

Binding of GSSG in *At*Atm3. GSSG was shown in cyan stick; binding residues were shown as yellow stick.

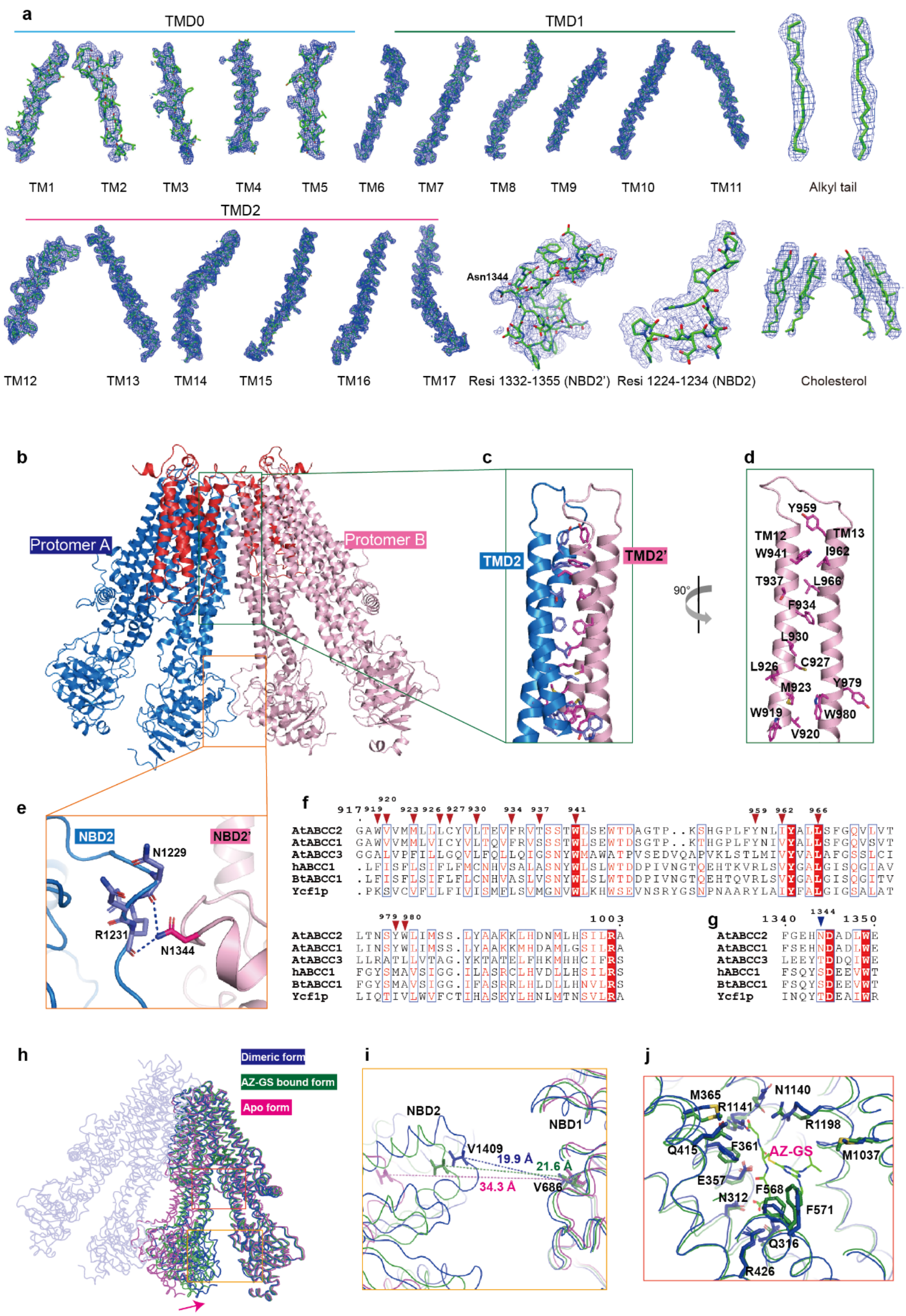

Supplementary Fig. S10 Structure of *At*ABCC2 in dimeric state. **a** Cryo-EM densities of representative regions of dimeric *At*ABCC2. Contour levels are set at 5σ for transmembrane helices and NBD regions. All the density maps are carved at a distance of 2 Å. Cryo-EM density for the cholesterol molecules and alkyl tails around

the dimer interface was also shown. **b-d** Interactions between TMD2 and TMD2'. Residues participating hydrophobic interactions are shown as sticks. **e** Interactions between NBD2 and NBD2'. Dashed lines indicate hydrogen bonds. **f-g** Sequence alignment of ABCC transporters from human, yeast, bovine, and Arabidopsis. Residues mediating hydrophobic (f) and polar (g) contacts are indicated with triangles. **h** Overall comparison of dimeric and AZ-GS bound *At*ABCC2. Red arrow indicates the translocation of NBD2 in dimeric *At*ABCC2. **i** Distance measurement between two NBDs. Cα atoms of Residues V686 and V1409 were taken as references. The distances are labeled and colored according to the structures. **j** Residue comparison of the AZ-GS binding pocket between substrate-bound and dimeric state. Surrounding residues are shown as sticks and colored according the cartoon color. AZ-GS is shown as green stick.

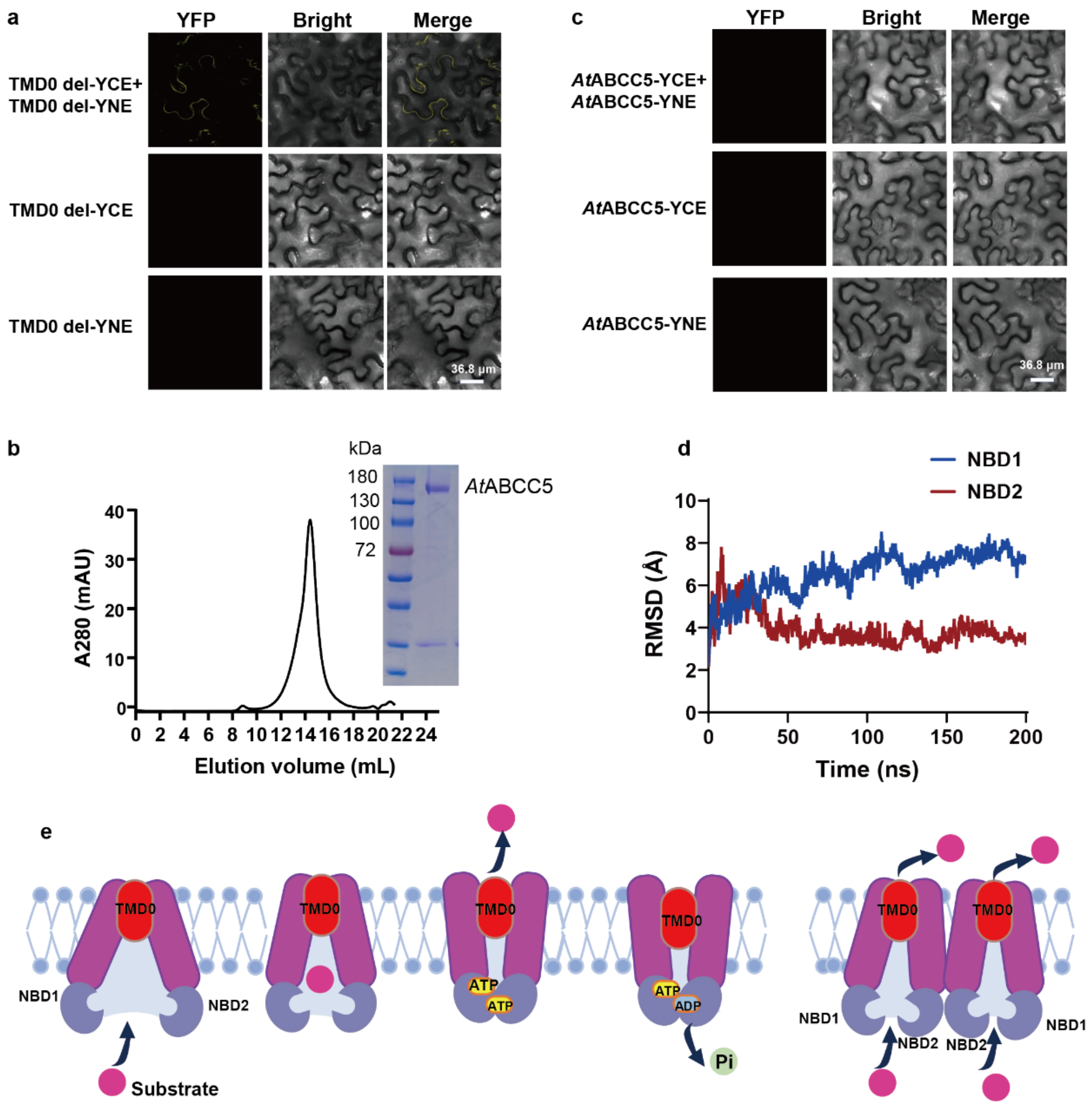

Supplementary Fig. S11 Functional characterization of dimeric *At*ABCC2. a BiFC results for the TMD0 deleted *At*ABCC2. **b** SEC profile and SDS-PAGE analysis of *At*ABCC5. Only the monomer peak was observed in the chromatograph. **c** BiFC results using split YFP to detect the fluorescence of intact YFP formed by *At*ABCC5. Scale bar = 36.8 μm. **d** RMSD results of NBD1 and NBD2 during the 200 ns MD simulations. **e** Working model of substrate export by *At*ABCC2. TMD0 plays a critical role in coordinating the function of the transmembrane transport process. Both monomer and dimer participate in xenobiotics export in *Arabidopsis*. TMD2 and NBD2 mediate dimerization, rather than TMD0. But dimeric *At*ABCC2 is in a suppressed functional state. Its ATPase activity and substrate export activity are lower than monomer due to the limited mobility of NBD2.

Supplementary Table S1 | Cryo-EM data collection, refinement, and validation statistics.

	Apo *At*ABCC2	Dimeric *At*ABCC2	AZ-GS bound *At*ABCC2	Closed *At*ABCC2
**EMDB code**	EMD-63864	EMD-63865	EMD-63866	EMD-65915
**PDB code**	9U56	9U57	9U58	9WET
**Data collection and processing**				
Magnification	165,000	165,000	165,000	165,000
Voltage (kV)	300	300	300	300
Camera	Falcon4	Falcon4	Falcon4	Falcon4
Electron exposure dose ($e^-/Å^2$)	49.72	49.72	49.79	50.10
Defocus range (μM)	-2.5 ~ -1.2	-2.5 ~ -1.2	-2.5 ~ -1.2	-2.5 ~ -1.2
Pixel size (Å)	0.75	0.75	0.74	
Symmetry imposed	C1	C2	C1	C1
Initial particles	2,286,911	2,286,911	3,024,506	1,012,661
Final particles	336,481	39,873	135,841	180,748
Map resolution (Å)	2.9	3.4	3.4	2.9
FSC threshold	0.143	0.143	0.143	0.143
B factor sharpening applied to the map ($Å^2$)	-121.2	-108.2	-124.4	-104.9
**Refinement**				
Model resolution (Å)	3.0	3.7	3.5	2.9
FSC threshold	0.5	0.5	0.5	0.5
Molprobity score	1.9	2.5	2.3	2.4
Model composition				
Nonhydrogen atoms	10915	21830	10962	11529
Protein residues	1379	2758	1379	1417
ligands	8 CLR	2 Hexadecane, 10 CLR	1 AZ-GS, 7 CLR	2 ATP, 6 CLR, 2 Mg^{2+}
Mean B factors ($Å^2$)				
Protein	103.58	169.36	84.02	75.42
Ligand	-	-	41.33	77.88
R.m.s. deviations				
Bond lengths (Å)	0.005	0.006	0.014	0.003
Bond angles (°)	1.041	1.109	1.468	0.697
Ramachandran plot				

Favored (%)	97.09	95.85	91.70	96.95
Allowed (%)	2.91	4.15	8.30	3.05
Outlier (%)	0.00	0.00	0.00	0.00

Supplementary Movie S1 200 ns MD simulations for the WT *At*ABCC2 in apo state. The TMD0 is shown in red, TMD1 in pink, TMD2 in purple, NBD1 in dark green, and NBD2 in green.

Supplementary Movie S2 200 ns MD simulations for the TMD0 deleted *At*ABCC2 in apo state. The TMD1 is shown in pink, TMD2 in purple, NBD1 in limegreen, and NBD2 in green.

Supplementary Movie S3 200 ns MD simulations for the dimeric *At*ABCC2. For each protomer, The TMD0 is shown in red, TMD1 in pink, TMD2 in purple, NBD1 in dark green, and NBD2 in green.
