# Supplementary material for: Molecular basis of Arabidopsis ABCC2 in plant detoxification
Source: Cell Discov. 2026 Aug 4;12:57. doi: 10.1038/s41421-026-00919-z (PMC13439234; doi:10.1038/s41421-026-00919-z)
Supplement: Supplementary file 2 [file 41421_2026_919_MOESM2_ESM.pdf]

Full wwPDB EM Validation Report (i)

Aug 21, 2025 – 01:13 PM JST

PDB ID	:	9U57 / pdb_00009u57
EMDB ID	:	EMD-63865
Title	:	Cryo-EM structure of dimeric AtABCC2
Deposited on	:	2025-03-20
Resolution	:	3.42 Å(reported)

This wwPDB validation report is for manuscript review

For Manuscript Review

This is a Full wwPDB EM Validation Report.

This report is produced by the wwPDB biocuration pipeline after annotation of the structure.

We welcome your comments at *validation@mail.wwpdb.org*
A user guide is available at
https://www.wwpdb.org/validation/2017/EMValidationReportHelp
with specific help available everywhere you see the (i) symbol.

The types of validation reports are described at
http://www.wwpdb.org/validation/2017/FAQs#types.

The following versions of software and data (see references (i)) were used in the production of this report:

EMDB validation analysis	:	0.0.1.dev126
Mogul	:	1.8.5 (274361), CSD as541be (2020)
MolProbity	:	4-5-2 with Phenix2.0rc1
buster-report	:	1.1.7 (2018)
Percentile statistics	:	20231227.v01 (using entries in the PDB archive December 27th 2023)
MapQ	:	1.9.13
Ideal geometry (proteins)	:	Engh & Huber (2001)
Ideal geometry (DNA, RNA)	:	Parkinson et al. (1996)
Validation Pipeline (wwPDB-VP)	:	2.45.1

1 Overall quality at a glance (i)

The following experimental techniques were used to determine the structure:
ELECTRON MICROSCOPY

The reported resolution of this entry is 3.42 Å.

Percentile scores (ranging between 0-100) for global validation metrics of the entry are shown in the following graphic. The table shows the number of entries on which the scores are based.

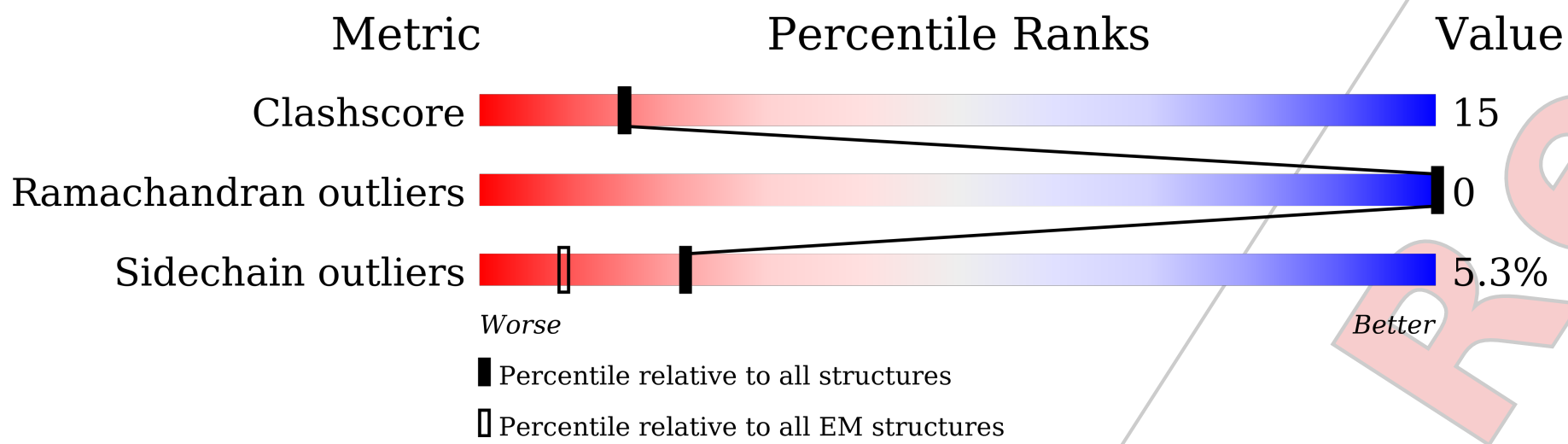

Metric	Whole archive (#Entries)	EM structures (#Entries)
Clashscore	210492	15764
Ramachandran outliers	207382	16835
Sidechain outliers	206894	16415

The table below summarises the geometric issues observed across the polymeric chains and their fit to the map. The red, orange, yellow and green segments of the bar indicate the fraction of residues that contain outliers for >=3, 2, 1 and 0 types of geometric quality criteria respectively. A grey segment represents the fraction of residues that are not modelled. The numeric value for each fraction is indicated below the corresponding segment, with a dot representing fractions <=5% The upper red bar (where present) indicates the fraction of residues that have poor fit to the EM map (all-atom inclusion < 40%). The numeric value is given above the bar.

Mol	Chain	Length	Quality of chain
1	A	1464	22%<br>62% 29% • 6%
1	B	1464	23%<br>64% 29% • 6%

The following table lists non-polymeric compounds, carbohydrate monomers and non-standard residues in protein, DNA, RNA chains that are outliers for geometric or electron-density-fit criteria:

Mol	Type	Chain	Res	Chirality	Geometry	Clashes	Electron density
3	R16	B	1502	-	-	X	-

For Manuscript Review

2 Entry composition (i)

There are 3 unique types of molecules in this entry. The entry contains 22142 atoms, of which 0 are hydrogens and 0 are deuteriums.

In the tables below, the AltConf column contains the number of residues with at least one atom in alternate conformation and the Trace column contains the number of residues modelled with at most 2 atoms.

- Molecule 1 is a protein called ABC transporter C family member 2.

Mol	Chain	Residues	Atoms					AltConf	Trace
			Total	C	N	O	S		
1	A	1379	10915	7068	1839	1956	52	0	0
1	B	1379	10915	7068	1839	1956	52	0	0

- Molecule 2 is CHOLESTEROL (CCD ID: CLR) (formula: $C_{27}H_{46}O$).

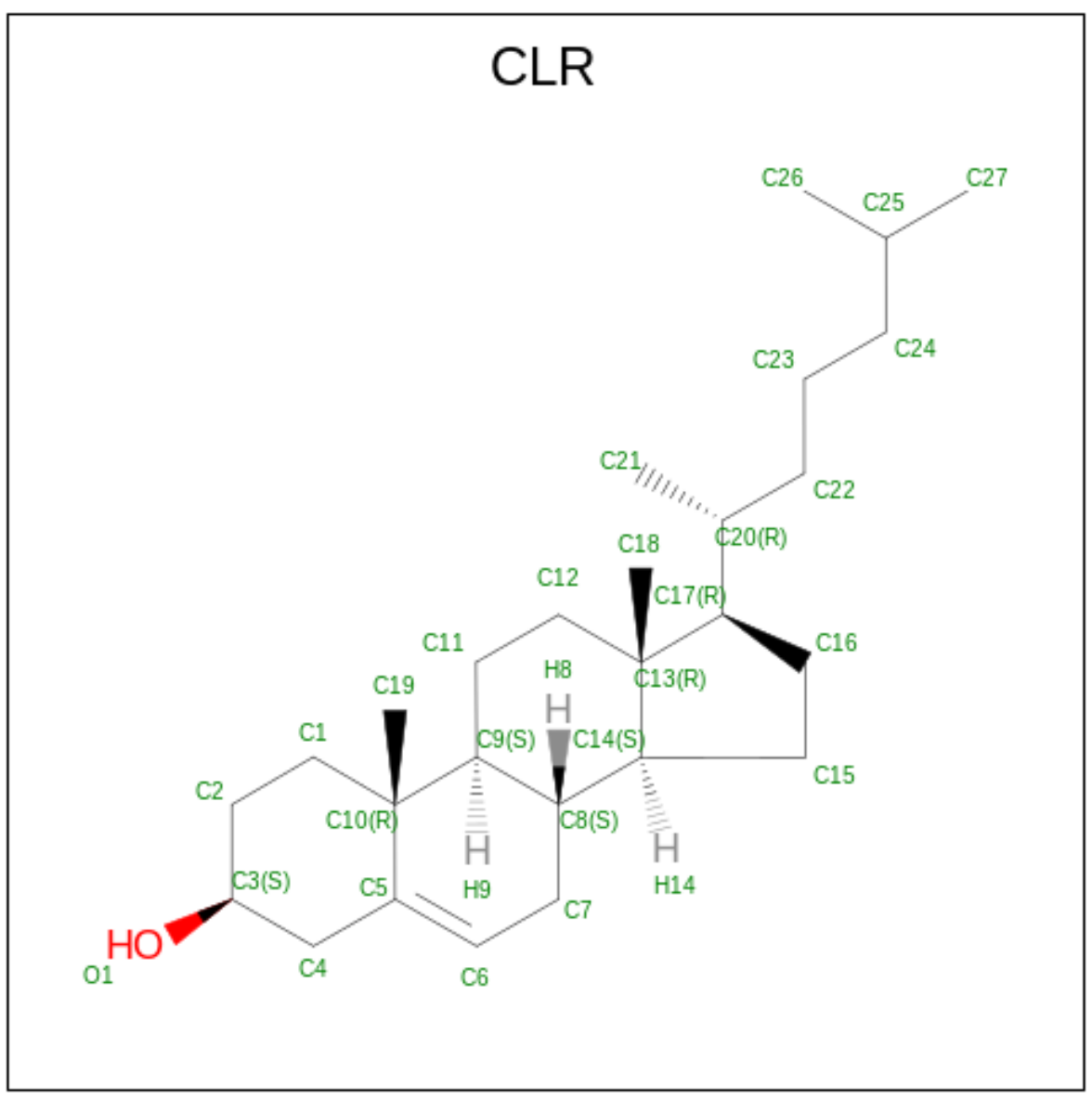

Mol	Chain	Residues	Atoms			AltConf
			Total	C	O	
2	A	1	28	27	1	0
2	A	1	28	27	1	0
2	A	1	28	27	1	0
2	A	1	28	27	1	0
2	A	1	28	27	1	0

Continued on next page...

For Manuscript Review

Continued from previous page...

Mol	Chain	Residues	Atoms	AltConf
2	B	1	Total C O<br>28 27 1	0
2	B	1	Total C O<br>28 27 1	0
2	B	1	Total C O<br>28 27 1	0
2	B	1	Total C O<br>28 27 1	0
2	B	1	Total C O<br>28 27 1	0

- Molecule 3 is HEXADECANE (CCD ID: R16) (formula: $C_{16}H_{34}$).

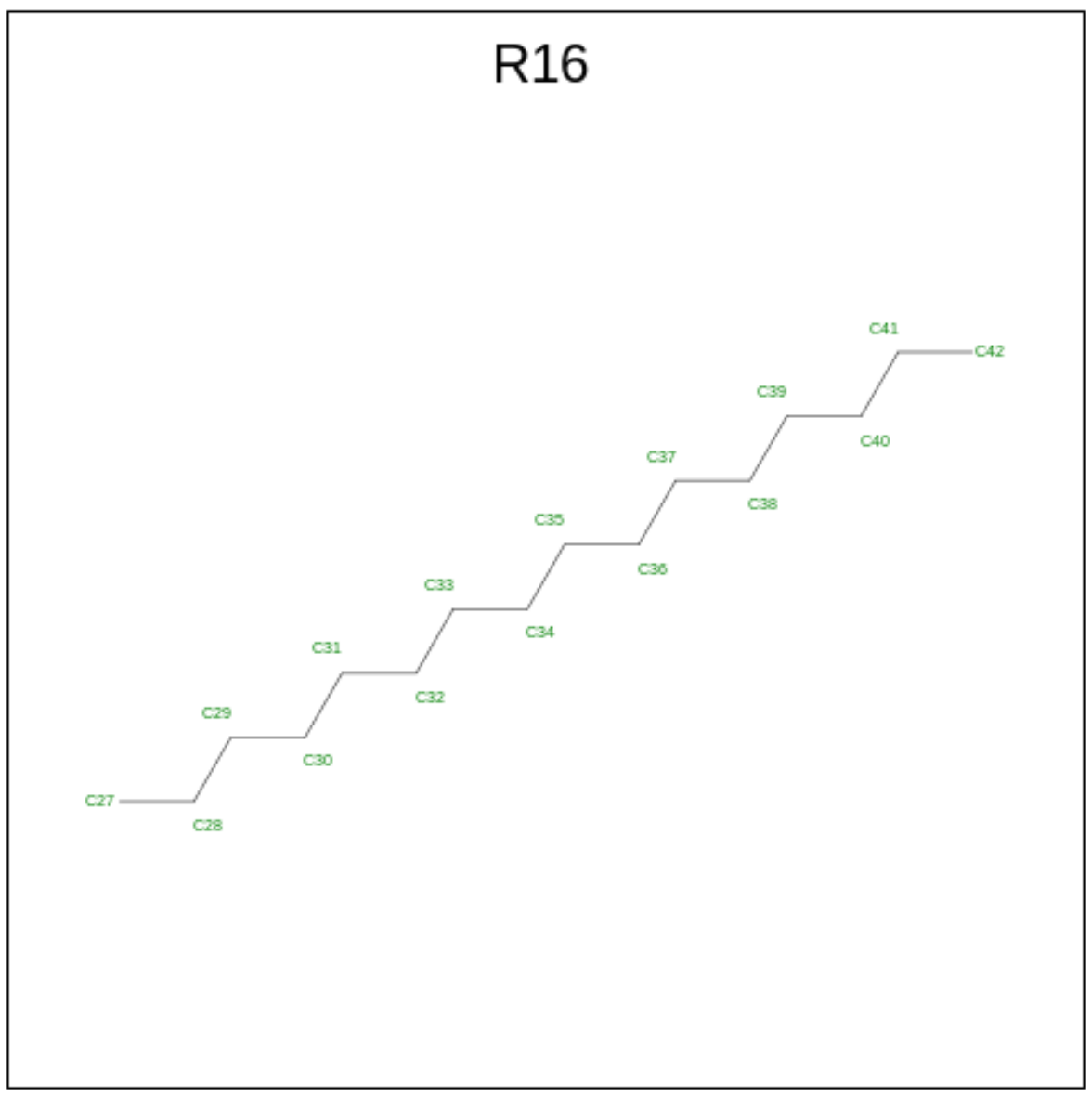

Mol	Chain	Residues	Atoms	AltConf
3	B	1	Total C<br>16 16	0
3	B	1	Total C<br>16 16	0

For Manuscript Review

3 Residue-property plots

These plots are drawn for all protein, RNA, DNA and oligosaccharide chains in the entry. The first graphic for a chain summarises the proportions of the various outlier classes displayed in the second graphic. The second graphic shows the sequence view annotated by issues in geometry and atom inclusion in map density. Residues are color-coded according to the number of geometric quality criteria for which they contain at least one outlier: green = 0, yellow = 1, orange = 2 and red = 3 or more. A red diamond above a residue indicates a poor fit to the EM map for this residue (all-atom inclusion < 40%). Stretches of 2 or more consecutive residues without any outlier are shown as a green connector. Residues present in the sample, but not in the model, are shown in grey.

- Molecule 1: ABC transporter C family member 2

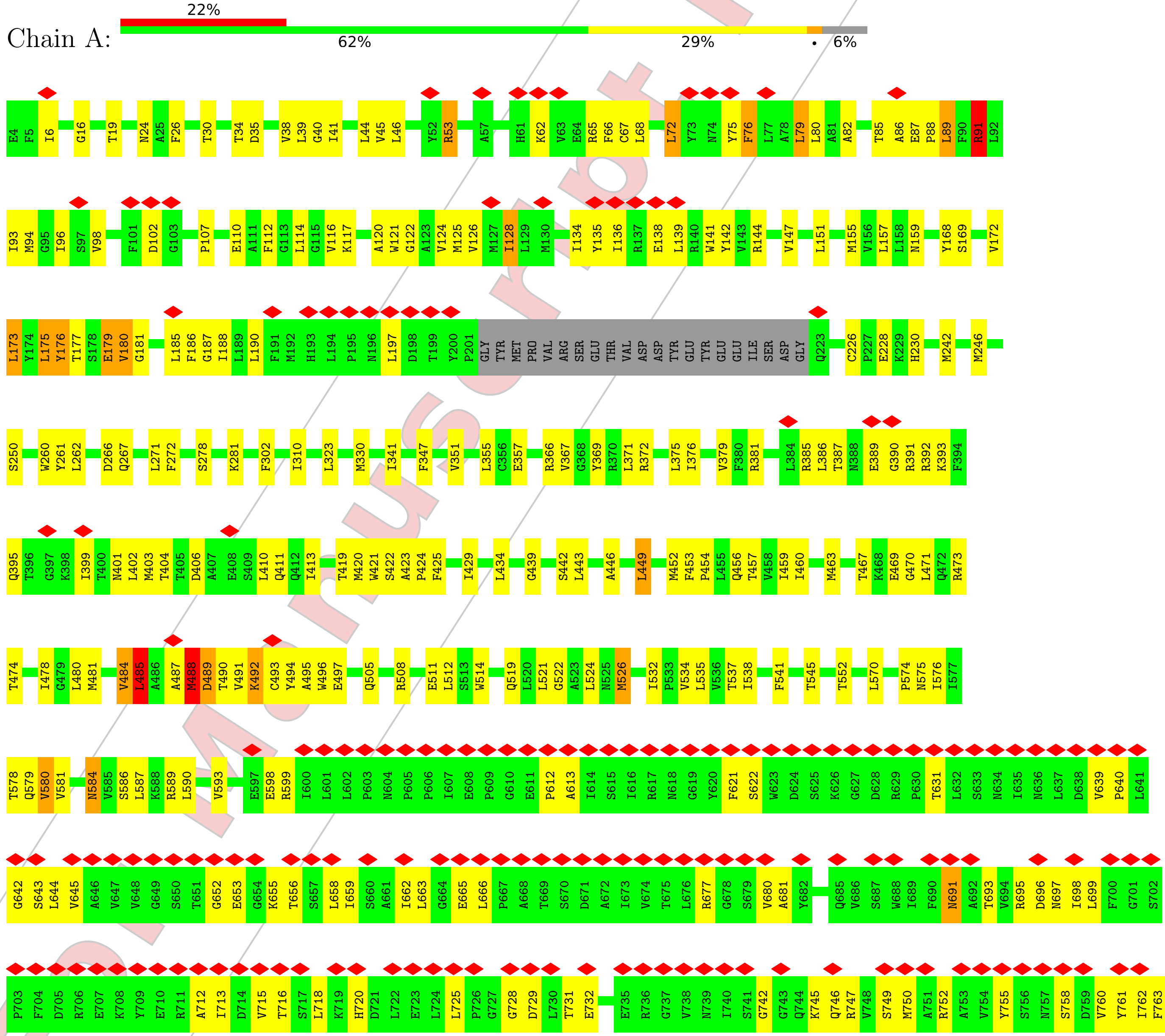

For Manuscript Review

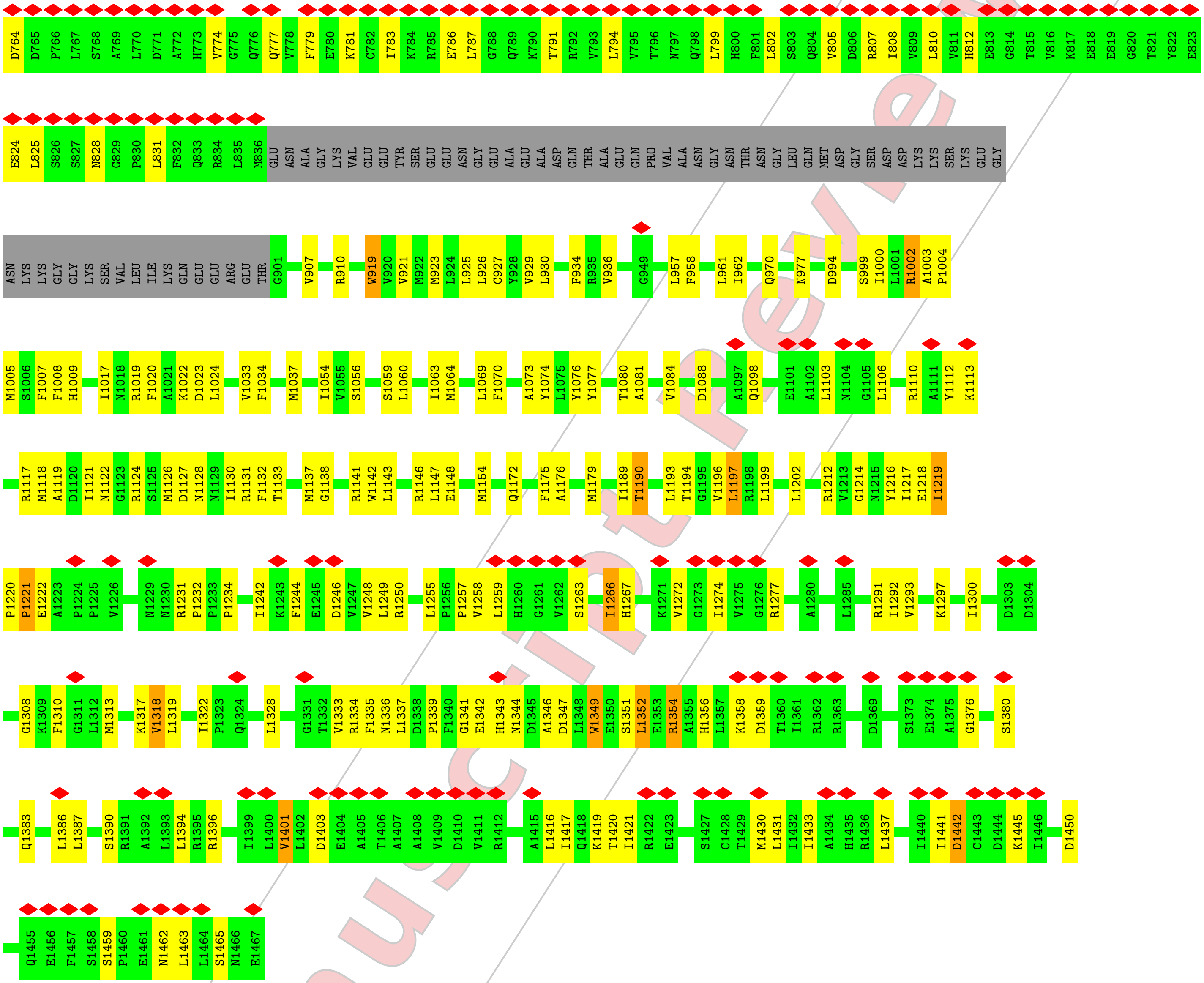

• Molecule 1: ABC transporter C family member 2

Chain B:

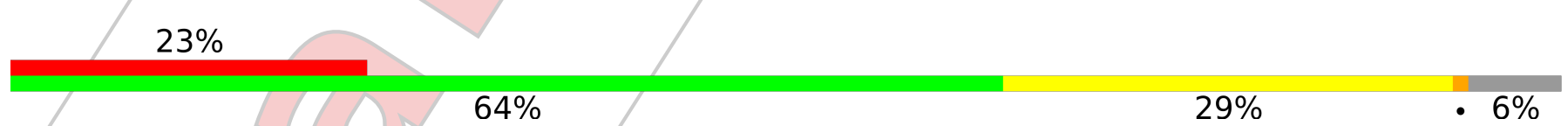

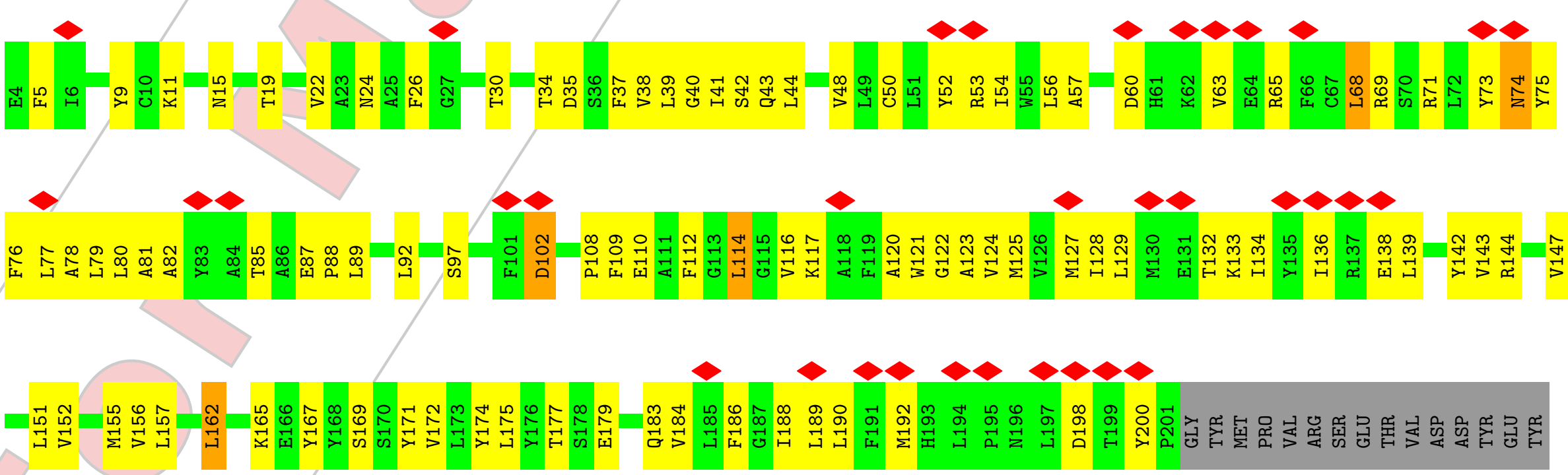

For Manuscript Review

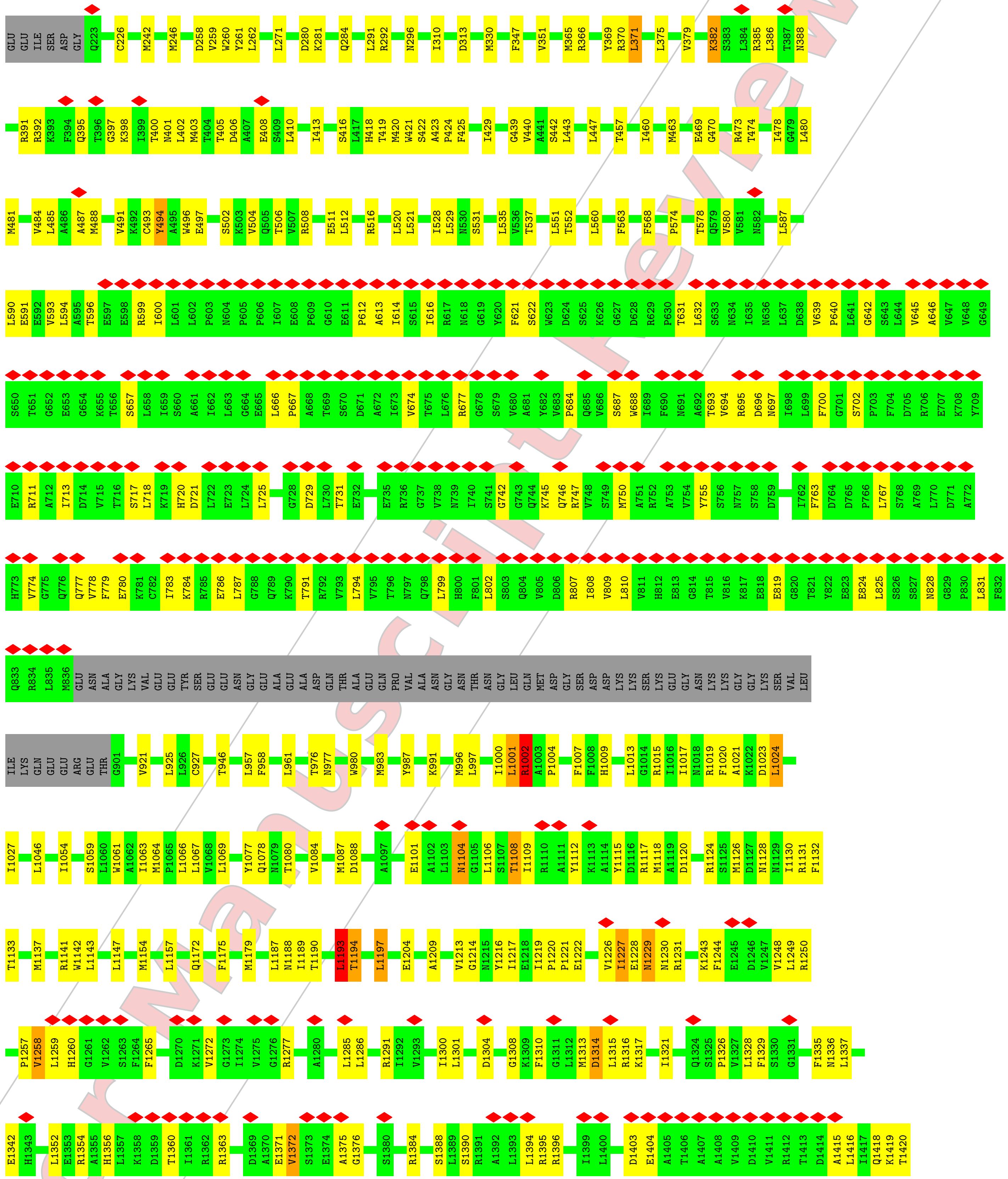

For Manuscript Review

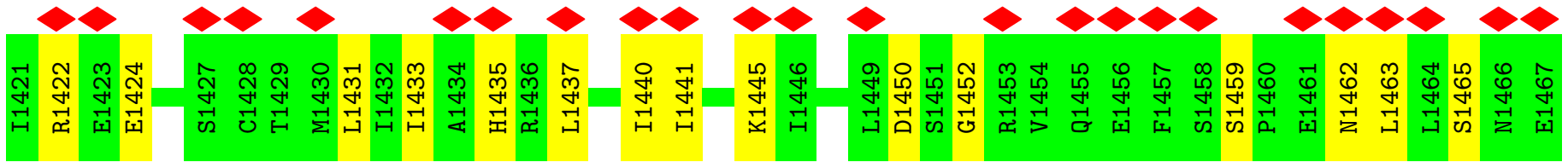

For Manuscript Review

4 Experimental information (i)

Property	Value	Source
EM reconstruction method	SINGLE PARTICLE	Depositor
Imposed symmetry	POINT, Not provided	
Number of particles used	39873	Depositor
Resolution determination method	FSC 0.143 CUT-OFF	Depositor
CTF correction method	PHASE FLIPPING AND AMPLITUDE CORRECTION	Depositor
Microscope	TFS KRIOS	Depositor
Voltage (kV)	300	Depositor
Electron dose ($e^-/\text{Å}^2$)	49.72	Depositor
Minimum defocus (nm)	1200	Depositor
Maximum defocus (nm)	2500	Depositor
Magnification	Not provided	
Image detector	FEI FALCON IV (4k x 4k)	Depositor
Maximum map value	1.104	Depositor
Minimum map value	-1.053	Depositor
Average map value	0.003	Depositor
Map value standard deviation	0.032	Depositor
Recommended contour level	0.15	Depositor
Map size (Å)	300.0, 300.0, 300.0	wwPDB
Map dimensions	400, 400, 400	wwPDB
Map angles (°)	90.0, 90.0, 90.0	wwPDB
Pixel spacing (Å)	0.75, 0.75, 0.75	Depositor

For Manuscript Review

5 Model quality (i)

5.1 Standard geometry (i)

Bond lengths and bond angles in the following residue types are not validated in this section: R16, CLR

The Z score for a bond length (or angle) is the number of standard deviations the observed value is removed from the expected value. A bond length (or angle) with $|Z| > 5$ is considered an outlier worth inspection. RMSZ is the root-mean-square of all Z scores of the bond lengths (or angles).

Mol	Chain	Bond lengths		Bond angles	
		RMSZ	$\#\|Z\| >5$	RMSZ	$\#\|Z\| >5$
1	A	0.35	7/11160 (0.1%)	0.55	10/15159 (0.1%)
1	B	0.27	3/11160 (0.0%)	0.45	2/15159 (0.0%)
All	All	0.31	10/22320 (0.0%)	0.50	12/30318 (0.0%)

Chiral center outliers are detected by calculating the chiral volume of a chiral center and verifying if the center is modelled as a planar moiety or with the opposite hand.A planarity outlier is detected by checking planarity of atoms in a peptide group, atoms in a mainchain group or atoms of a sidechain that are expected to be planar.

Mol	Chain	#Chirality outliers	#Planarity outliers
1	A	0	2
1	B	0	2
All	All	0	4

All (10) bond length outliers are listed below:

Mol	Chain	Res	Type	Atoms	Z	Observed(Å)	Ideal(Å)
1	A	1190	THR	C-O	6.94	1.32	1.24
1	B	1190	THR	C-O	6.59	1.31	1.24
1	B	1193	LEU	C-O	6.45	1.31	1.24
1	A	489	ASP	C-O	6.16	1.31	1.24
1	A	72	LEU	C-O	5.93	1.31	1.23
1	A	1193	LEU	C-O	5.91	1.30	1.24
1	A	76	PHE	C-O	5.55	1.30	1.24
1	A	176	TYR	C-O	5.52	1.30	1.24
1	B	1188	ASN	C-O	5.10	1.30	1.24
1	A	485	LEU	C-O	5.07	1.29	1.24

All (12) bond angle outliers are listed below:

For Manuscript Review

Mol	Chain	Res	Type	Atoms	Z	Observed(°)	Ideal(°)
1	A	1221	PRO	CA-C-O	-9.30	110.18	122.08
1	A	485	LEU	N-CA-C	-9.28	100.73	111.03
1	A	488	MET	N-CA-C	-8.66	102.16	112.89
1	A	489	ASP	N-CA-C	-7.19	103.37	111.14
1	A	180	VAL	CA-C-N	6.39	127.08	119.98
1	A	180	VAL	C-N-CA	6.39	127.08	119.98
1	A	1196	VAL	N-CA-C	-6.03	104.63	110.42
1	B	1001	LEU	N-CA-C	-5.66	104.95	113.89
1	A	492	LYS	N-CA-C	-5.29	104.92	111.33
1	A	484	VAL	CA-C-N	-5.27	113.47	120.63
1	A	484	VAL	C-N-CA	-5.27	113.47	120.63
1	B	1002	ARG	N-CA-C	-5.08	106.22	114.09

There are no chirality outliers.

All (4) planarity outliers are listed below:

Mol	Chain	Res	Type	Group
1	A	1354	ARG	Sidechain
1	A	91	ARG	Sidechain
1	B	1002	ARG	Sidechain
1	B	1187	LEU	Mainchain

5.2 Too-close contacts ⓘ

In the following table, the Non-H and H(model) columns list the number of non-hydrogen atoms and hydrogen atoms in the chain respectively. The H(added) column lists the number of hydrogen atoms added and optimized by MolProbity. The Clashes column lists the number of clashes within the asymmetric unit, whereas Symm-Clashes lists symmetry-related clashes.

Mol	Chain	Non-H	H(model)	H(added)	Clashes	Symm-Clashes
1	A	10915	0	11098	339	0
1	B	10915	0	11098	306	0
2	A	140	0	230	22	0
2	B	140	0	230	30	0
3	B	32	0	68	16	0
All	All	22142	0	22724	660	0

The all-atom clashscore is defined as the number of clashes found per 1000 atoms (including hydrogen atoms). The all-atom clashscore for this structure is 15.

All (660) close contacts within the same asymmetric unit are listed below, sorted by their clash magnitude.

For Manuscript Review

Atom-1	Atom-2	Interatomic distance (Å)	Clash overlap (Å)
1:A:958:PHE:CE1	3:B:1502:R16:H423	1.76	1.18
1:A:958:PHE:CD1	3:B:1502:R16:H423	1.82	1.14
1:A:85:THR:HB	1:A:125:MET:HE2	1.31	1.06
1:A:484:VAL:HA	1:A:487:ALA:HB3	1.31	1.05
1:A:958:PHE:CD1	3:B:1502:R16:C42	2.44	0.99
1:A:958:PHE:CE1	3:B:1502:R16:C42	2.56	0.88
1:B:1147:LEU:HD11	1:B:1197:LEU:HD22	1.55	0.87
1:A:386:LEU:HB3	1:A:392:ARG:HH12	1.38	0.87
1:A:484:VAL:HA	1:A:487:ALA:CB	2.06	0.85
2:A:1504:CLR:H151	2:B:1503:CLR:H122	1.59	0.83
1:B:85:THR:HG22	1:B:121:TRP:HB3	1.61	0.82
1:A:85:THR:CB	1:A:125:MET:HE2	2.09	0.81
1:B:1109:ILE:HD11	1:B:1118:MET:HE2	1.66	0.78
1:A:246:MET:HE2	1:A:1080:THR:HG21	1.66	0.77
1:A:640:PRO:HD2	1:A:807:ARG:HH22	1.47	0.77
2:A:1505:CLR:H213	2:B:1507:CLR:H17	1.66	0.77
1:B:1313:MET:SD	1:B:1313:MET:N	2.58	0.76
1:A:272:PHE:HZ	1:A:381:ARG:HD3	1.51	0.76
1:B:457:THR:HA	1:B:460:ILE:HG12	1.69	0.75
1:A:1118:MET:SD	1:A:1122:ASN:ND2	2.58	0.75
1:A:457:THR:HA	1:A:460:ILE:HG12	1.69	0.75
1:B:92:LEU:HB2	1:B:114:LEU:HD11	1.68	0.74
1:A:1147:LEU:HD11	1:A:1197:LEU:HD13	1.68	0.74
1:B:484:VAL:HG22	1:B:488:MET:HE3	1.70	0.74
1:B:34:THR:O	1:B:38:VAL:HG22	1.88	0.74
1:A:958:PHE:HE1	3:B:1502:R16:H423	1.47	0.73
1:B:1115:TYR:HA	1:B:1118:MET:HE1	1.70	0.73
1:B:1328:LEU:HD12	1:B:1376:GLY:HA3	1.70	0.73
1:A:147:VAL:HG11	1:A:190:LEU:HD21	1.71	0.73
1:B:1314:ASP:OD1	1:B:1314:ASP:N	2.22	0.73
1:A:155:MET:HE2	2:A:1504:CLR:H183	1.71	0.72
1:A:68:LEU:HA	1:A:197:LEU:HD22	1.70	0.72
1:A:484:VAL:CA	1:A:487:ALA:HB3	2.14	0.71
2:A:1504:CLR:H71	2:B:1503:CLR:H14	1.71	0.71
1:A:391:ARG:HG3	1:A:392:ARG:HG2	1.73	0.71
1:A:46:LEU:HD22	1:A:120:ALA:HB1	1.73	0.71
1:A:962:ILE:HD11	3:B:1502:R16:H391	1.73	0.70
1:A:1054:ILE:HG23	2:B:1503:CLR:H212	1.73	0.70
1:B:50:CYS:HB2	1:B:127:MET:HG2	1.73	0.70
1:A:1081:ALA:HA	1:A:1084:VAL:HG22	1.73	0.70
1:A:387:THR:HA	1:A:599:ARG:HD3	1.73	0.70
1:B:52:TYR:O	1:B:56:LEU:HD12	1.92	0.70

Continued on next page...

For Manuscript Review

Continued from previous page...

Atom-1	Atom-2	Interatomic distance (Å)	Clash overlap (Å)
1:B:371:LEU:O	1:B:375:LEU:HD12	1.92	0.70
1:B:261:TYR:HA	1:B:1131:ARG:HE	1.56	0.69
1:A:691:ASN:O	1:A:691:ASN:ND2	2.18	0.69
1:A:581:VAL:O	1:A:584:ASN:ND2	2.26	0.69
1:A:371:LEU:O	1:A:375:LEU:HD12	1.92	0.69
1:A:26:PHE:HE1	2:A:1502:CLR:H111	1.55	0.69
1:A:85:THR:HB	1:A:125:MET:CE	2.18	0.68
1:B:138:GLU:HB3	1:B:139:LEU:HD12	1.74	0.68
1:A:110:GLU:O	1:A:114:LEU:HD12	1.92	0.68
1:A:91:ARG:HA	1:A:96:ILE:HD12	1.74	0.68
1:B:640:PRO:HD2	1:B:807:ARG:HH22	1.58	0.68
1:A:958:PHE:CD1	3:B:1502:R16:H421	2.28	0.68
1:B:927:CYS:HB3	1:B:977:ASN:HD21	1.58	0.68
1:A:330:MET:HE1	1:A:1179:MET:HG2	1.75	0.68
1:A:622:SER:HA	1:A:631:THR:H	1.58	0.68
1:B:40:GLY:O	1:B:44:LEU:HG	1.93	0.68
1:B:587:LEU:O	1:B:591:GLU:HG2	1.93	0.68
1:B:397:GLY:O	1:B:401:ASN:ND2	2.26	0.67
1:B:122:GLY:HA2	1:B:125:MET:HG3	1.75	0.67
1:B:688:TRP:HB2	1:B:1108:THR:HG21	1.76	0.67
1:B:1291:ARG:NH1	1:B:1308:GLY:O	2.28	0.67
1:A:958:PHE:HD1	3:B:1502:R16:C42	2.09	0.66
1:B:73:TYR:HD1	1:B:74:ASN:H	1.42	0.66
1:B:957:LEU:HD23	2:B:1503:CLR:H162	1.78	0.66
1:A:919:TRP:O	1:A:923:MET:HG2	1.95	0.65
1:B:37:PHE:O	1:B:41:ILE:HG12	1.97	0.65
1:A:505:GLN:NE2	1:A:994:ASP:OD1	2.30	0.65
1:B:961:LEU:HD22	2:B:1503:CLR:H232	1.79	0.65
1:B:1128:ASN:HA	1:B:1131:ARG:HD3	1.78	0.65
1:B:281:LYS:NZ	1:B:296:ASN:OD1	2.28	0.65
1:B:44:LEU:O	1:B:48:VAL:HG23	1.97	0.64
1:B:1101:GLU:O	1:B:1104:ASN:ND2	2.31	0.64
1:A:159:ASN:HB2	2:A:1504:CLR:H182	1.79	0.64
1:A:1059:SER:O	1:A:1063:ILE:HG12	1.98	0.64
1:A:1277:ARG:HH22	1:A:1450:ASP:HA	1.63	0.64
1:B:26:PHE:HE1	2:B:1505:CLR:H111	1.63	0.64
1:A:725:LEU:HD12	1:A:731:THR:HG21	1.80	0.64
1:A:927:CYS:HA	1:A:930:LEU:HD12	1.80	0.63
1:A:999:SER:O	1:A:1003:ALA:HB2	1.98	0.63
1:B:1214:GLY:HA2	1:B:1217:ILE:HG12	1.80	0.63
1:B:927:CYS:HB3	1:B:977:ASN:ND2	2.12	0.63

Continued on next page...

For Manuscript Review

Continued from previous page...

Atom-1	Atom-2	Interatomic distance (Å)	Clash overlap (Å)
1:A:484:VAL:HG23	1:A:487:ALA:HB3	1.80	0.63
1:A:488:MET:HA	1:A:491:VAL:HG22	1.79	0.63
1:B:184:VAL:O	1:B:188:ILE:HG12	1.98	0.63
1:B:996:MET:O	1:B:1000:ILE:HG13	1.99	0.63
1:B:1059:SER:O	1:B:1063:ILE:HG12	1.98	0.63
1:A:1274:ILE:HG22	1:A:1433:ILE:HG22	1.81	0.63
1:A:452:MET:HB3	1:A:532:ILE:HD12	1.80	0.63
1:A:390:GLY:HA2	1:A:393:LYS:HG2	1.80	0.63
1:A:392:ARG:HH21	1:A:395:GLN:HG2	1.64	0.62
1:A:488:MET:HA	1:A:491:VAL:HG13	1.81	0.62
1:A:40:GLY:O	1:A:44:LEU:HG	1.99	0.62
1:B:1277:ARG:HH22	1:B:1450:ASP:HA	1.64	0.62
1:A:39:LEU:HD11	1:A:157:LEU:HD22	1.82	0.62
1:B:102:ASP:OD1	1:B:102:ASP:N	2.32	0.62
1:A:330:MET:HE3	1:A:1176:ALA:HA	1.81	0.62
1:A:446:ALA:HA	1:A:449:LEU:HB3	1.82	0.62
1:B:246:MET:HE1	1:B:1142:TRP:CD2	2.34	0.62
1:B:493:CYS:HA	1:B:1316:ARG:HD2	1.82	0.62
1:A:246:MET:HG2	1:A:1076:TYR:CZ	2.35	0.61
1:A:810:LEU:HD21	1:A:831:LEU:HB2	1.82	0.61
1:B:1120:ASP:O	1:B:1124:ARG:HG2	2.01	0.61
1:B:487:ALA:O	1:B:491:VAL:HG22	2.00	0.61
1:B:1404:GLU:OE2	1:B:1435:HIS:NE2	2.34	0.60
1:A:1019:ARG:HD2	1:A:1216:TYR:HE2	1.67	0.60
1:A:574:PRO:O	1:A:578:THR:HG23	2.01	0.60
1:A:1250:ARG:HE	1:A:1257:PRO:HB3	1.65	0.60
1:A:419:THR:HA	1:A:422:SER:HB3	1.83	0.60
1:A:1214:GLY:HA2	1:A:1217:ILE:HD12	1.84	0.60
1:A:958:PHE:HD1	3:B:1502:R16:H423	1.58	0.59
1:A:1056:SER:HG	1:A:1059:SER:HG	1.46	0.59
1:B:420:MET:HE1	1:B:580:VAL:HG21	1.84	0.59
1:B:463:MET:HG2	1:B:521:LEU:HD22	1.85	0.59
1:B:82:ALA:HA	1:B:125:MET:HE3	1.84	0.59
1:B:291:LEU:HD22	1:B:292:ARG:HH11	1.67	0.59
1:A:779:PHE:HD1	1:A:783:ILE:HD13	1.66	0.59
1:A:1019:ARG:HE	1:A:1212:ARG:HD2	1.67	0.59
1:B:73:TYR:OH	1:B:192:MET:O	2.20	0.59
1:B:996:MET:HE2	1:B:1213:VAL:HB	1.83	0.59
1:A:1074:TYR:HB2	1:A:1197:LEU:HD11	1.84	0.59
1:A:121:TRP:HA	1:A:124:VAL:HG22	1.84	0.58
1:B:87:GLU:HB3	1:B:179:GLU:HG2	1.85	0.58

Continued on next page...

For Manuscript Review

Continued from previous page...

Atom-1	Atom-2	Interatomic distance (Å)	Clash overlap (Å)
1:A:612:PRO:O	1:A:677:ARG:NH2	2.36	0.58
1:B:147:VAL:HG11	1:B:190:LEU:HD21	1.83	0.58
1:B:612:PRO:O	1:B:677:ARG:NH1	2.37	0.58
1:A:1403:ASP:OD1	1:A:1433:ILE:HD11	2.03	0.58
1:B:1216:TYR:HA	1:B:1219:ILE:HG12	1.85	0.58
1:A:53:ARG:HE	1:A:139:LEU:HD22	1.68	0.58
1:A:589:ARG:HH21	1:A:590:LEU:HD23	1.69	0.58
1:A:652:GLY:H	1:A:655:LYS:HG2	1.69	0.58
1:B:186:PHE:CE1	1:B:190:LEU:HD23	2.39	0.58
1:A:1216:TYR:HA	1:A:1219:ILE:HG12	1.86	0.58
1:B:642:GLY:H	1:B:791:THR:HG22	1.67	0.58
1:B:469:GLU:OE2	1:B:473:ARG:NH1	2.37	0.58
1:A:261:TYR:HA	1:A:1131:ARG:HH11	1.67	0.58
1:A:1000:ILE:HD12	1:A:1020:PHE:HE1	1.68	0.58
1:A:1313:MET:SD	1:A:1313:MET:N	2.75	0.58
1:A:185:LEU:HD13	1:A:188:ILE:HD11	1.86	0.57
1:B:71:ARG:HH11	1:B:71:ARG:HG2	1.69	0.57
1:A:1242:ILE:HB	1:A:1266:ILE:HD11	1.85	0.57
1:A:487:ALA:O	1:A:491:VAL:HG13	2.04	0.57
2:A:1505:CLR:H161	2:B:1507:CLR:H122	1.85	0.57
1:B:1272:VAL:HG12	1:B:1445:LYS:HB2	1.86	0.57
1:B:666:LEU:HD12	1:B:667:PRO:HD2	1.86	0.57
1:A:1033:VAL:HG12	1:A:1037:MET:HE3	1.85	0.57
1:B:718:LEU:HA	1:B:747:ARG:HD3	1.87	0.57
1:B:1106:LEU:HA	1:B:1109:ILE:HG22	1.85	0.56
1:A:1267:HIS:H	1:A:1445:LYS:HZ1	1.53	0.56
1:A:366:ARG:HA	1:A:369:TYR:CE2	2.40	0.56
1:A:728:GLY:O	1:A:731:THR:OG1	2.23	0.56
1:A:1334:ARG:HA	1:A:1337:LEU:HD12	1.85	0.56
1:B:1258:VAL:HG23	1:B:1259:LEU:HD12	1.87	0.56
1:A:107:PRO:HG2	1:A:110:GLU:HG2	1.87	0.56
1:B:38:VAL:HG12	1:B:1061:TRP:CE2	2.39	0.56
1:B:124:VAL:HA	1:B:127:MET:SD	2.45	0.56
1:B:366:ARG:HA	1:B:369:TYR:CE2	2.40	0.56
1:B:1463:LEU:HD23	1:B:1465:SER:H	1.71	0.56
1:B:400:THR:HA	1:B:403:MET:HE1	1.88	0.56
1:B:60:ASP:O	1:B:65:ARG:NH2	2.39	0.56
1:B:481:MET:HE2	1:B:484:VAL:HG11	1.87	0.56
1:A:1232:PRO:HB3	1:A:1310:PHE:HZ	1.70	0.56
1:B:44:LEU:HD23	1:B:116:VAL:HG21	1.88	0.56
1:B:725:LEU:HG	1:B:731:THR:HG21	1.88	0.56

Continued on next page...

For Manuscript Review

Continued from previous page...

Atom-1	Atom-2	Interatomic distance (Å)	Clash overlap (Å)
1:B:1354:ARG:NH1	1:B:1424:GLU:OE1	2.39	0.56
1:A:1060:LEU:HG	1:A:1064:MET:SD	2.47	0.55
1:B:77:LEU:HG	1:B:189:LEU:HB3	1.88	0.55
1:B:574:PRO:O	1:B:578:THR:HG23	2.05	0.55
1:A:420:MET:HE1	1:A:580:VAL:HG21	1.88	0.55
1:B:188:ILE:HG22	1:B:192:MET:HE1	1.87	0.55
1:A:302:PHE:HD1	1:A:367:VAL:HG11	1.72	0.55
1:B:1004:PRO:HB2	1:B:1007:PHE:HB3	1.88	0.55
1:A:642:GLY:H	1:A:791:THR:HG22	1.72	0.55
1:A:403:MET:HE1	1:A:589:ARG:HH22	1.71	0.55
1:B:921:VAL:O	1:B:925:LEU:HG	2.07	0.55
1:B:1227:ILE:H	1:B:1231:ARG:NH2	2.05	0.55
1:B:1249:LEU:HB3	1:B:1259:LEU:HD13	1.89	0.55
1:A:330:MET:HG2	1:A:1176:ALA:HB2	1.89	0.54
1:A:586:SER:O	1:A:590:LEU:HG	2.07	0.54
1:A:621:PHE:HB3	1:A:666:LEU:HB3	1.89	0.54
1:A:1007:PHE:CZ	1:A:1216:TYR:HE1	2.25	0.54
1:B:1126:MET:O	1:B:1130:ILE:HG12	2.06	0.54
1:B:165:LYS:HD2	1:B:165:LYS:O	2.07	0.54
1:B:1390:SER:O	1:B:1394:LEU:N	2.40	0.54
2:B:1506:CLR:H121	2:B:1506:CLR:H221	1.89	0.54
1:A:480:LEU:O	1:A:484:VAL:HG12	2.07	0.54
1:A:484:VAL:HG22	1:A:488:MET:HG2	1.88	0.54
1:A:535:LEU:HA	1:A:538:ILE:HG12	1.90	0.54
1:B:35:ASP:OD1	1:B:39:LEU:HD12	2.07	0.54
1:B:1133:THR:O	1:B:1137:MET:HG3	2.08	0.54
1:A:402:LEU:C	1:A:403:MET:HE2	2.31	0.54
1:A:266:ASP:OD2	1:A:366:ARG:NH2	2.40	0.54
1:B:516:ARG:HH11	1:B:983:MET:HE1	1.73	0.54
1:B:711:ARG:NH1	1:B:786:GLU:OE1	2.41	0.54
1:A:181:GLY:O	1:A:185:LEU:HD23	2.08	0.54
1:A:278:SER:O	1:A:281:LYS:HG3	2.08	0.54
1:B:313:ASP:OD2	1:B:422:SER:OG	2.26	0.54
1:A:141:TRP:HA	1:A:144:ARG:HG3	1.90	0.53
1:A:495:ALA:HB1	1:A:1317:LYS:HZ2	1.72	0.53
1:B:81:ALA:O	1:B:85:THR:HG23	2.07	0.53
1:B:590:LEU:HA	1:B:593:VAL:HB	1.90	0.53
1:B:810:LEU:HD21	1:B:831:LEU:HB2	1.90	0.53
1:A:808:ILE:HG22	1:A:825:LEU:HD12	1.90	0.53
1:A:495:ALA:HB1	1:A:1317:LYS:NZ	2.23	0.53
1:A:452:MET:O	1:A:456:GLN:N	2.29	0.53

Continued on next page...

For Manuscript Review

Continued from previous page...

Atom-1	Atom-2	Interatomic distance (Å)	Clash overlap (Å)
1:B:259:VAL:HG23	1:B:1131:ARG:HH22	1.74	0.53
1:A:1019:ARG:HD2	1:A:1216:TYR:CE2	2.43	0.53
1:A:1234:PRO:HG3	1:B:1396:ARG:HH22	1.74	0.53
1:A:923:MET:HE2	1:B:976:THR:HA	1.90	0.53
1:B:112:PHE:O	1:B:116:VAL:HG12	2.08	0.53
1:B:1259:LEU:HA	1:B:1452:GLY:HA3	1.89	0.53
1:B:1002:ARG:HD2	1:B:1222:GLU:HB2	1.91	0.53
1:A:957:LEU:HD23	2:A:1505:CLR:H162	1.91	0.52
1:B:721:ASP:O	1:B:725:LEU:N	2.42	0.52
1:B:783:ILE:HG12	1:B:794:LEU:HG	1.91	0.52
1:B:957:LEU:HD23	2:B:1503:CLR:C16	2.39	0.52
1:A:228:GLU:OE2	1:A:366:ARG:NH1	2.43	0.52
1:A:372:ARG:HG3	1:A:411:GLN:HG3	1.92	0.52
1:A:1142:TRP:NE1	1:A:1146:ARG:HE	2.07	0.52
1:A:186:PHE:CE1	1:A:190:LEU:HD23	2.45	0.52
1:A:508:ARG:HA	1:A:511:GLU:HG2	1.92	0.52
1:A:926:LEU:O	1:A:929:VAL:HG22	2.10	0.52
1:A:1349:TRP:CH2	1:A:1358:LYS:HG2	2.45	0.52
1:A:242:MET:HE1	1:A:1138:GLY:HA3	1.91	0.52
1:A:484:VAL:CG2	1:A:487:ALA:HB3	2.39	0.52
1:A:799:LEU:HA	1:A:802:LEU:HG	1.92	0.52
1:B:388:ASN:HB3	1:B:391:ARG:HD3	1.92	0.52
1:A:112:PHE:O	1:A:116:VAL:HG12	2.09	0.52
1:A:177:THR:O	1:A:180:VAL:HG22	2.10	0.52
1:A:439:GLY:O	1:A:442:SER:OG	2.28	0.52
1:B:162:LEU:HD13	1:B:165:LYS:HG2	1.91	0.52
1:A:487:ALA:O	1:A:491:VAL:N	2.43	0.52
1:B:1459:SER:OG	1:B:1462:ASN:ND2	2.43	0.52
1:A:459:ILE:O	1:A:463:MET:HG3	2.10	0.52
1:B:693:THR:HG23	1:B:695:ARG:H	1.74	0.52
1:B:958:PHE:CD1	3:B:1501:R16:C42	2.93	0.52
1:B:1088:ASP:OD1	1:B:1132:PHE:HB3	2.10	0.52
1:A:403:MET:HE1	1:A:589:ARG:NH2	2.25	0.52
1:B:961:LEU:HD22	2:B:1503:CLR:C23	2.39	0.52
1:A:262:LEU:CD1	1:A:1127:ASP:HB3	2.40	0.51
1:A:488:MET:HA	1:A:491:VAL:CG2	2.39	0.51
1:A:1054:ILE:HG12	2:B:1503:CLR:C21	2.40	0.51
1:A:82:ALA:HA	1:A:125:MET:HE1	1.93	0.51
1:B:69:ARG:NH1	1:B:198:ASP:OD2	2.44	0.51
1:B:123:ALA:O	1:B:127:MET:HG3	2.09	0.51
1:B:481:MET:HE2	1:B:481:MET:HA	1.93	0.51

Continued on next page...

For Manuscript Review

Continued from previous page...

Atom-1	Atom-2	Interatomic distance (Å)	Clash overlap (Å)
1:B:382:LYS:HA	1:B:385:ARG:HG2	1.92	0.51
1:A:488:MET:CA	1:A:491:VAL:HG22	2.41	0.51
1:A:695:ARG:O	1:A:699:LEU:HG	2.11	0.51
1:A:961:LEU:HD22	2:A:1505:CLR:H231	1.92	0.51
1:B:777:GLN:NE2	1:B:778:VAL:HG23	2.26	0.51
1:A:644:LEU:HD23	1:A:805:VAL:HA	1.90	0.51
1:A:697:ASN:O	1:A:752:ARG:NE	2.43	0.51
1:B:117:LYS:O	1:B:121:TRP:HD1	1.94	0.51
1:B:487:ALA:HB3	1:B:488:MET:HE2	1.93	0.51
1:B:808:ILE:HG22	1:B:825:LEU:HD12	1.93	0.51
1:A:1106:LEU:O	1:A:1110:ARG:HB3	2.11	0.51
1:A:587:LEU:HD12	1:A:590:LEU:HD12	1.93	0.51
1:B:401:ASN:O	1:B:405:THR:OG1	2.22	0.51
1:A:389:GLU:HB2	1:A:391:ARG:HG2	1.94	0.50
1:A:488:MET:HA	1:A:491:VAL:CG1	2.40	0.50
1:B:824:GLU:O	1:B:828:ASN:HB2	2.11	0.50
1:A:691:ASN:HD22	1:A:691:ASN:C	2.13	0.50
1:A:1249:LEU:H	1:A:1259:LEU:HB2	1.76	0.50
1:B:26:PHE:CE1	2:B:1505:CLR:H111	2.44	0.50
1:B:78:ALA:HA	1:B:128:ILE:HD12	1.92	0.50
1:B:385:ARG:O	1:B:391:ARG:NH2	2.44	0.50
1:B:958:PHE:CE1	3:B:1501:R16:H412	2.46	0.50
1:A:85:THR:OG1	1:A:121:TRP:HB3	2.11	0.50
1:A:1002:ARG:HH21	1:A:1222:GLU:C	2.19	0.50
1:B:1329:PHE:HB2	1:B:1335:PHE:HE2	1.76	0.50
1:A:925:LEU:O	1:A:929:VAL:HG13	2.11	0.50
1:A:1349:TRP:CZ3	1:A:1358:LYS:HG2	2.46	0.50
1:B:1360:THR:HA	1:B:1363:ARG:HD3	1.92	0.50
1:A:136:ILE:HG22	1:A:138:GLU:H	1.75	0.50
1:A:1274:ILE:CG2	1:A:1433:ILE:HG22	2.42	0.50
1:B:508:ARG:HA	1:B:511:GLU:HG2	1.92	0.50
1:A:1128:ASN:O	1:A:1131:ARG:HG2	2.12	0.50
1:B:139:LEU:HA	1:B:142:TYR:HD2	1.77	0.50
1:A:469:GLU:O	1:A:473:ARG:HG2	2.12	0.50
1:A:681:ALA:HB2	1:A:758:SER:HB2	1.93	0.50
1:B:330:MET:HE1	1:B:1179:MET:HG2	1.94	0.50
1:B:365:MET:HE1	1:B:1141:ARG:HH21	1.77	0.50
1:B:599:ARG:HD3	1:B:600:ILE:H	1.77	0.50
1:B:799:LEU:HA	1:B:802:LEU:HG	1.94	0.50
1:B:1243:LYS:HB3	1:B:1265:PHE:HD1	1.77	0.50
1:B:259:VAL:HG23	1:B:1131:ARG:NH2	2.26	0.50

Continued on next page...

For Manuscript Review

Continued from previous page...

Atom-1	Atom-2	Interatomic distance (Å)	Clash overlap (Å)
1:B:957:LEU:HG	2:B:1503:CLR:H222	1.94	0.50
1:A:457:THR:O	1:A:459:ILE:N	2.44	0.49
1:A:824:GLU:O	1:A:828:ASN:HB2	2.12	0.49
1:B:645:VAL:HG22	1:B:807:ARG:HE	1.77	0.49
1:A:680:VAL:HA	1:A:760:VAL:HB	1.94	0.49
1:A:910:ARG:NH2	1:A:1218:GLU:OE2	2.46	0.49
1:A:742:GLY:HA2	1:A:745:LYS:HE2	1.94	0.49
1:A:659:ILE:O	1:A:663:LEU:HD22	2.12	0.49
1:A:693:THR:O	1:A:697:ASN:ND2	2.45	0.49
1:B:1063:ILE:HD12	1:B:1154:MET:HG3	1.93	0.49
1:A:491:VAL:CG2	1:A:497:GLU:HG3	2.43	0.49
1:B:696:ASP:O	1:B:1117:ARG:NH1	2.45	0.49
1:A:386:LEU:HD22	1:A:392:ARG:HH22	1.77	0.49
1:A:575:ASN:O	1:A:579:GLN:HG2	2.12	0.49
1:A:729:ASP:OD1	1:A:729:ASP:N	2.42	0.49
2:A:1505:CLR:H211	1:B:1054:ILE:HG12	1.94	0.49
1:A:34:THR:O	1:A:38:VAL:HG22	2.12	0.49
1:B:155:MET:HG2	2:B:1507:CLR:H222	1.95	0.49
1:B:485:LEU:HD12	1:B:1013:LEU:HG	1.94	0.49
1:B:696:ASP:OD1	1:B:1117:ARG:NH2	2.46	0.49
1:A:653:GLU:HB3	1:A:812:HIS:HA	1.95	0.49
1:A:1401:VAL:HG12	1:A:1431:LEU:HB3	1.93	0.49
1:A:310:ILE:HG13	1:A:421:TRP:HH2	1.78	0.49
1:B:391:ARG:HG3	1:B:395:GLN:HG2	1.95	0.49
1:B:713:ILE:HG23	1:B:718:LEU:HB3	1.94	0.49
1:B:1064:MET:HA	1:B:1067:LEU:HD12	1.96	0.48
1:B:1250:ARG:HE	1:B:1257:PRO:HB3	1.77	0.48
1:B:1416:LEU:HD12	1:B:1419:LYS:HD2	1.95	0.48
1:A:272:PHE:CZ	1:A:381:ARG:HD3	2.40	0.48
1:A:1098:GLN:HB3	1:A:1122:ASN:HD21	1.79	0.48
1:A:1258:VAL:HG12	1:A:1259:LEU:HD23	1.94	0.48
1:B:259:VAL:O	1:B:1131:ARG:NH2	2.46	0.48
1:B:695:ARG:NH2	1:B:729:ASP:O	2.47	0.48
1:B:1209:ALA:O	1:B:1213:VAL:HG22	2.13	0.48
1:A:86:ALA:O	1:A:89:LEU:HB2	2.13	0.48
1:A:155:MET:CE	2:A:1504:CLR:H183	2.41	0.48
1:A:260:TRP:O	1:A:1131:ARG:HD2	2.13	0.48
1:A:1005:MET:HG2	1:A:1292:ILE:HG23	1.96	0.48
1:A:485:LEU:HG	1:A:1017:ILE:HD11	1.95	0.48
1:A:1143:LEU:HD21	1:A:1197:LEU:HD22	1.95	0.48
1:B:382:LYS:HD2	1:B:594:LEU:HA	1.95	0.48

Continued on next page...

For Manuscript Review

Continued from previous page...

Atom-1	Atom-2	Interatomic distance (Å)	Clash overlap (Å)
1:B:684:PRO:HG2	1:B:687:SER:HA	1.95	0.48
1:B:717:SER:OG	1:B:777:GLN:NE2	2.36	0.48
1:B:957:LEU:HB3	2:B:1503:CLR:C15	2.43	0.48
1:A:934:PHE:HB3	1:A:970:GLN:HB2	1.95	0.48
1:B:496:TRP:HE1	1:B:1395:ARG:HH21	1.61	0.48
1:A:87:GLU:HG3	1:A:179:GLU:HG2	1.94	0.48
1:A:463:MET:HG2	1:A:521:LEU:HD22	1.95	0.48
1:A:715:VAL:HA	1:A:781:LYS:HB3	1.94	0.48
1:A:1003:ALA:HB1	1:A:1004:PRO:HD2	1.96	0.48
1:B:1301:LEU:HG	1:B:1304:ASP:HA	1.96	0.48
1:A:957:LEU:HB3	2:A:1505:CLR:H152	1.96	0.48
1:A:1291:ARG:NH1	1:A:1308:GLY:O	2.46	0.48
1:B:39:LEU:HD11	1:B:157:LEU:HD12	1.96	0.48
1:B:958:PHE:HD1	3:B:1501:R16:C42	2.27	0.48
1:A:168:TYR:HB3	1:A:173:LEU:HG	1.96	0.48
1:A:658:LEU:O	1:A:662:ILE:HG13	2.13	0.48
1:A:102:ASP:OD1	1:A:102:ASP:N	2.38	0.47
1:B:1118:MET:SD	1:B:1118:MET:N	2.87	0.47
1:A:121:TRP:O	1:A:124:VAL:HG22	2.13	0.47
1:A:658:LEU:HD21	1:A:764:ASP:HB2	1.96	0.47
1:A:712:ALA:O	1:A:716:THR:OG1	2.30	0.47
1:B:24:ASN:HB3	1:B:30:THR:HG21	1.96	0.47
1:B:53:ARG:HD3	1:B:143:VAL:HG11	1.95	0.47
1:B:502:SER:O	1:B:506:THR:HG23	2.15	0.47
1:B:1066:LEU:HD11	1:B:1157:LEU:HD12	1.96	0.47
1:B:481:MET:O	1:B:484:VAL:HG12	2.14	0.47
1:A:392:ARG:NH2	1:A:395:GLN:OE1	2.47	0.47
1:A:470:GLY:O	1:A:474:THR:HG23	2.14	0.47
1:A:696:ASP:OD1	1:A:1117:ARG:NH2	2.44	0.47
1:B:440:VAL:HA	1:B:443:LEU:HD12	1.97	0.47
1:B:958:PHE:HD1	3:B:1501:R16:H422	1.79	0.47
1:A:76:PHE:HD1	1:A:79:LEU:HD23	1.80	0.47
1:A:644:LEU:HD11	1:A:794:LEU:HB2	1.96	0.47
1:B:613:ALA:N	1:B:639:VAL:O	2.47	0.47
1:A:488:MET:SD	1:A:491:VAL:HG21	2.54	0.47
1:B:551:LEU:HD23	1:B:946:THR:HG22	1.97	0.47
1:A:93:ILE:HG13	1:A:94:MET:HE2	1.97	0.47
1:A:262:LEU:HD11	1:A:1127:ASP:HB3	1.97	0.47
2:A:1505:CLR:H17	2:B:1507:CLR:H14	1.96	0.47
1:B:175:LEU:O	1:B:179:GLU:HG3	2.15	0.47
1:B:1403:ASP:HA	1:B:1433:ILE:HG13	1.96	0.47

Continued on next page...

For Manuscript Review

Continued from previous page...

Atom-1	Atom-2	Interatomic distance (Å)	Clash overlap (Å)
1:A:117:LYS:O	1:A:121:TRP:HD1	1.97	0.47
1:A:613:ALA:N	1:A:639:VAL:O	2.48	0.47
1:B:419:THR:HA	1:B:422:SER:HB3	1.97	0.47
2:B:1504:CLR:H221	2:B:1504:CLR:H162	1.51	0.47
1:A:488:MET:O	1:A:491:VAL:HG22	2.15	0.46
1:A:491:VAL:HB	1:A:496:TRP:O	2.15	0.46
1:A:621:PHE:CB	1:A:666:LEU:HD23	2.44	0.46
1:A:713:ILE:HG23	1:A:718:LEU:HB3	1.97	0.46
1:A:761:TYR:C	1:A:762:ILE:HD12	2.41	0.46
1:A:87:GLU:HB3	1:A:88:PRO:HD3	1.96	0.46
1:A:139:LEU:HA	1:A:142:TYR:CD1	2.51	0.46
1:A:341:ILE:HD11	2:A:1501:CLR:H161	1.98	0.46
1:A:1077:TYR:CD2	1:A:1143:LEU:HD22	2.50	0.46
1:A:1341:GLY:C	1:A:1343:HIS:H	2.23	0.46
1:A:1390:SER:O	1:A:1394:LEU:N	2.48	0.46
1:B:129:LEU:O	1:B:132:THR:OG1	2.21	0.46
1:B:1375:ALA:O	1:B:1384:ARG:NH2	2.28	0.46
1:A:1019:ARG:HA	1:A:1023:ASP:OD2	2.14	0.46
1:B:366:ARG:HD3	1:B:370:ARG:HH12	1.80	0.46
1:B:402:LEU:O	1:B:406:ASP:N	2.45	0.46
1:B:742:GLY:HA2	1:B:745:LYS:HE2	1.96	0.46
1:A:1394:LEU:O	1:A:1396:ARG:NH1	2.48	0.46
1:A:1459:SER:OG	1:A:1462:ASN:ND2	2.48	0.46
1:B:379:VAL:HA	1:B:382:LYS:HG2	1.98	0.46
1:B:439:GLY:O	1:B:442:SER:OG	2.32	0.46
1:B:616:ILE:HG12	1:B:674:VAL:HG22	1.96	0.46
1:B:747:ARG:HA	1:B:750:MET:HG2	1.96	0.46
1:A:24:ASN:HB3	1:A:30:THR:HG21	1.97	0.46
1:B:97:SER:HA	1:B:110:GLU:OE2	2.16	0.46
1:B:1078:GLN:NE2	1:B:1204:GLU:OE1	2.48	0.46
1:B:1415:ALA:O	1:B:1419:LYS:NZ	2.49	0.46
1:A:62:LYS:HA	1:A:62:LYS:HD3	1.74	0.46
1:B:392:ARG:HD3	1:B:392:ARG:C	2.41	0.46
2:A:1501:CLR:H162	2:A:1501:CLR:H221	1.46	0.46
2:A:1502:CLR:H221	2:A:1502:CLR:H162	1.39	0.46
1:B:54:ILE:HD12	1:B:57:ALA:HB3	1.97	0.46
1:B:481:MET:HE3	1:B:504:VAL:HG21	1.98	0.46
1:B:639:VAL:HA	1:B:807:ARG:HH22	1.81	0.46
1:A:1322:ILE:O	1:A:1403:ASP:HB2	2.15	0.46
1:B:702:SER:HB2	1:B:755:TYR:CZ	2.51	0.45
1:B:1243:LYS:HB2	1:B:1243:LYS:HE3	1.78	0.45

Continued on next page...

For Manuscript Review

Continued from previous page...

Atom-1	Atom-2	Interatomic distance (Å)	Clash overlap (Å)
1:B:1286:LEU:HD21	1:B:1321:ILE:HD11	1.96	0.45
1:A:66:PHE:HB2	1:A:135:TYR:HD2	1.81	0.45
1:A:522:GLY:O	1:A:526:MET:HG2	2.16	0.45
1:A:621:PHE:HB3	1:A:666:LEU:HD23	1.97	0.45
1:A:1172:GLN:HA	1:A:1175:PHE:HD2	1.80	0.45
1:B:261:TYR:HA	1:B:1131:ARG:NE	2.27	0.45
1:B:1020:PHE:O	1:B:1024:LEU:HB2	2.16	0.45
1:A:122:GLY:O	1:A:126:VAL:HG13	2.16	0.45
1:A:1246:ASP:O	1:A:1297:LYS:NZ	2.38	0.45
1:B:226:CYS:HB3	1:B:260:TRP:CD1	2.51	0.45
1:B:480:LEU:O	1:B:484:VAL:HB	2.15	0.45
1:B:531:SER:O	1:B:535:LEU:HD12	2.17	0.45
1:B:767:LEU:HB3	1:B:779:PHE:CZ	2.51	0.45
1:B:1227:ILE:HG23	1:B:1229:ASN:H	1.80	0.45
1:A:250:SER:HB3	1:A:1076:TYR:HE1	1.81	0.45
1:A:693:THR:HG22	1:A:732:GLU:HG3	1.99	0.45
1:A:1141:ARG:HA	1:A:1141:ARG:HD3	1.81	0.45
1:B:416:SER:O	1:B:416:SER:OG	2.32	0.45
1:B:612:PRO:HA	1:B:640:PRO:HA	1.99	0.45
1:B:1313:MET:HB3	1:B:1317:LYS:HZ2	1.80	0.45
1:A:1352:LEU:HD12	1:A:1352:LEU:HA	1.73	0.45
1:A:1417:ILE:O	1:A:1420:THR:OG1	2.32	0.45
1:B:470:GLY:O	1:B:474:THR:HG23	2.16	0.45
1:B:742:GLY:O	1:B:746:GLN:HG2	2.17	0.45
2:B:1504:CLR:H213	2:B:1504:CLR:H231	1.82	0.45
1:A:53:ARG:HA	1:A:53:ARG:HD3	1.49	0.45
1:A:491:VAL:HG23	1:A:497:GLU:HG3	1.99	0.45
1:B:144:ARG:O	1:B:147:VAL:HG12	2.17	0.45
1:B:958:PHE:HE1	3:B:1501:R16:H412	1.81	0.45
1:A:175:LEU:O	1:A:176:TYR:C	2.59	0.45
1:A:1272:VAL:HG23	1:A:1445:LYS:HG3	1.99	0.45
1:B:1193:LEU:O	1:B:1194:THR:C	2.60	0.45
1:A:1335:PHE:CE1	1:A:1339:PRO:HB3	2.52	0.45
1:B:987:TYR:CZ	1:B:991:LYS:HD3	2.52	0.45
2:B:1507:CLR:H213	2:B:1507:CLR:H232	1.82	0.45
1:A:425:PHE:O	1:A:429:ILE:HG12	2.17	0.45
1:B:71:ARG:HA	1:B:71:ARG:NE	2.32	0.45
1:B:976:THR:HG22	1:B:980:TRP:CD1	2.52	0.45
1:B:1141:ARG:HD3	1:B:1141:ARG:HA	1.78	0.45
1:A:399:ILE:O	1:A:403:MET:HG2	2.17	0.44
1:A:484:VAL:C	1:A:487:ALA:H	2.25	0.44

Continued on next page...

For Manuscript Review

Continued from previous page...

Atom-1	Atom-2	Interatomic distance (Å)	Clash overlap (Å)
1:B:121:TRP:HZ3	1:B:186:PHE:HD2	1.65	0.44
1:B:179:GLU:O	1:B:183:GLN:HG3	2.17	0.44
1:B:786:GLU:HG2	1:B:787:LEU:HG	1.98	0.44
1:A:387:THR:H	1:A:392:ARG:NH1	2.14	0.44
1:A:698:ILE:HA	1:A:752:ARG:HB2	1.99	0.44
1:B:38:VAL:HG12	1:B:1061:TRP:CZ2	2.52	0.44
1:B:478:ILE:HD12	1:B:478:ILE:HA	1.86	0.44
1:A:230:HIS:O	1:A:230:HIS:ND1	2.39	0.44
1:A:1088:ASP:OD1	1:A:1132:PHE:HB3	2.17	0.44
1:A:1416:LEU:HD12	1:A:1419:LYS:HD2	1.99	0.44
1:B:85:THR:O	1:B:88:PRO:HD2	2.17	0.44
1:B:366:ARG:NH1	1:B:369:TYR:OH	2.50	0.44
1:B:1243:LYS:HB3	1:B:1265:PHE:CD1	2.52	0.44
1:A:372:ARG:HD2	1:A:1126:MET:HE1	1.99	0.44
1:A:1148:GLU:OE1	1:A:1190:THR:HB	2.17	0.44
1:A:1216:TYR:O	1:A:1219:ILE:HB	2.17	0.44
1:B:71:ARG:HG2	1:B:71:ARG:NH1	2.32	0.44
1:A:26:PHE:HB2	1:A:30:THR:HG23	1.99	0.44
1:A:267:GLN:HG2	1:A:1124:ARG:HH12	1.83	0.44
1:A:491:VAL:O	1:A:492:LYS:C	2.60	0.44
1:A:718:LEU:HA	1:A:747:ARG:HD3	1.98	0.44
1:A:1069:LEU:HD22	2:A:1503:CLR:H191	2.00	0.44
1:A:1133:THR:O	1:A:1137:MET:HG3	2.17	0.44
1:A:1344:ASN:C	1:A:1346:ALA:N	2.76	0.44
1:B:52:TYR:CE2	1:B:56:LEU:HD11	2.53	0.44
1:B:133:LYS:HE3	1:B:134:ILE:H	1.83	0.44
1:B:1013:LEU:O	1:B:1017:ILE:HG22	2.17	0.44
1:B:408:GLU:OE1	1:B:408:GLU:N	2.43	0.44
1:B:1326:PRO:HB2	1:B:1384:ARG:HG2	2.00	0.44
1:A:41:ILE:O	1:A:45:VAL:HG23	2.18	0.44
1:A:750:MET:HB3	1:A:763:PHE:HE2	1.82	0.44
1:A:1220:PRO:HA	1:A:1221:PRO:HD2	1.92	0.44
1:B:174:TYR:O	1:B:177:THR:N	2.51	0.44
1:B:1248:VAL:HG22	1:B:1260:HIS:HA	2.00	0.44
1:A:155:MET:HE3	1:A:155:MET:O	2.17	0.44
1:A:187:GLY:O	1:A:190:LEU:HG	2.18	0.44
1:A:936:VAL:HG11	3:B:1501:R16:H352	2.00	0.44
1:A:1246:ASP:H	1:A:1263:SER:HG	1.63	0.44
1:B:621:PHE:HB3	1:B:666:LEU:HG	2.00	0.43
1:A:379:VAL:HG11	1:A:403:MET:HG3	2.00	0.43
1:A:656:THR:O	1:A:659:ILE:HG12	2.18	0.43

Continued on next page...

For Manuscript Review

Continued from previous page...

Atom-1	Atom-2	Interatomic distance (Å)	Clash overlap (Å)
1:A:716:THR:HB	1:A:750:MET:HE3	2.00	0.43
1:A:1442:ASP:OD1	1:A:1442:ASP:N	2.35	0.43
1:B:134:ILE:HD11	1:B:200:TYR:CZ	2.54	0.43
1:B:347:PHE:O	1:B:351:VAL:HG22	2.18	0.43
1:B:1172:GLN:HA	1:B:1175:PHE:HD2	1.82	0.43
1:A:643:SER:OG	1:A:807:ARG:NH2	2.52	0.43
1:A:742:GLY:O	1:A:746:GLN:HG2	2.17	0.43
1:A:1317:LYS:HA	1:A:1317:LYS:HD3	1.72	0.43
1:B:694:VAL:HA	1:B:697:ASN:ND2	2.33	0.43
1:B:1321:ILE:HD12	1:B:1321:ILE:HA	1.91	0.43
1:A:121:TRP:O	1:A:125:MET:HG2	2.19	0.43
1:A:179:GLU:H	1:A:179:GLU:HG3	1.54	0.43
1:A:434:LEU:HD23	1:A:434:LEU:HA	1.86	0.43
1:B:136:ILE:HG22	1:B:138:GLU:H	1.83	0.43
1:B:260:TRP:O	1:B:1131:ARG:NE	2.52	0.43
1:A:1328:LEU:HD22	1:A:1376:GLY:N	2.33	0.43
1:A:1354:ARG:C	1:A:1356:HIS:H	2.26	0.43
1:A:185:LEU:O	1:A:188:ILE:HG12	2.19	0.43
1:A:347:PHE:O	1:A:351:VAL:HG22	2.18	0.43
1:A:385:ARG:NH2	1:A:598:GLU:OE2	2.52	0.43
1:A:463:MET:HE2	1:A:463:MET:HB3	1.81	0.43
1:A:1336:ASN:HD21	1:A:1387:LEU:HD21	1.82	0.43
1:A:35:ASP:OD1	1:A:39:LEU:HD12	2.19	0.43
1:A:1054:ILE:HG12	2:B:1503:CLR:H211	2.00	0.43
1:A:1244:PHE:CE1	1:A:1300:ILE:HB	2.54	0.43
1:B:783:ILE:HB	1:B:784:LYS:HE2	2.00	0.43
1:B:1220:PRO:HA	1:B:1221:PRO:HD3	1.94	0.43
1:B:310:ILE:HG13	1:B:421:TRP:HH2	1.84	0.43
1:B:410:LEU:HA	1:B:413:ILE:HG22	2.01	0.43
1:B:997:LEU:HD12	1:B:1001:LEU:HD11	1.99	0.43
1:B:1000:ILE:HD11	1:B:1216:TYR:HB3	2.00	0.43
2:B:1505:CLR:H221	2:B:1505:CLR:H162	1.26	0.43
1:A:67:CYS:O	1:A:197:LEU:HB3	2.18	0.43
1:A:302:PHE:CD1	1:A:367:VAL:HG11	2.52	0.43
1:A:1034:PHE:HE2	1:A:1202:LEU:HD13	1.84	0.43
1:A:1383:GLN:HA	1:A:1386:LEU:HG	2.00	0.43
1:B:1244:PHE:CE1	1:B:1300:ILE:HB	2.54	0.43
1:B:1356:HIS:HE1	1:B:1420:THR:HG22	1.82	0.43
1:A:453:PHE:HB3	1:A:454:PRO:HD3	2.01	0.43
1:B:30:THR:O	1:B:34:THR:HG22	2.19	0.43
1:B:155:MET:HE3	2:B:1507:CLR:H211	2.01	0.43

Continued on next page...

For Manuscript Review

Continued from previous page...

Atom-1	Atom-2	Interatomic distance (Å)	Clash overlap (Å)
1:B:246:MET:HE1	1:B:1142:TRP:CE2	2.54	0.43
1:B:423:ALA:HB3	1:B:424:PRO:HD3	2.01	0.43
2:B:1507:CLR:H263	2:B:1507:CLR:H231	1.78	0.43
1:A:699:LEU:HA	1:A:755:TYR:CE1	2.54	0.42
1:B:43:GLN:HE21	1:B:117:LYS:HB2	1.84	0.42
1:B:68:LEU:HD22	1:B:133:LYS:HA	2.01	0.42
1:B:478:ILE:HD11	1:B:1021:ALA:HB2	2.01	0.42
1:B:537:THR:HG22	1:B:563:PHE:CD2	2.54	0.42
1:A:467:THR:O	1:A:471:LEU:HD13	2.20	0.42
1:B:402:LEU:HD23	1:B:403:MET:N	2.34	0.42
1:B:425:PHE:O	1:B:429:ILE:HG12	2.19	0.42
1:B:614:ILE:HB	1:B:639:VAL:HB	2.01	0.42
1:B:642:GLY:N	1:B:791:THR:HG22	2.34	0.42
1:B:1437:LEU:O	1:B:1441:ILE:HG13	2.19	0.42
1:A:590:LEU:HA	1:A:593:VAL:HG12	2.01	0.42
1:B:280:ASP:O	1:B:284:GLN:HG2	2.18	0.42
1:B:1077:TYR:CD2	1:B:1143:LEU:HD22	2.55	0.42
1:B:1285:LEU:HD12	1:B:1285:LEU:HA	1.89	0.42
1:A:469:GLU:HB3	1:A:514:TRP:CE3	2.54	0.42
1:A:541:PHE:O	1:A:545:THR:OG1	2.27	0.42
1:B:73:TYR:O	1:B:74:ASN:ND2	2.51	0.42
1:B:174:TYR:O	1:B:175:LEU:C	2.63	0.42
1:B:632:LEU:HD21	1:B:657:SER:HB2	2.00	0.42
1:A:125:MET:O	1:A:128:ILE:HG12	2.20	0.42
1:A:410:LEU:HA	1:A:413:ILE:HG22	2.01	0.42
1:A:1103:LEU:HD13	1:A:1106:LEU:HD11	2.00	0.42
1:A:1231:ARG:HD2	1:A:1231:ARG:HA	1.83	0.42
1:A:1356:HIS:ND1	1:A:1356:HIS:O	2.52	0.42
1:B:9:TYR:CE1	1:B:109:PHE:HB3	2.55	0.42
1:B:75:TYR:CE1	1:B:79:LEU:HD21	2.55	0.42
1:B:957:LEU:CD2	2:B:1503:CLR:H162	2.49	0.42
1:A:403:MET:HE2	1:A:403:MET:N	2.34	0.42
1:A:481:MET:HE3	1:A:481:MET:HA	2.01	0.42
1:B:646:ALA:HB3	1:B:808:ILE:HD13	2.02	0.42
1:B:767:LEU:HD22	1:B:779:PHE:CE1	2.55	0.42
1:B:957:LEU:HB3	2:B:1503:CLR:H152	2.02	0.42
2:B:1504:CLR:H222	2:B:1504:CLR:H25	1.65	0.42
2:A:1504:CLR:H14	2:B:1503:CLR:H161	2.02	0.42
1:A:121:TRP:HZ3	1:A:186:PHE:HD2	1.66	0.42
1:A:173:LEU:HD23	1:A:173:LEU:HA	1.76	0.42
1:A:927:CYS:HB3	1:A:977:ASN:ND2	2.34	0.42

Continued on next page...

For Manuscript Review

Continued from previous page...

Atom-1	Atom-2	Interatomic distance (Å)	Clash overlap (Å)
1:B:26:PHE:HB2	1:B:30:THR:HG23	2.01	0.42
1:B:120:ALA:O	1:B:124:VAL:HG23	2.20	0.42
1:A:957:LEU:HB3	2:A:1505:CLR:C15	2.50	0.42
1:B:242:MET:HB3	1:B:242:MET:HE2	1.79	0.42
1:B:622:SER:HA	1:B:631:THR:H	1.85	0.42
1:B:1019:ARG:O	1:B:1023:ASP:HB2	2.19	0.42
1:A:612:PRO:HA	1:A:640:PRO:HA	2.01	0.41
1:A:642:GLY:N	1:A:791:THR:HG22	2.35	0.41
1:A:1349:TRP:HA	1:A:1349:TRP:CE3	2.55	0.41
1:A:1396:ARG:NH1	1:A:1396:ARG:HG3	2.35	0.41
2:A:1505:CLR:H14	2:B:1507:CLR:H71	2.00	0.41
1:A:423:ALA:HB3	1:A:424:PRO:HD3	2.02	0.41
1:A:786:GLU:HG2	1:A:787:LEU:HG	2.02	0.41
1:A:1118:MET:O	1:A:1121:ILE:HG13	2.21	0.41
1:A:1318:VAL:HG23	1:A:1319:LEU:HD22	2.01	0.41
1:B:491:VAL:HB	1:B:494:TYR:HB2	2.02	0.41
1:B:1336:ASN:HD21	1:B:1372:VAL:HG11	1.85	0.41
1:A:226:CYS:HB3	1:A:260:TRP:CD1	2.55	0.41
1:A:381:ARG:HG2	1:A:385:ARG:HH11	1.84	0.41
1:A:491:VAL:O	1:A:495:ALA:N	2.54	0.41
1:A:1070:PHE:HE1	1:A:1197:LEU:HD12	1.85	0.41
1:A:1396:ARG:HG3	1:A:1396:ARG:HH11	1.84	0.41
1:A:310:ILE:HG13	1:A:421:TRP:CH2	2.56	0.41
1:A:1063:ILE:HD12	1:A:1154:MET:HG3	2.01	0.41
1:A:1118:MET:HE3	1:A:1119:ALA:N	2.35	0.41
1:A:1126:MET:O	1:A:1130:ILE:HG12	2.20	0.41
1:B:22:VAL:HG12	1:B:24:ASN:H	1.85	0.41
1:B:488:MET:HE2	1:B:488:MET:N	2.36	0.41
1:A:1463:LEU:HD23	1:A:1465:SER:H	1.85	0.41
1:B:75:TYR:CD1	1:B:75:TYR:C	2.99	0.41
1:B:388:ASN:H	1:B:391:ARG:NH1	2.19	0.41
1:B:809:VAL:HG22	1:B:819:GLU:HG2	2.02	0.41
1:A:53:ARG:NE	1:A:139:LEU:HD13	2.36	0.41
1:A:484:VAL:HG23	1:A:487:ALA:CB	2.48	0.41
1:A:645:VAL:HG22	1:A:807:ARG:HE	1.86	0.41
1:A:30:THR:O	1:A:34:THR:HG22	2.21	0.41
1:A:65:ARG:HB3	1:A:134:ILE:HG23	2.01	0.41
1:A:401:ASN:HB2	1:A:402:LEU:HD22	2.03	0.41
1:A:534:VAL:O	1:A:537:THR:OG1	2.35	0.41
1:A:1073:ALA:HB2	2:A:1503:CLR:H112	2.03	0.41
1:A:1417:ILE:O	1:A:1421:ILE:HG12	2.20	0.41

Continued on next page...

For Manuscript Review

Continued from previous page...

Atom-1	Atom-2	Interatomic distance (Å)	Clash overlap (Å)
1:B:11:LYS:HD3	1:B:108:PRO:HD2	2.02	0.41
1:B:15:ASN:HA	1:B:19:THR:HG21	2.03	0.41
1:B:780:GLU:OE1	1:B:780:GLU:N	2.53	0.41
1:B:1352:LEU:HD12	1:B:1352:LEU:HA	1.83	0.41
1:B:1069:LEU:HD13	2:B:1506:CLR:H192	2.01	0.41
1:B:1087:MET:HB3	1:B:1132:PHE:CE2	2.56	0.41
1:A:16:GLY:O	1:A:19:THR:HG22	2.21	0.41
1:A:406:ASP:N	1:A:406:ASP:OD1	2.53	0.41
1:A:658:LEU:HD11	1:A:764:ASP:HB3	2.03	0.41
2:A:1503:CLR:H222	2:A:1503:CLR:H162	1.71	0.41
1:B:85:THR:O	1:B:89:LEU:HD23	2.20	0.41
1:B:1080:THR:O	1:B:1084:VAL:HG13	2.21	0.41
1:A:1380:SER:O	1:A:1383:GLN:NE2	2.27	0.40
1:A:1437:LEU:O	1:A:1441:ILE:HG13	2.21	0.40
1:B:76:PHE:HA	1:B:79:LEU:HG	2.03	0.40
1:B:157:LEU:HD23	1:B:157:LEU:HA	1.82	0.40
1:B:262:LEU:HD11	1:B:1130:ILE:HB	2.03	0.40
1:B:640:PRO:HD2	1:B:807:ARG:NH2	2.30	0.40
1:B:721:ASP:OD2	1:B:747:ARG:HD2	2.21	0.40
1:A:386:LEU:HD13	1:A:392:ARG:HH22	1.86	0.40
1:B:133:LYS:HA	1:B:133:LYS:HD2	1.85	0.40
1:B:152:VAL:O	1:B:156:VAL:HG23	2.21	0.40
1:B:386:LEU:HA	1:B:599:ARG:HB2	2.03	0.40
1:B:497:GLU:H	1:B:497:GLU:CD	2.28	0.40
1:B:520:LEU:HA	1:B:520:LEU:HD23	1.78	0.40
1:B:560:LEU:HD23	1:B:560:LEU:HA	1.85	0.40
1:B:590:LEU:O	1:B:594:LEU:HG	2.21	0.40
1:B:763:PHE:HB2	1:B:794:LEU:HD13	2.04	0.40
1:B:1046:LEU:HA	1:B:1046:LEU:HD12	1.85	0.40
1:B:1337:LEU:H	1:B:1337:LEU:HD12	1.86	0.40
1:A:376:ILE:HG21	1:A:1126:MET:SD	2.61	0.40
1:A:1179:MET:HB2	1:A:1179:MET:HE2	1.87	0.40
1:B:68:LEU:HB2	1:B:71:ARG:NH1	2.36	0.40
1:B:485:LEU:HB2	1:B:1013:LEU:HD12	2.04	0.40
1:B:491:VAL:HA	1:B:494:TYR:HB2	2.02	0.40
1:B:693:THR:HG21	1:B:731:THR:O	2.20	0.40
1:B:1101:GLU:HA	1:B:1104:ASN:HD22	1.86	0.40
1:A:921:VAL:O	1:A:925:LEU:HG	2.21	0.40
1:A:1248:VAL:HB	1:A:1297:LYS:HG2	2.03	0.40
1:B:958:PHE:CD1	3:B:1501:R16:H421	2.55	0.40
1:B:1418:GLN:HB3	1:B:1422:ARG:HH12	1.85	0.40

Continued on next page...

For Manuscript Review

Continued from previous page...

Atom-1	Atom-2	Interatomic distance (Å)	Clash overlap (Å)
1:A:589:ARG:NH2	1:A:590:LEU:HD23	2.35	0.40
1:A:774:VAL:HA	1:A:777:GLN:HG2	2.04	0.40
1:A:1113:LYS:HE2	1:A:1113:LYS:HB2	1.91	0.40

There are no symmetry-related clashes.

5.3 Torsion angles (i)

5.3.1 Protein backbone (i)

In the following table, the Percentiles column shows the percent Ramachandran outliers of the chain as a percentile score with respect to all PDB entries followed by that with respect to all EM entries.

The Analysed column shows the number of residues for which the backbone conformation was analysed, and the total number of residues.

Mol	Chain	Analysed	Favoured	Allowed	Outliers	Percentiles	
1	A	1373/1464 (94%)	1314 (96%)	59 (4%)	0	100	100
1	B	1373/1464 (94%)	1318 (96%)	55 (4%)	0	100	100
All	All	2746/2928 (94%)	2632 (96%)	114 (4%)	0	100	100

There are no Ramachandran outliers to report.

5.3.2 Protein sidechains (i)

In the following table, the Percentiles column shows the percent sidechain outliers of the chain as a percentile score with respect to all PDB entries followed by that with respect to all EM entries.

The Analysed column shows the number of residues for which the sidechain conformation was analysed, and the total number of residues.

Mol	Chain	Analysed	Rotameric	Outliers	Percentiles	
1	A	1193/1264 (94%)	1123 (94%)	70 (6%)	16	41
1	B	1193/1264 (94%)	1136 (95%)	57 (5%)	21	48
All	All	2386/2528 (94%)	2259 (95%)	127 (5%)	21	44

All (127) residues with a non-rotameric sidechain are listed below:

For Manuscript Review

Mol	Chain	Res	Type
1	A	6	ILE
1	A	53	ARG
1	A	72	LEU
1	A	75	TYR
1	A	79	LEU
1	A	80	LEU
1	A	89	LEU
1	A	91	ARG
1	A	98	VAL
1	A	128	ILE
1	A	151	LEU
1	A	169	SER
1	A	172	VAL
1	A	173	LEU
1	A	175	LEU
1	A	179	GLU
1	A	271	LEU
1	A	323	LEU
1	A	355	LEU
1	A	357	GLU
1	A	404	THR
1	A	443	LEU
1	A	449	LEU
1	A	478	ILE
1	A	485	LEU
1	A	488	MET
1	A	489	ASP
1	A	490	THR
1	A	493	CYS
1	A	494	TYR
1	A	512	LEU
1	A	519	GLN
1	A	524	LEU
1	A	526	MET
1	A	552	THR
1	A	570	LEU
1	A	576	ILE
1	A	580	VAL
1	A	584	ASN
1	A	665	GLU
1	A	691	ASN
1	A	720	HIS
1	A	749	SER

Continued on next page...

For Manuscript Review

Continued from previous page...

Mol	Chain	Res	Type
1	A	907	VAL
1	A	919	TRP
1	A	1002	ARG
1	A	1008	PHE
1	A	1009	HIS
1	A	1022	LYS
1	A	1024	LEU
1	A	1112	TYR
1	A	1189	ILE
1	A	1194	THR
1	A	1197	LEU
1	A	1199	LEU
1	A	1219	ILE
1	A	1255	LEU
1	A	1266	ILE
1	A	1293	VAL
1	A	1318	VAL
1	A	1333	VAL
1	A	1342	GLU
1	A	1347	ASP
1	A	1349	TRP
1	A	1351	SER
1	A	1352	LEU
1	A	1359	ASP
1	A	1401	VAL
1	A	1430	MET
1	A	1442	ASP
1	B	5	PHE
1	B	42	SER
1	B	63	VAL
1	B	68	LEU
1	B	74	ASN
1	B	80	LEU
1	B	102	ASP
1	B	114	LEU
1	B	151	LEU
1	B	162	LEU
1	B	167	TYR
1	B	169	SER
1	B	171	TYR
1	B	172	VAL
1	B	258	ASP

Continued on next page...

For Manuscript Review

Continued from previous page...

Mol	Chain	Res	Type
1	B	271	LEU
1	B	371	LEU
1	B	382	LYS
1	B	398	LYS
1	B	418	HIS
1	B	447	LEU
1	B	494	TYR
1	B	512	LEU
1	B	528	ILE
1	B	529	LEU
1	B	552	THR
1	B	568	PHE
1	B	596	THR
1	B	700	PHE
1	B	720	HIS
1	B	774	VAL
1	B	1009	HIS
1	B	1015	ARG
1	B	1024	LEU
1	B	1027	ILE
1	B	1104	ASN
1	B	1108	THR
1	B	1112	TYR
1	B	1189	ILE
1	B	1193	LEU
1	B	1194	THR
1	B	1197	LEU
1	B	1226	VAL
1	B	1227	ILE
1	B	1228	GLU
1	B	1229	ASN
1	B	1230	ASN
1	B	1258	VAL
1	B	1310	PHE
1	B	1314	ASP
1	B	1315	LEU
1	B	1342	GLU
1	B	1371	GLU
1	B	1372	VAL
1	B	1388	SER
1	B	1431	LEU
1	B	1440	ILE

For Manuscript Review

Sometimes sidechains can be flipped to improve hydrogen bonding and reduce clashes. All (33) such sidechains are listed below:

Mol	Chain	Res	Type
1	A	21	GLN
1	A	193	HIS
1	A	296	ASN
1	A	316	GLN
1	A	324	ASN
1	A	363	ASN
1	A	482	ASN
1	A	746	GLN
1	A	912	GLN
1	A	1009	HIS
1	A	1011	ASN
1	A	1260	HIS
1	A	1455	GLN
1	A	1462	ASN
1	B	74	ASN
1	B	232	ASN
1	B	316	GLN
1	B	363	ASN
1	B	582	ASN
1	B	584	ASN
1	B	697	ASN
1	B	776	GLN
1	B	970	GLN
1	B	1041	GLN
1	B	1128	ASN
1	B	1165	GLN
1	B	1260	HIS
1	B	1287	ASN
1	B	1336	ASN
1	B	1356	HIS
1	B	1364	ASN
1	B	1455	GLN
1	B	1462	ASN

5.3.3 RNA (i)

There are no RNA molecules in this entry.

For Manuscript Review

5.4 Non-standard residues in protein, DNA, RNA chains (i)

There are no non-standard protein/DNA/RNA residues in this entry.

5.5 Carbohydrates (i)

There are no oligosaccharides in this entry.

5.6 Ligand geometry (i)

12 ligands are modelled in this entry.

In the following table, the Counts columns list the number of bonds (or angles) for which Mogul statistics could be retrieved, the number of bonds (or angles) that are observed in the model and the number of bonds (or angles) that are defined in the Chemical Component Dictionary. The Link column lists molecule types, if any, to which the group is linked. The Z score for a bond length (or angle) is the number of standard deviations the observed value is removed from the expected value. A bond length (or angle) with $|Z| > 2$ is considered an outlier worth inspection. RMSZ is the root-mean-square of all Z scores of the bond lengths (or angles).

Mol	Type	Chain	Res	Link	Bond lengths			Bond angles		
					Counts	RMSZ	#|Z| > 2	Counts	RMSZ	#|Z| > 2
2	CLR	B	1507	-	31,31,31	0.28	0	48,48,48	0.36	0
2	CLR	A	1503	-	31,31,31	0.31	0	48,48,48	0.49	0
3	R16	B	1502	-	15,15,15	0.23	0	14,14,14	0.45	0
2	CLR	B	1506	-	31,31,31	0.34	0	48,48,48	0.53	0
2	CLR	A	1501	-	31,31,31	0.30	0	48,48,48	0.42	0
2	CLR	A	1502	-	31,31,31	0.30	0	48,48,48	0.42	0
2	CLR	B	1503	-	31,31,31	0.35	0	48,48,48	0.66	0
3	R16	B	1501	-	15,15,15	0.24	0	14,14,14	0.42	0
2	CLR	B	1505	-	31,31,31	0.31	0	48,48,48	0.44	0
2	CLR	A	1504	-	31,31,31	0.28	0	48,48,48	0.47	0
2	CLR	B	1504	-	31,31,31	0.31	0	48,48,48	0.40	0
2	CLR	A	1505	-	31,31,31	0.33	0	48,48,48	0.54	0

In the following table, the Chirals column lists the number of chiral outliers, the number of chiral centers analysed, the number of these observed in the model and the number defined in the Chemical Component Dictionary. Similar counts are reported in the Torsion and Rings columns. '-' means no outliers of that kind were identified.

Mol	Type	Chain	Res	Link	Chirals	Torsions	Rings
2	CLR	B	1507	-	-	10/10/68/68	0/4/4/4
2	CLR	A	1503	-	-	5/10/68/68	0/4/4/4

Continued on next page...

For Manuscript Review

Continued from previous page...

Mol	Type	Chain	Res	Link	Chirals	Torsions	Rings
3	R16	B	1502	-	-	6/13/13/13	-
2	CLR	B	1506	-	-	10/10/68/68	0/4/4/4
2	CLR	A	1501	-	-	8/10/68/68	0/4/4/4
2	CLR	A	1502	-	-	9/10/68/68	0/4/4/4
2	CLR	B	1503	-	-	5/10/68/68	0/4/4/4
3	R16	B	1501	-	-	6/13/13/13	-
2	CLR	B	1505	-	-	8/10/68/68	0/4/4/4
2	CLR	A	1504	-	-	10/10/68/68	0/4/4/4
2	CLR	B	1504	-	-	8/10/68/68	0/4/4/4
2	CLR	A	1505	-	-	5/10/68/68	0/4/4/4

There are no bond length outliers.

There are no bond angle outliers.

There are no chirality outliers.

All (90) torsion outliers are listed below:

Mol	Chain	Res	Type	Atoms
2	A	1502	CLR	C13-C17-C20-C21
2	A	1505	CLR	C13-C17-C20-C21
2	A	1505	CLR	C13-C17-C20-C22
2	A	1505	CLR	C16-C17-C20-C22
2	B	1503	CLR	C13-C17-C20-C21
2	B	1503	CLR	C16-C17-C20-C22
2	B	1505	CLR	C13-C17-C20-C21
2	B	1505	CLR	C16-C17-C20-C22
2	B	1506	CLR	C13-C17-C20-C22
2	A	1504	CLR	C21-C20-C22-C23
2	B	1505	CLR	C21-C20-C22-C23
2	A	1501	CLR	C16-C17-C20-C21
2	A	1502	CLR	C16-C17-C20-C21
2	A	1503	CLR	C16-C17-C20-C21
2	A	1505	CLR	C16-C17-C20-C21
2	B	1503	CLR	C16-C17-C20-C21
2	B	1505	CLR	C16-C17-C20-C21
2	A	1501	CLR	C13-C17-C20-C21
2	A	1503	CLR	C13-C17-C20-C21
2	B	1507	CLR	C13-C17-C20-C21
2	B	1503	CLR	C13-C17-C20-C22
2	B	1505	CLR	C13-C17-C20-C22

Continued on next page...

For Manuscript Review

Continued from previous page...

Mol	Chain	Res	Type	Atoms
2	A	1502	CLR	C21-C20-C22-C23
2	B	1506	CLR	C21-C20-C22-C23
2	B	1507	CLR	C21-C20-C22-C23
2	B	1507	CLR	C16-C17-C20-C21
2	A	1502	CLR	C16-C17-C20-C22
2	B	1506	CLR	C16-C17-C20-C22
2	A	1501	CLR	C13-C17-C20-C22
2	A	1502	CLR	C13-C17-C20-C22
2	A	1503	CLR	C13-C17-C20-C22
2	A	1504	CLR	C13-C17-C20-C22
2	B	1504	CLR	C13-C17-C20-C22
2	B	1507	CLR	C13-C17-C20-C22
2	B	1504	CLR	C16-C17-C20-C21
2	B	1506	CLR	C16-C17-C20-C21
2	B	1504	CLR	C13-C17-C20-C21
2	A	1501	CLR	C16-C17-C20-C22
2	A	1503	CLR	C16-C17-C20-C22
2	B	1507	CLR	C16-C17-C20-C22
2	A	1504	CLR	C13-C17-C20-C21
2	A	1501	CLR	C21-C20-C22-C23
2	B	1504	CLR	C17-C20-C22-C23
2	B	1507	CLR	C17-C20-C22-C23
2	B	1504	CLR	C21-C20-C22-C23
2	A	1504	CLR	C16-C17-C20-C21
2	B	1506	CLR	C13-C17-C20-C21
2	B	1504	CLR	C16-C17-C20-C22
2	A	1502	CLR	C22-C23-C24-C25
2	A	1501	CLR	C17-C20-C22-C23
2	A	1502	CLR	C17-C20-C22-C23
2	B	1505	CLR	C17-C20-C22-C23
2	B	1506	CLR	C17-C20-C22-C23
2	B	1507	CLR	C20-C22-C23-C24
2	B	1507	CLR	C22-C23-C24-C25
2	A	1504	CLR	C16-C17-C20-C22
2	A	1504	CLR	C22-C23-C24-C25
2	B	1506	CLR	C22-C23-C24-C25
2	B	1503	CLR	C20-C22-C23-C24
2	A	1505	CLR	C20-C22-C23-C24
2	B	1506	CLR	C23-C24-C25-C27
3	B	1501	R16	C35-C36-C37-C38
2	B	1507	CLR	C23-C24-C25-C27
3	B	1501	R16	C36-C37-C38-C39

Continued on next page...

For Manuscript Review

Continued from previous page...

Mol	Chain	Res	Type	Atoms
3	B	1502	R16	C35-C36-C37-C38
3	B	1502	R16	C31-C32-C33-C34
2	B	1506	CLR	C23-C24-C25-C26
2	B	1505	CLR	C20-C22-C23-C24
2	B	1505	CLR	C22-C23-C24-C25
3	B	1501	R16	C39-C40-C41-C42
3	B	1501	R16	C27-C28-C29-C30
3	B	1502	R16	C37-C38-C39-C40
3	B	1502	R16	C34-C35-C36-C37
2	B	1504	CLR	C22-C23-C24-C25
3	B	1502	R16	C39-C40-C41-C42
2	A	1502	CLR	C23-C24-C25-C26
2	B	1506	CLR	C20-C22-C23-C24
2	A	1504	CLR	C20-C22-C23-C24
2	A	1502	CLR	C23-C24-C25-C27
3	B	1502	R16	C38-C39-C40-C41
2	A	1504	CLR	C23-C24-C25-C27
2	A	1504	CLR	C17-C20-C22-C23
3	B	1501	R16	C32-C33-C34-C35
2	B	1507	CLR	C23-C24-C25-C26
2	B	1504	CLR	C20-C22-C23-C24
3	B	1501	R16	C37-C38-C39-C40
2	A	1504	CLR	C23-C24-C25-C26
2	A	1501	CLR	C20-C22-C23-C24
2	A	1503	CLR	C22-C23-C24-C25
2	A	1501	CLR	C22-C23-C24-C25

There are no ring outliers.

12 monomers are involved in 61 short contacts:

Mol	Chain	Res	Type	Clashes	Symm-Clashes
2	B	1507	CLR	8	0
2	A	1503	CLR	3	0
3	B	1502	R16	9	0
2	B	1506	CLR	2	0
2	A	1501	CLR	2	0
2	A	1502	CLR	2	0
2	B	1503	CLR	14	0
3	B	1501	R16	7	0
2	B	1505	CLR	3	0
2	A	1504	CLR	6	0
2	B	1504	CLR	3	0

Continued on next page...

For Manuscript Review

Continued from previous page...

Mol	Chain	Res	Type	Clashes	Symm-Clashes
2	A	1505	CLR	9	0

The following is a two-dimensional graphical depiction of Mogul quality analysis of bond lengths, bond angles, torsion angles, and ring geometry for all instances of the Ligand of Interest. In addition, ligands with molecular weight > 250 and outliers as shown on the validation Tables will also be included. For torsion angles, if less then 5% of the Mogul distribution of torsion angles is within 10 degrees of the torsion angle in question, then that torsion angle is considered an outlier. Any bond that is central to one or more torsion angles identified as an outlier by Mogul will be highlighted in the graph. For rings, the root-mean-square deviation (RMSD) between the ring in question and similar rings identified by Mogul is calculated over all ring torsion angles. If the average RMSD is greater than 60 degrees and the minimal RMSD between the ring in question and any Mogul-identified rings is also greater than 60 degrees, then that ring is considered an outlier. The outliers are highlighted in purple. The color gray indicates Mogul did not find sufficient equivalents in the CSD to analyse the geometry.

Ligand CLR B 1507

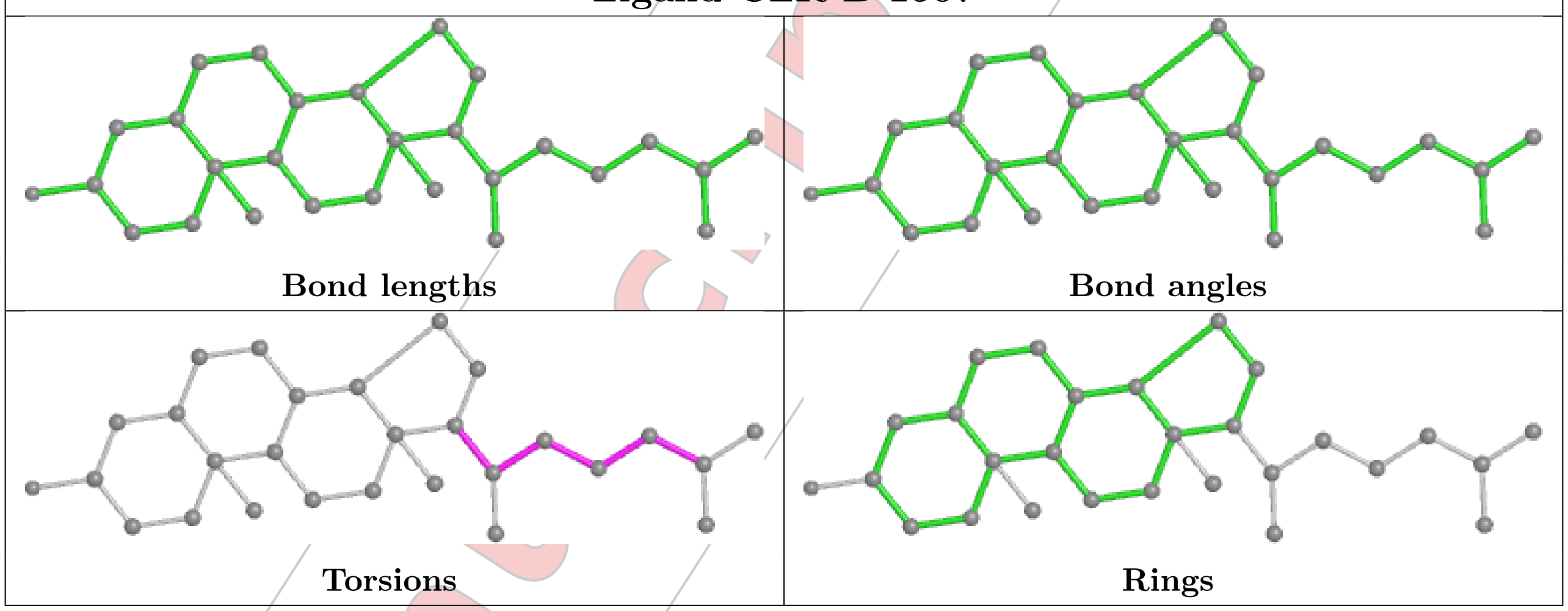

Ligand CLR A 1503

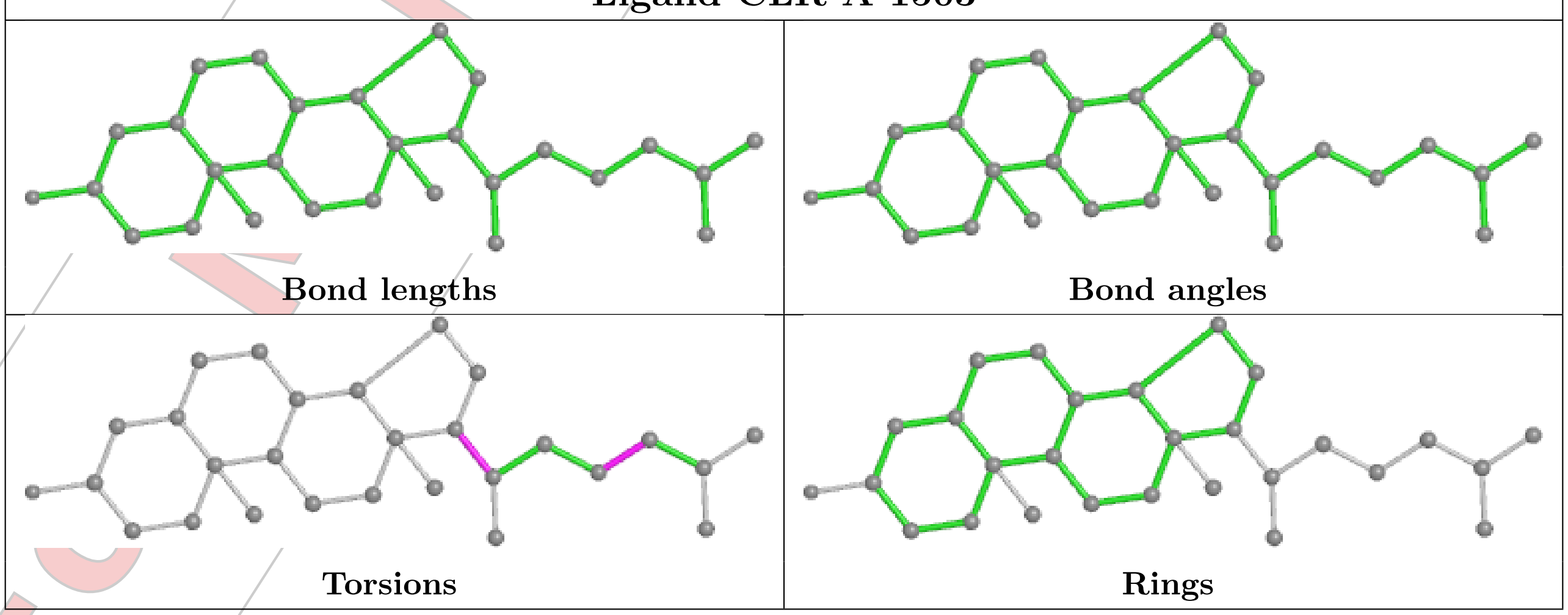

For Manuscript Review

Ligand CLR B 1506

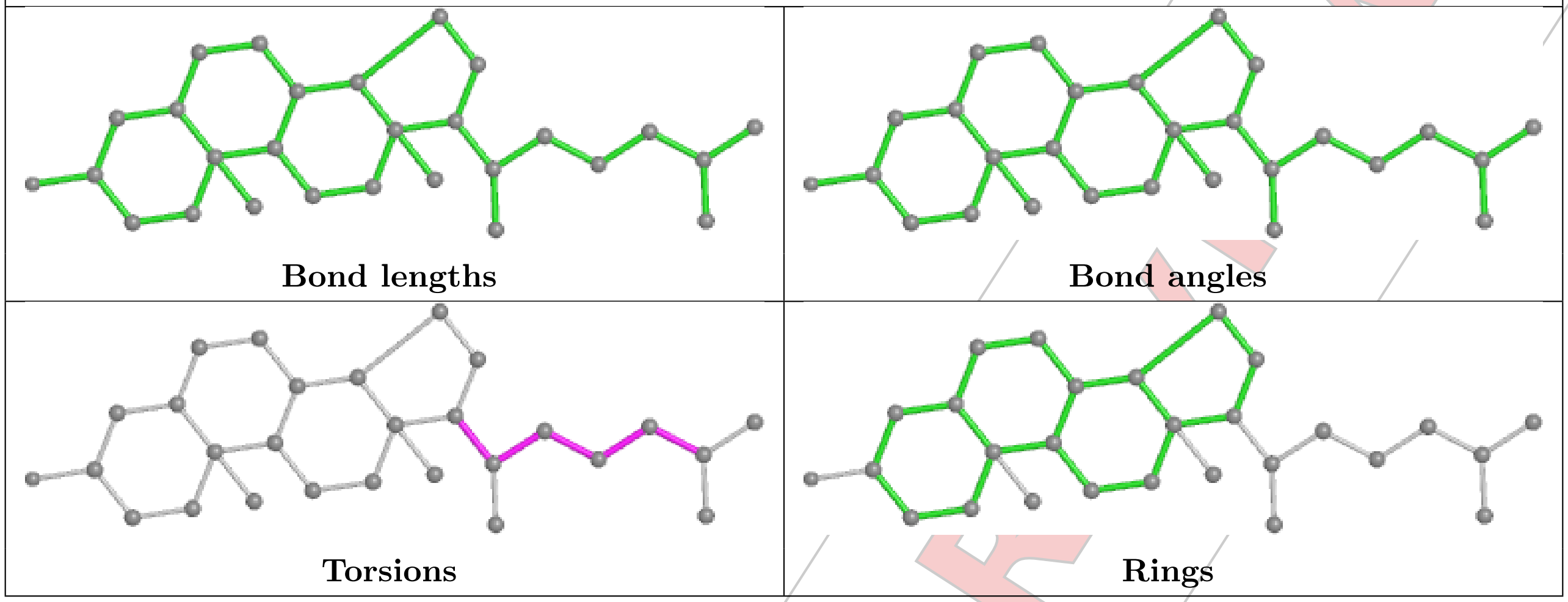

Ligand CLR A 1501

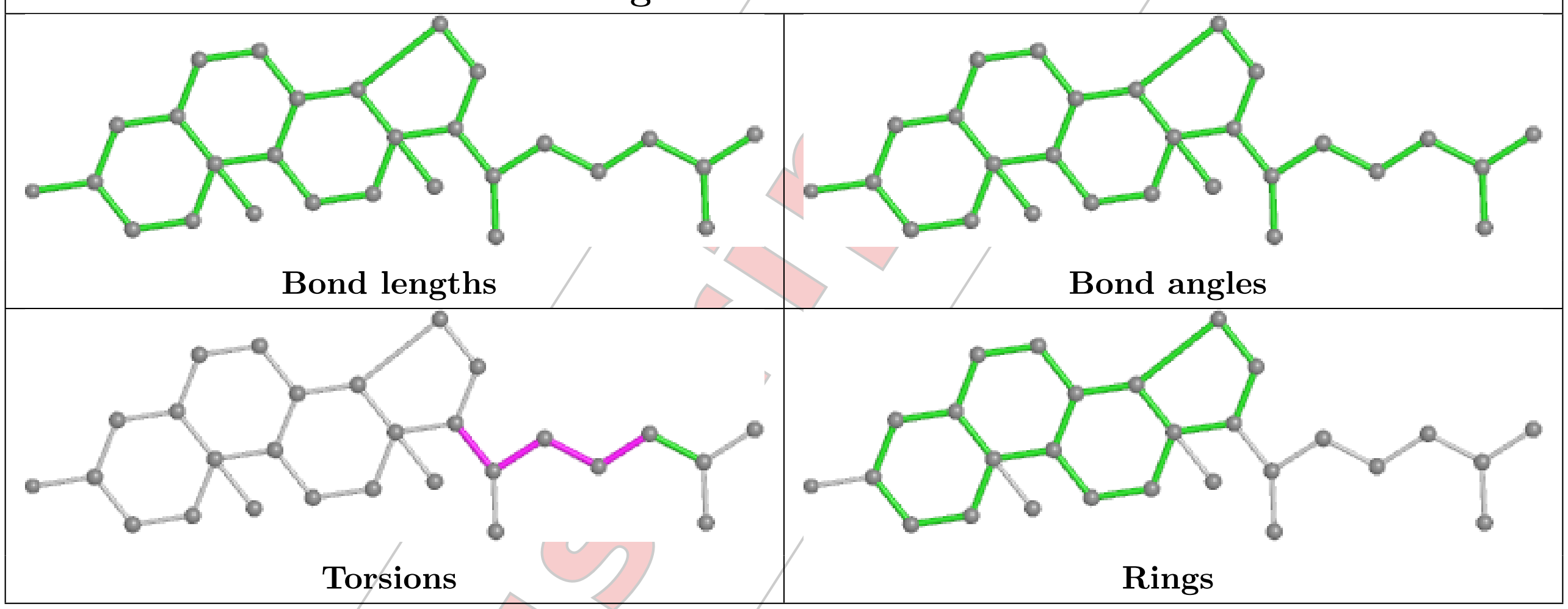

Ligand CLR A 1502

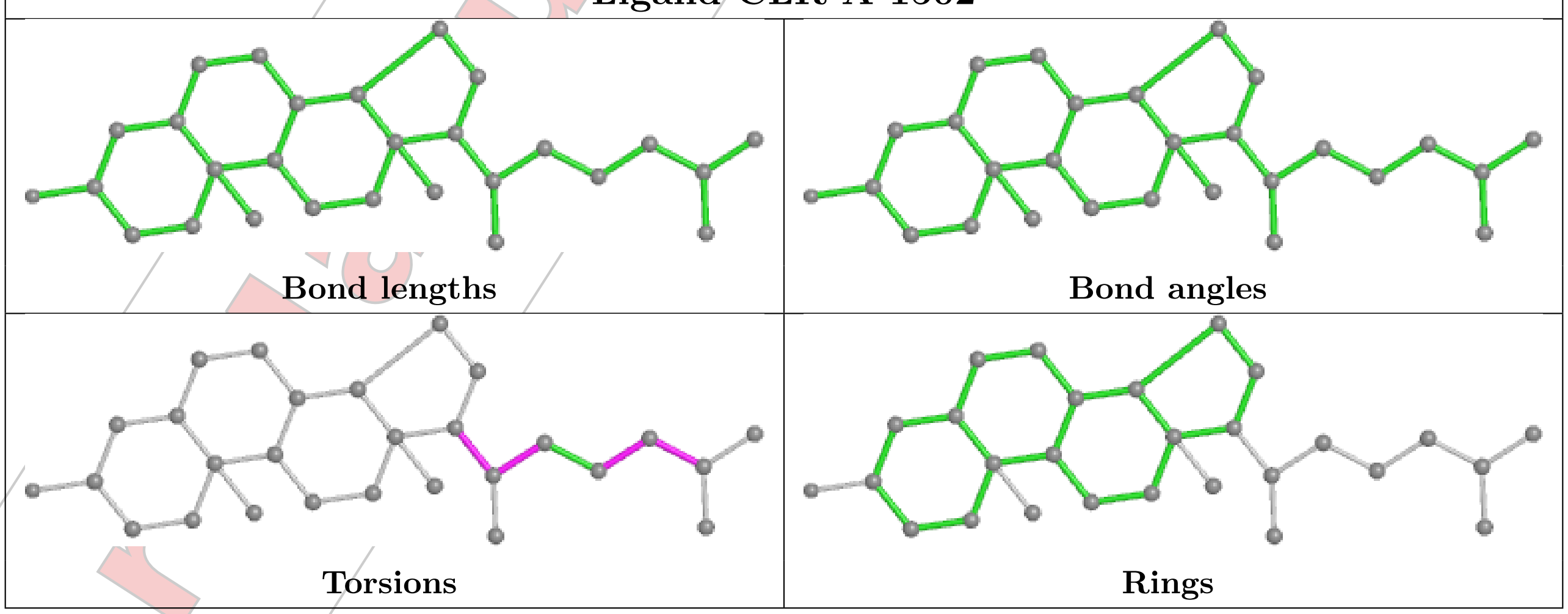

For Manuscript Review

Ligand CLR B 1503

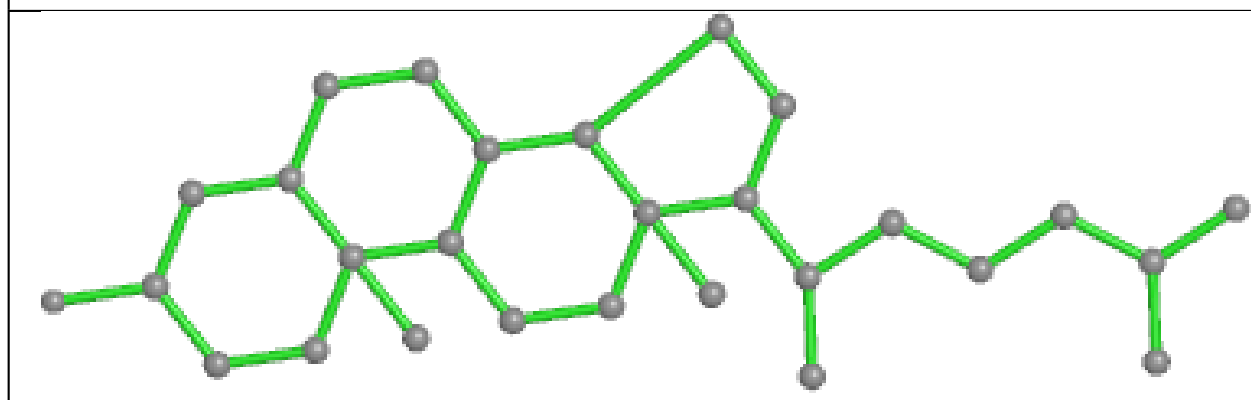
Bond lengths

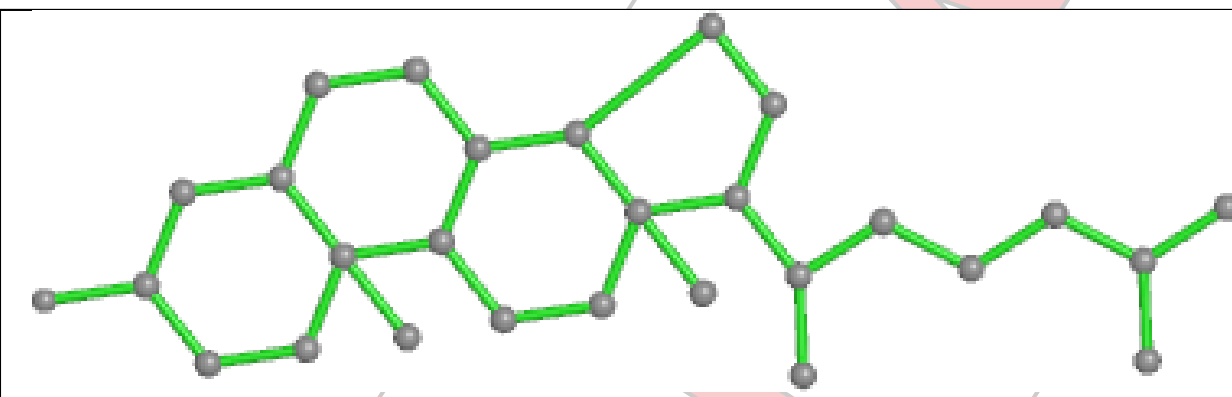
Bond angles

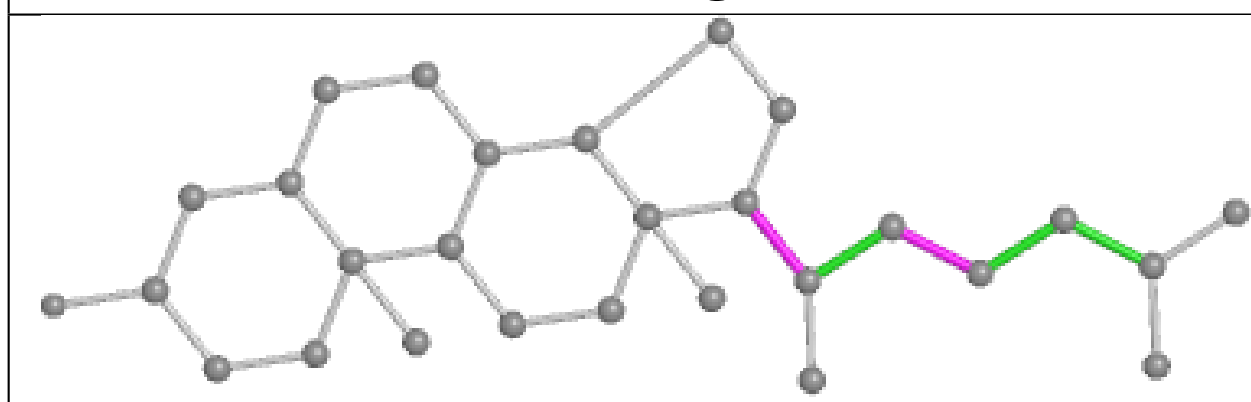
Torsions

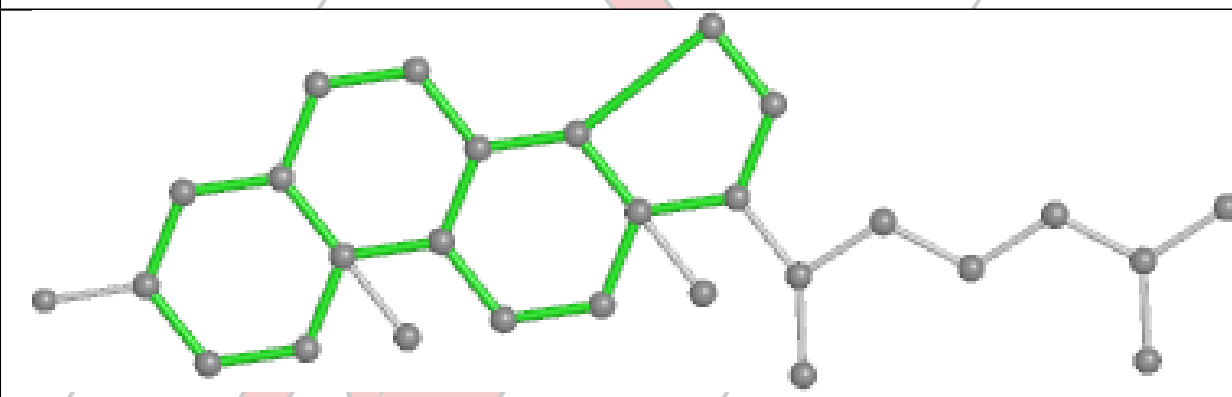
Rings

Ligand CLR B 1505

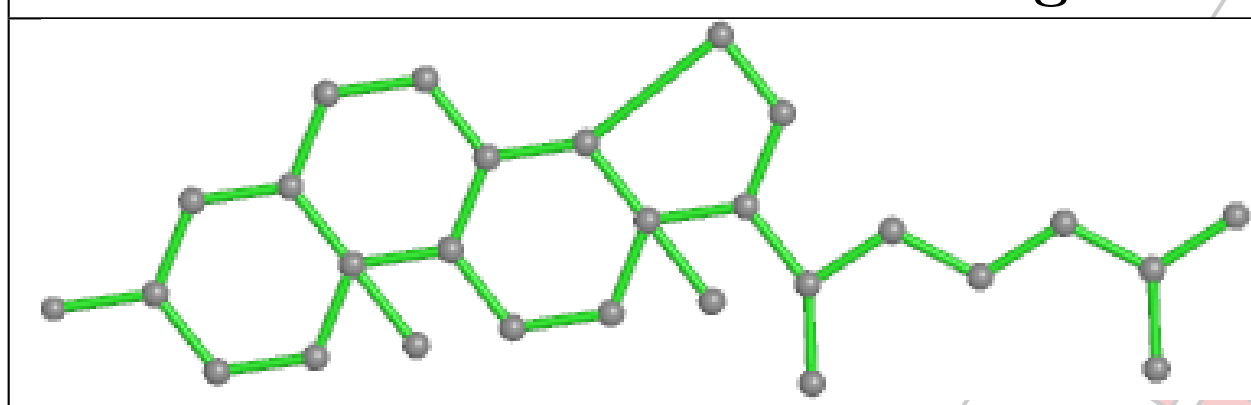
Bond lengths

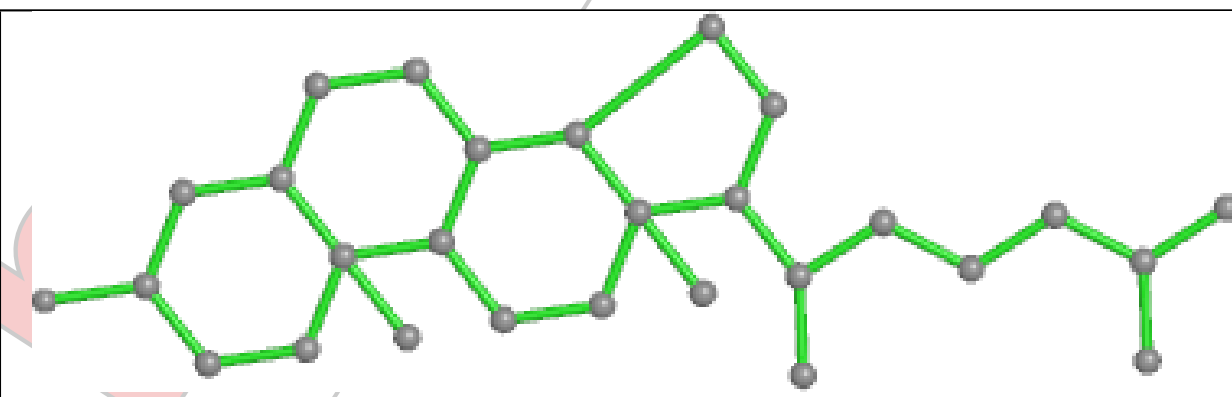
Bond angles

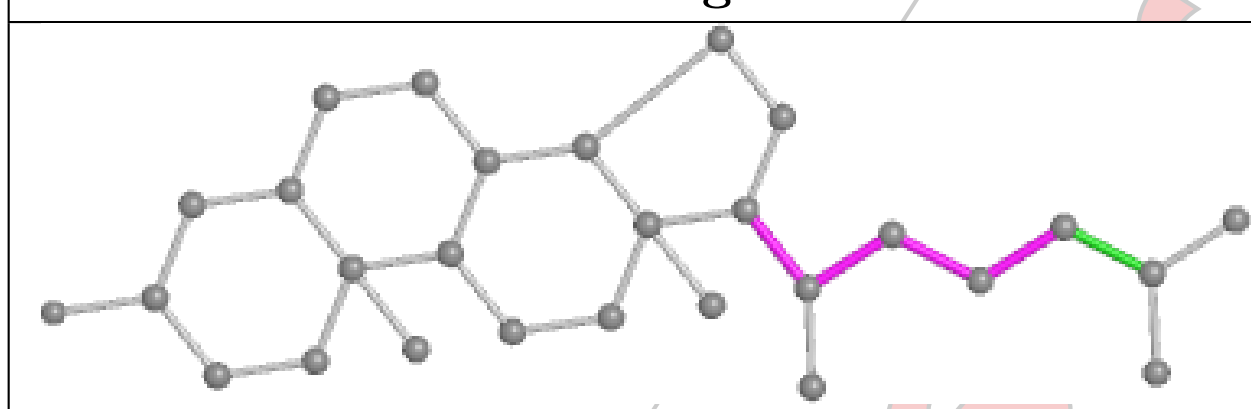
Torsions

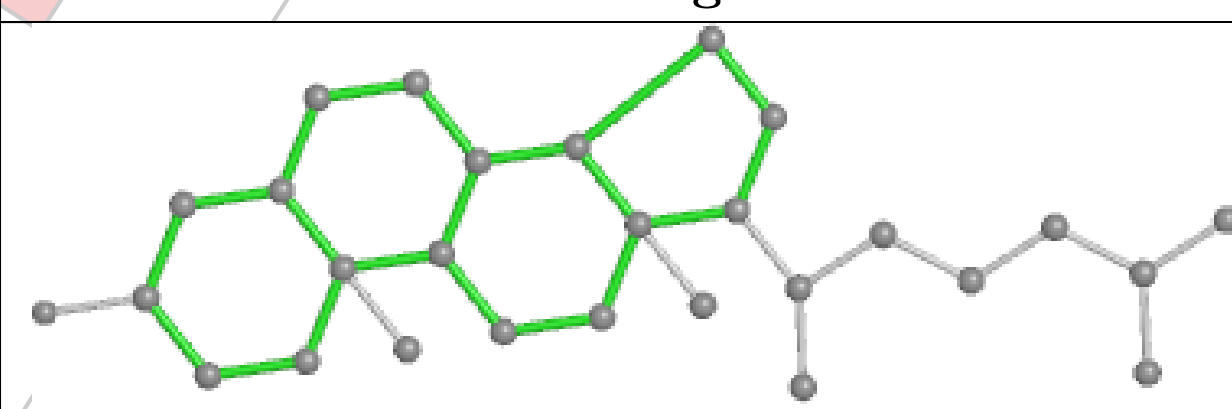
Rings

Ligand CLR A 1504

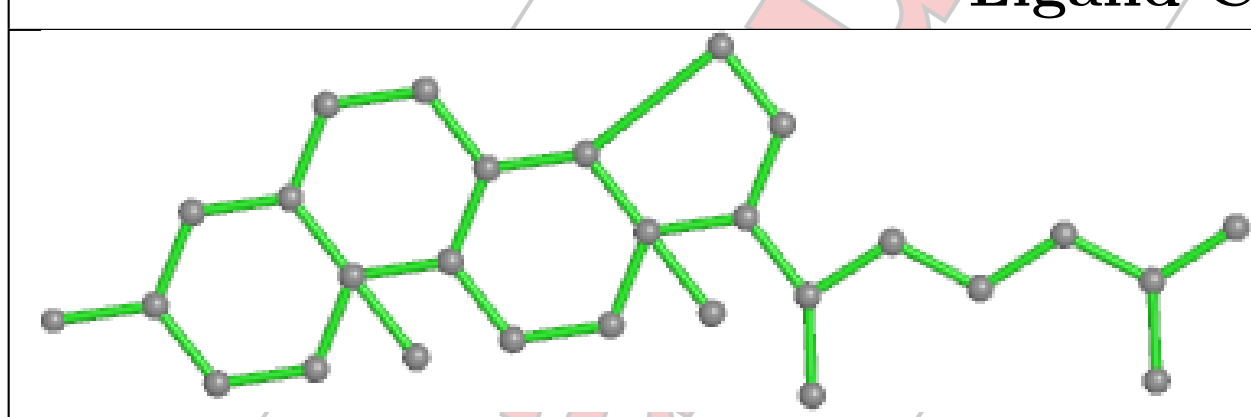
Bond lengths

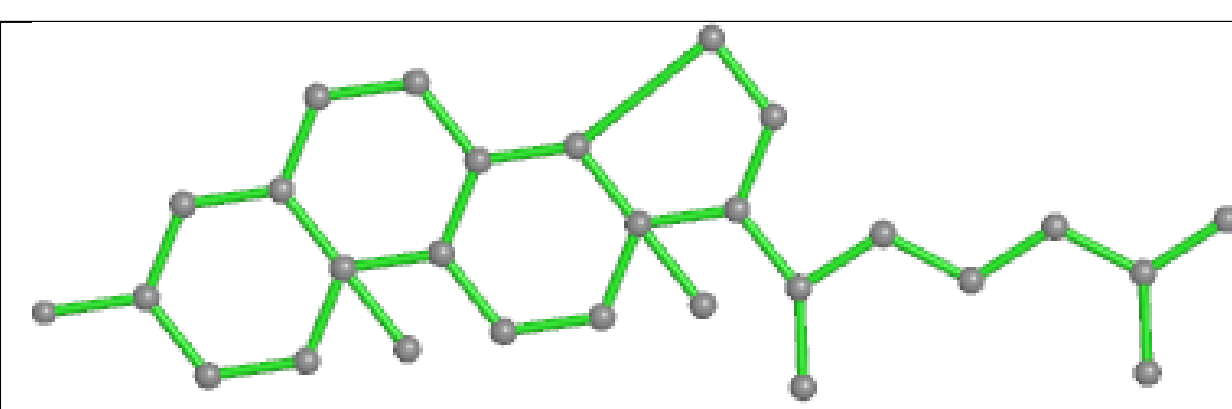
Bond angles

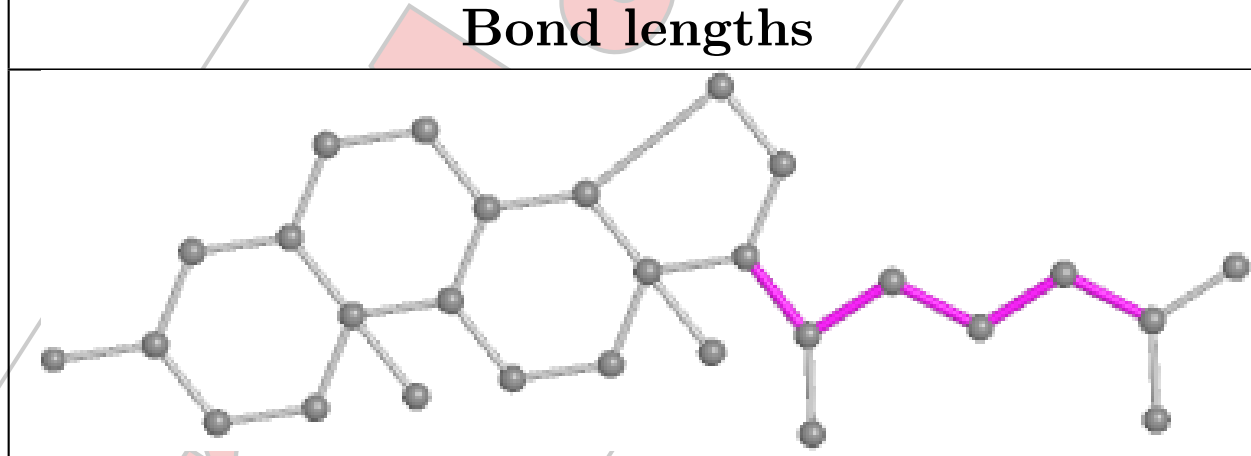
Torsions

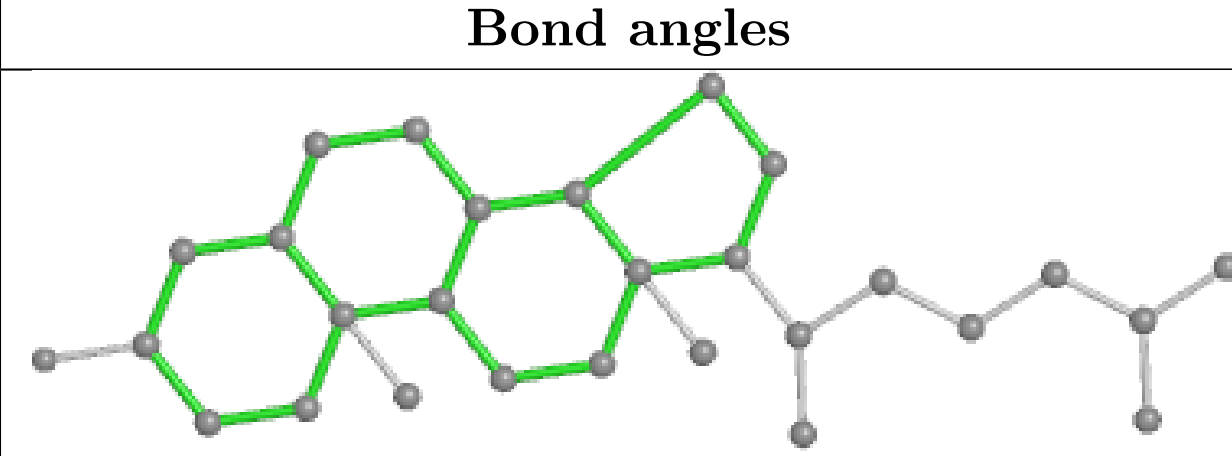
Rings

For Manuscript Review

Ligand CLR B 1504

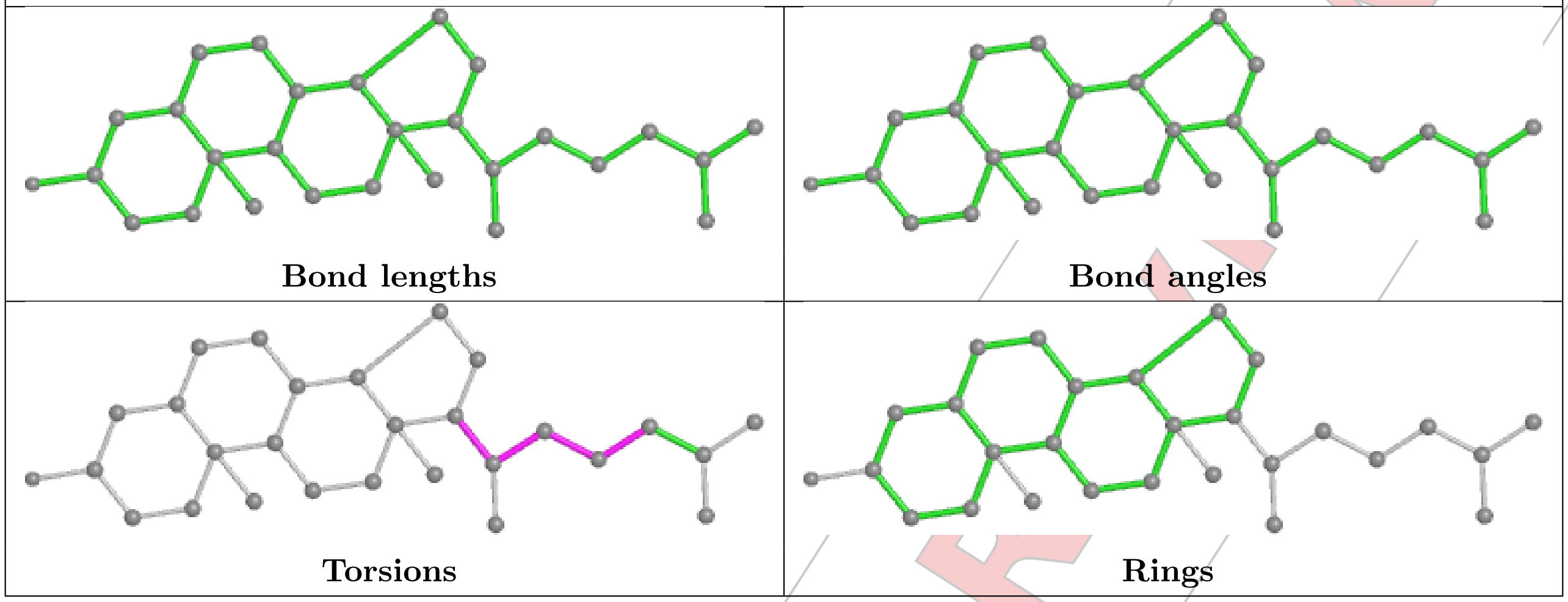

Ligand CLR A 1505

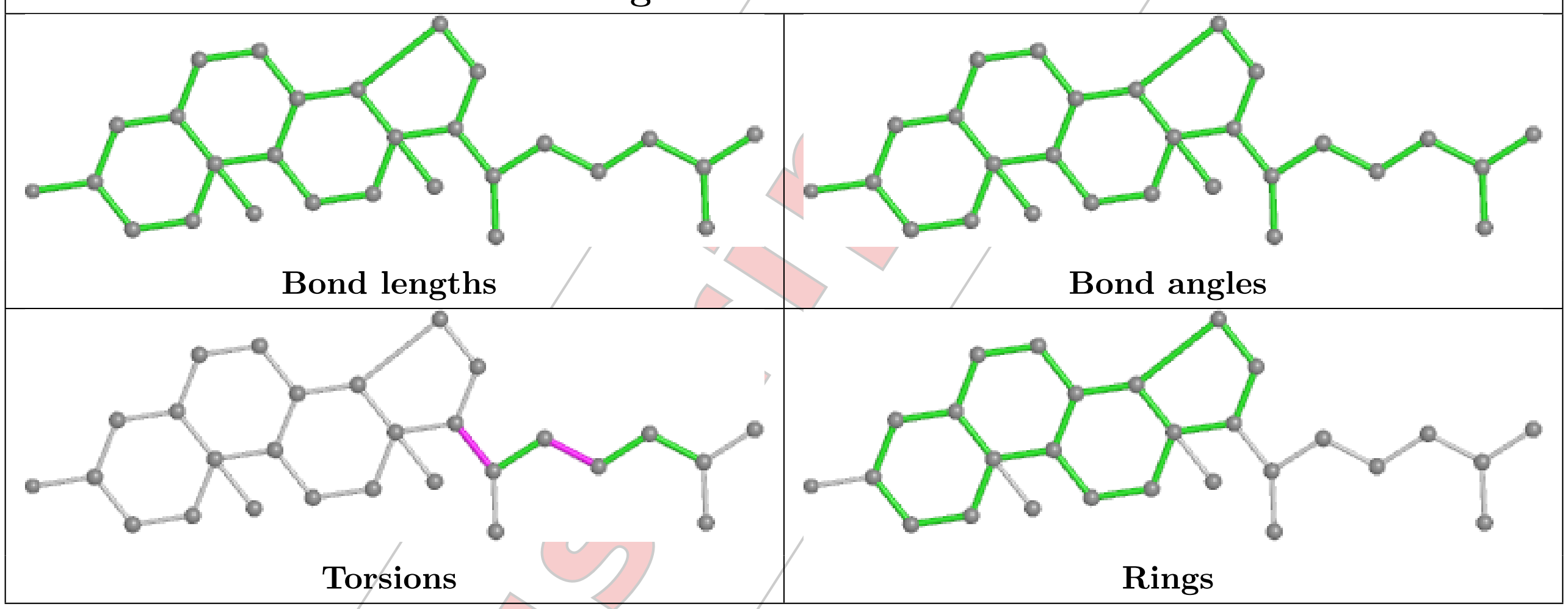

5.7 Other polymers (i)

There are no such residues in this entry.

5.8 Polymer linkage issues (i)

There are no chain breaks in this entry.

For Manuscript Review

6 Map visualisation (i)

This section contains visualisations of the EMDB entry EMD-63865. These allow visual inspection of the internal detail of the map and identification of artifacts.

Images derived from a raw map, generated by summing the deposited half-maps, are presented below the corresponding image components of the primary map to allow further visual inspection and comparison with those of the primary map.

6.1 Orthogonal projections (i)

6.1.1 Primary map

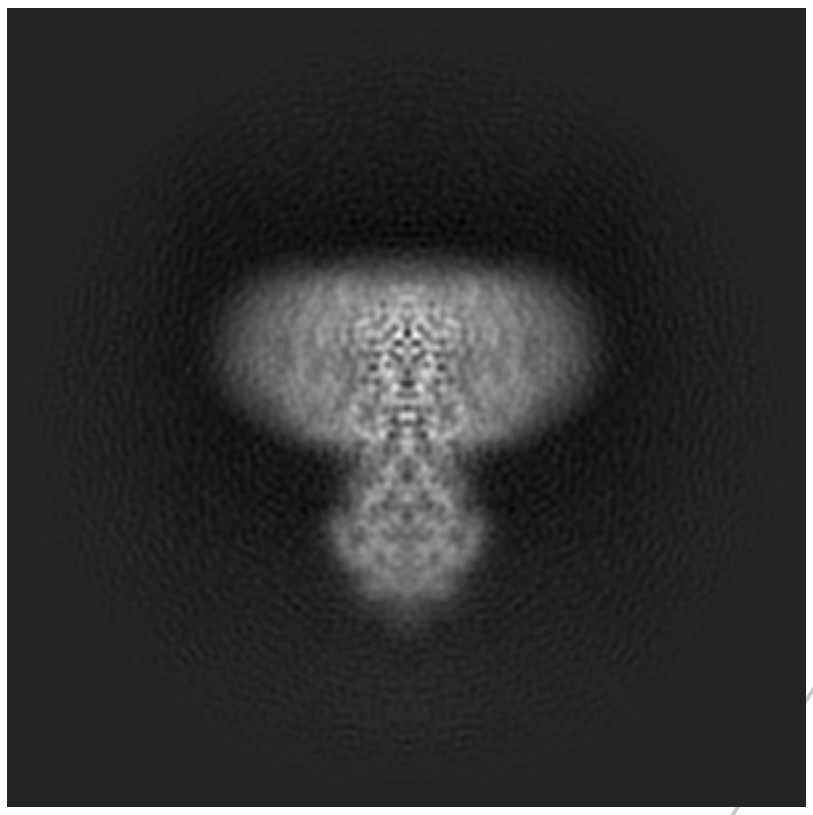
X

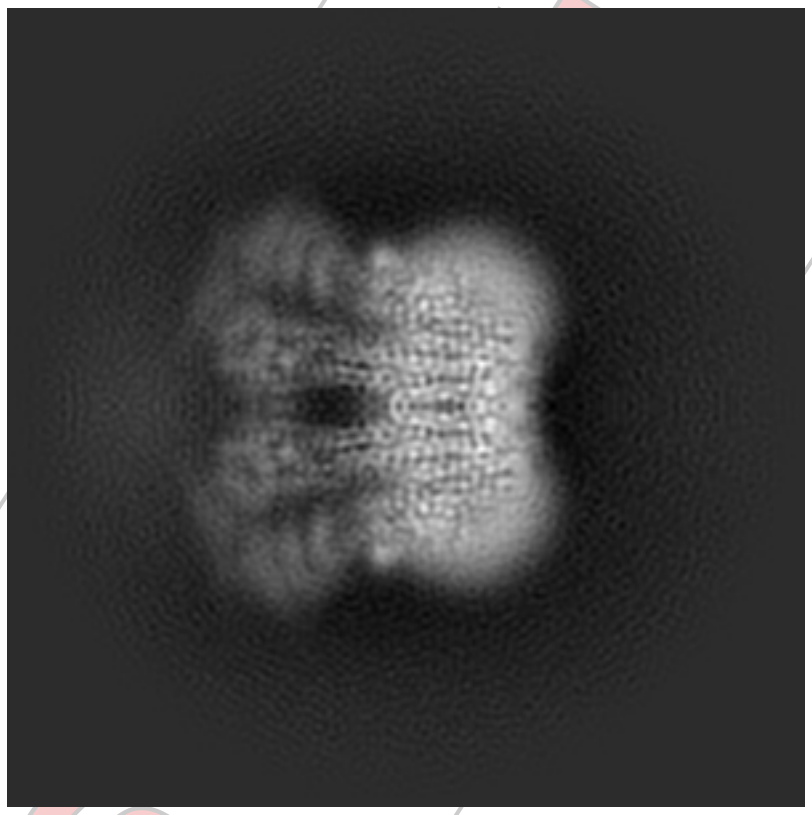
Y

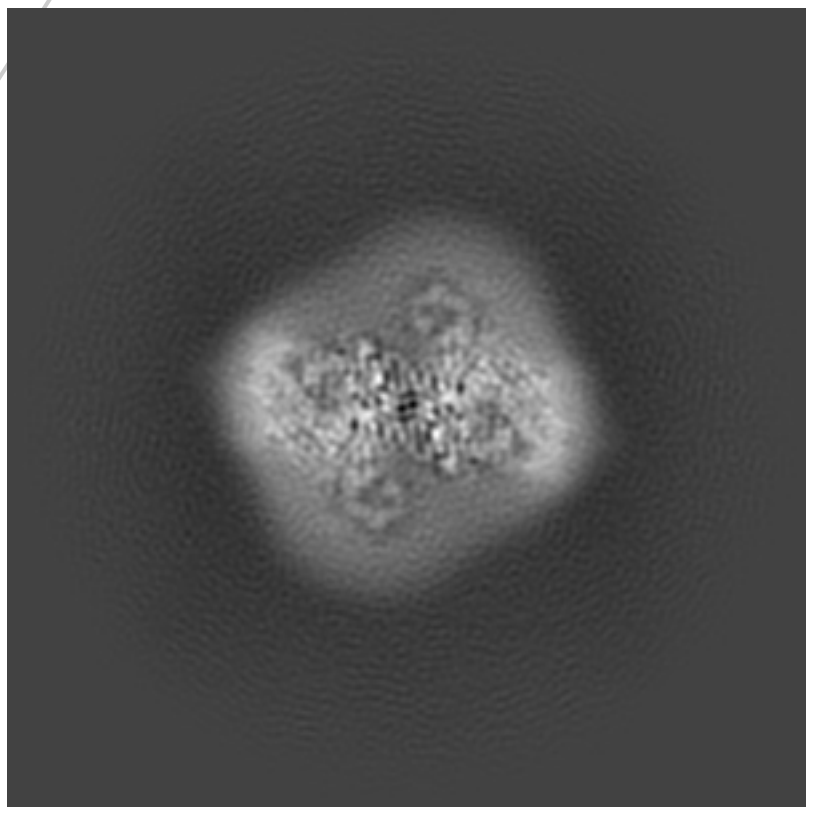
Z

6.1.2 Raw map

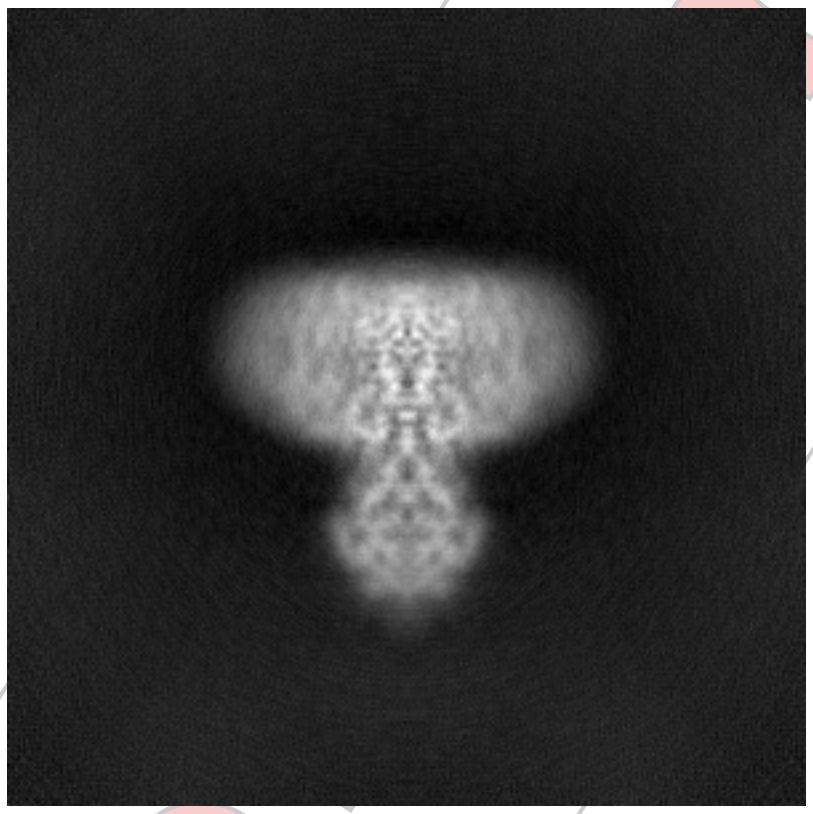
X

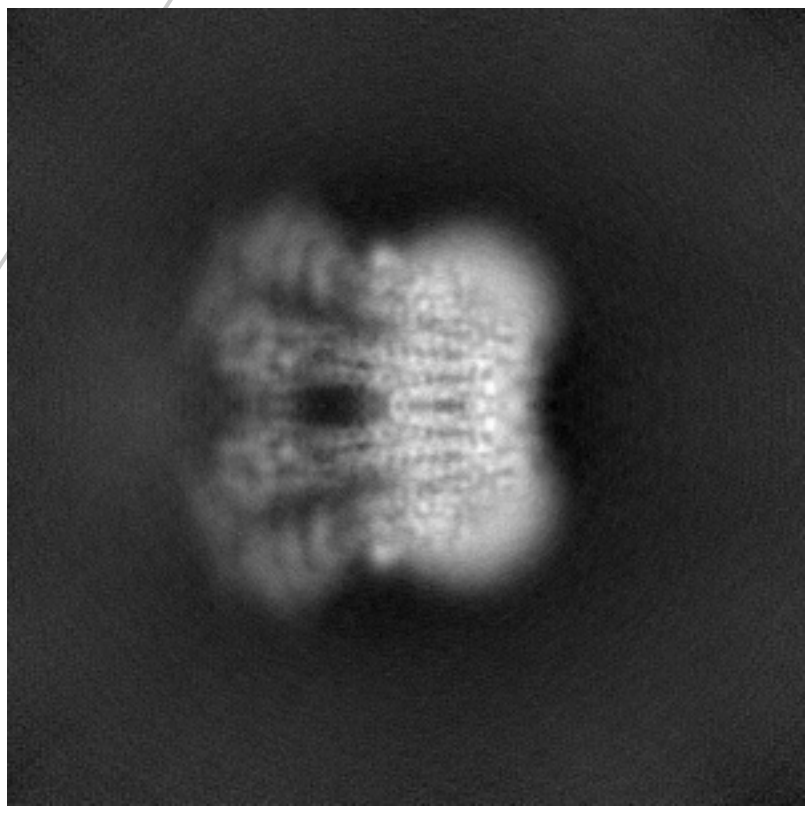
Y

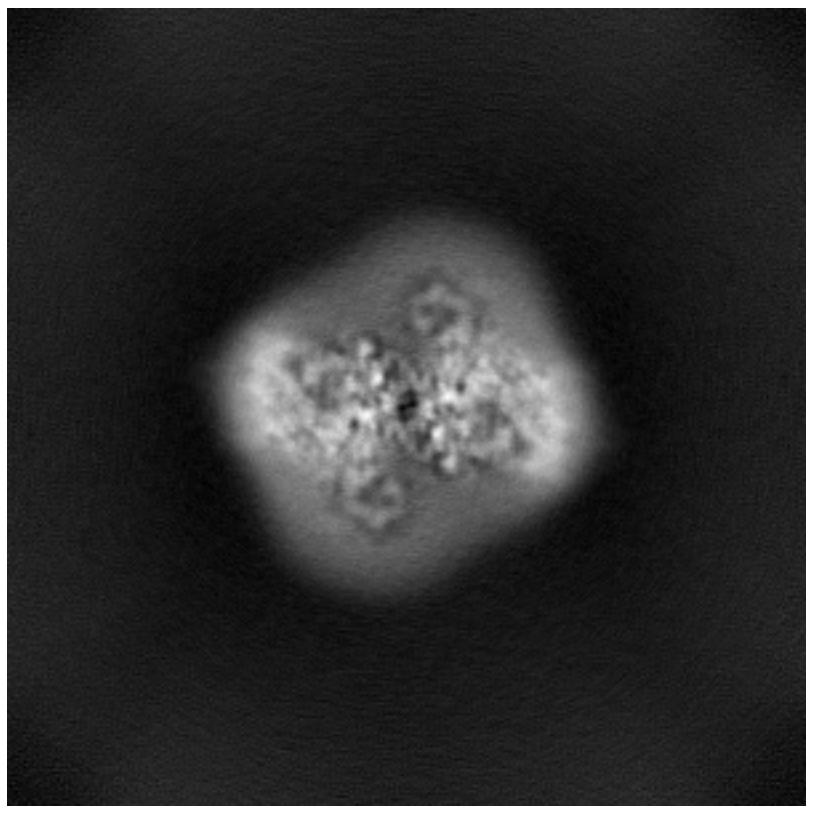
Z

The images above show the map projected in three orthogonal directions.

For Manuscript Review

6.2 Central slices (i)

6.2.1 Primary map

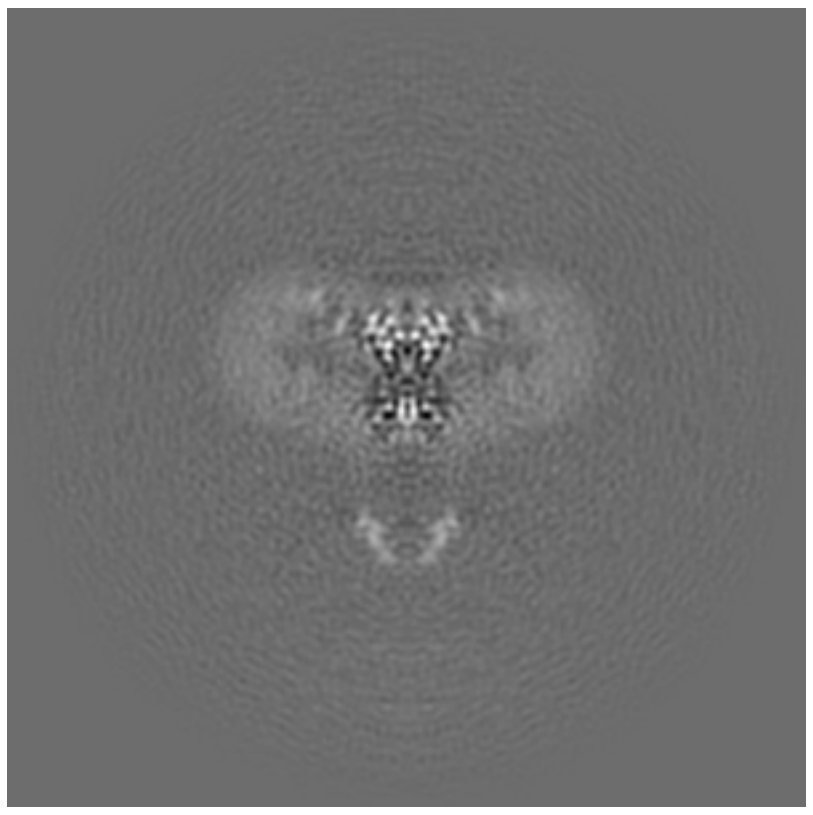

X Index: 200

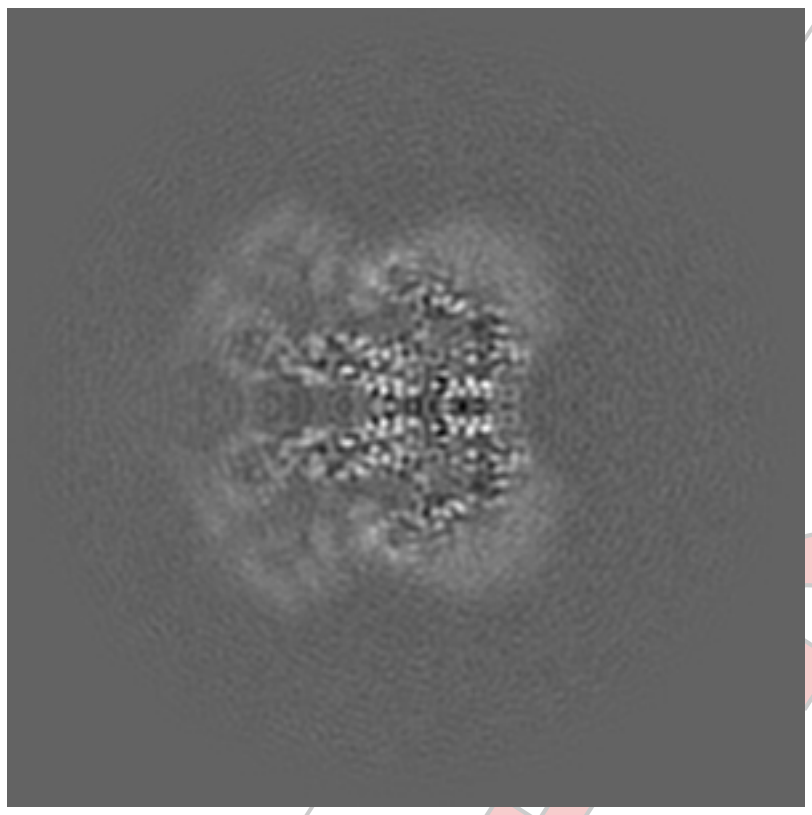

Y Index: 200

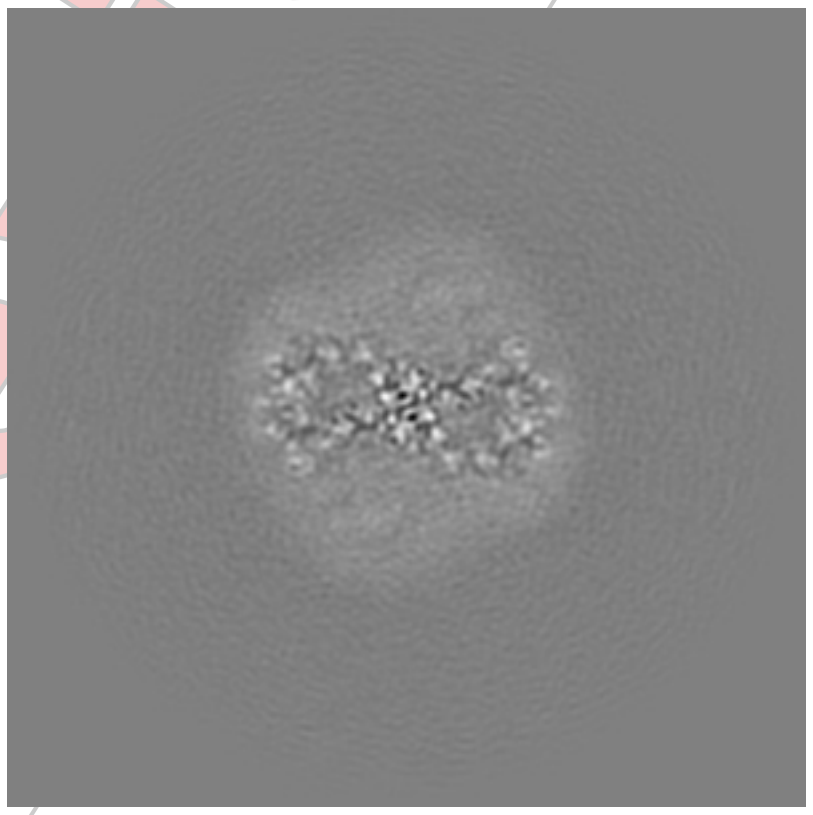

Z Index: 200

6.2.2 Raw map

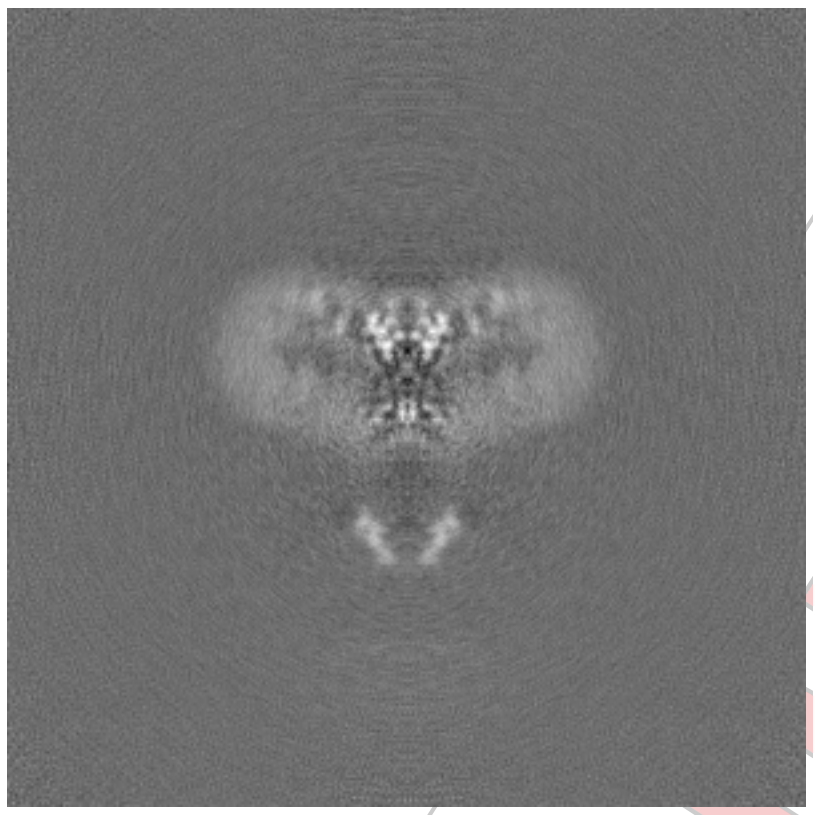

X Index: 200

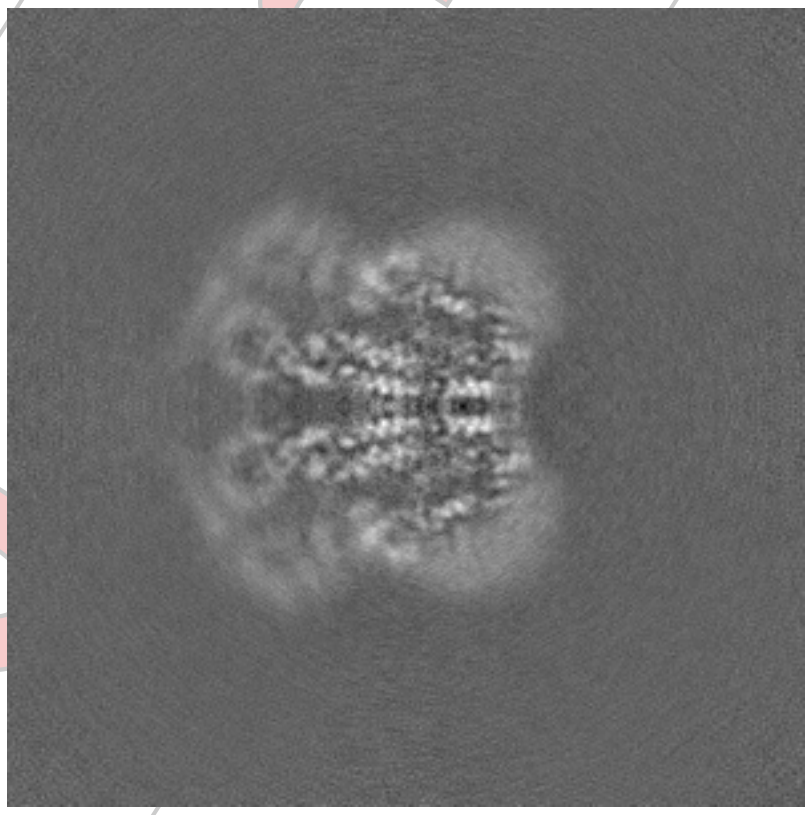

Y Index: 200

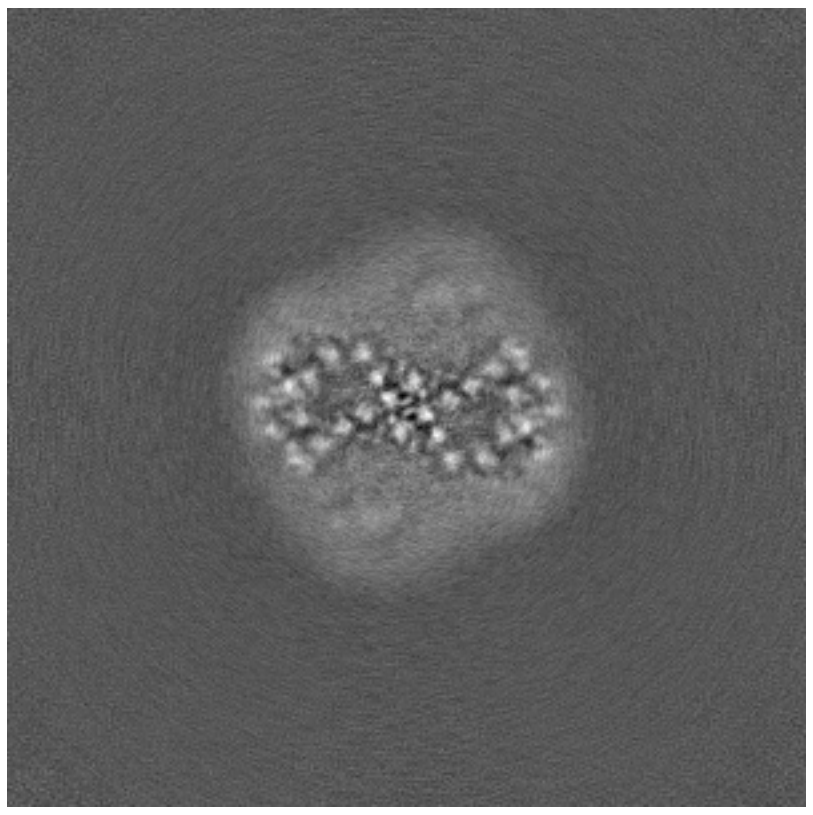

Z Index: 200

The images above show central slices of the map in three orthogonal directions.

For Manuscript Review

6.3 Largest variance slices (i)

6.3.1 Primary map

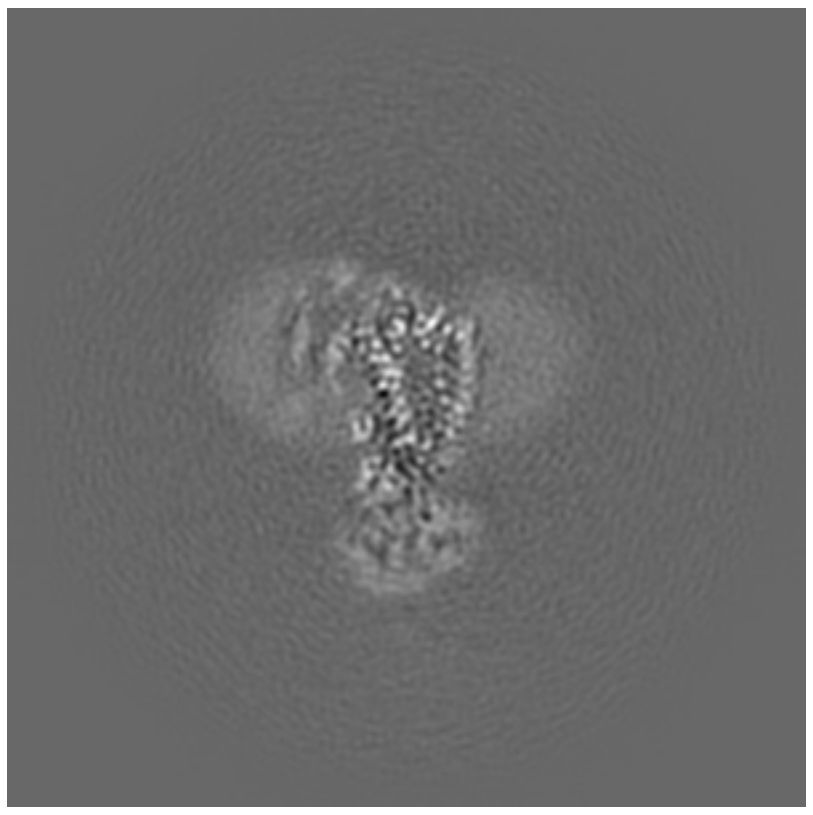
X Index: 179

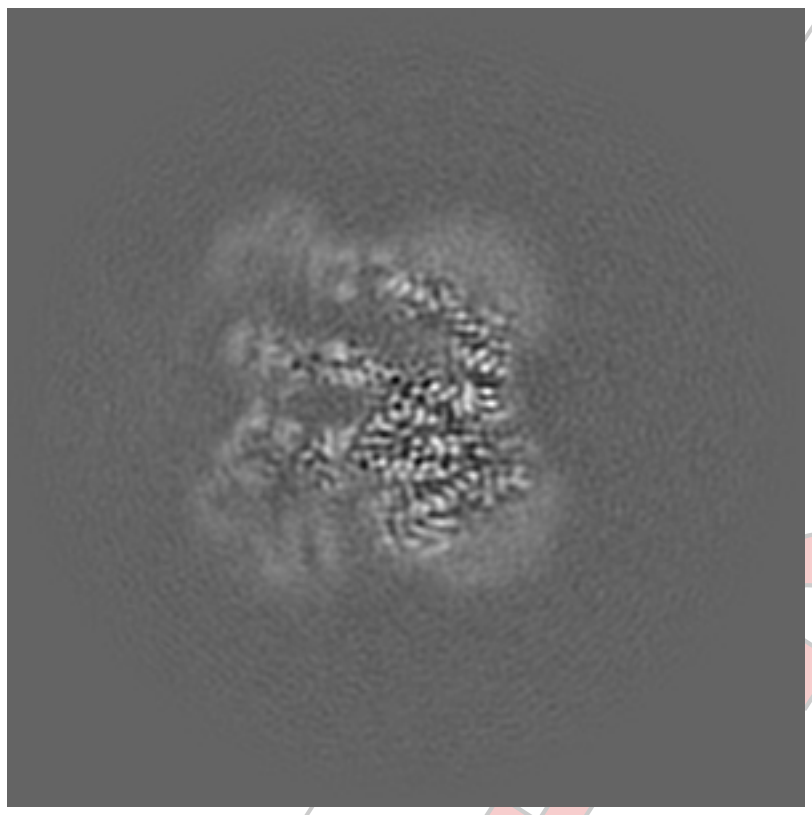
Y Index: 190

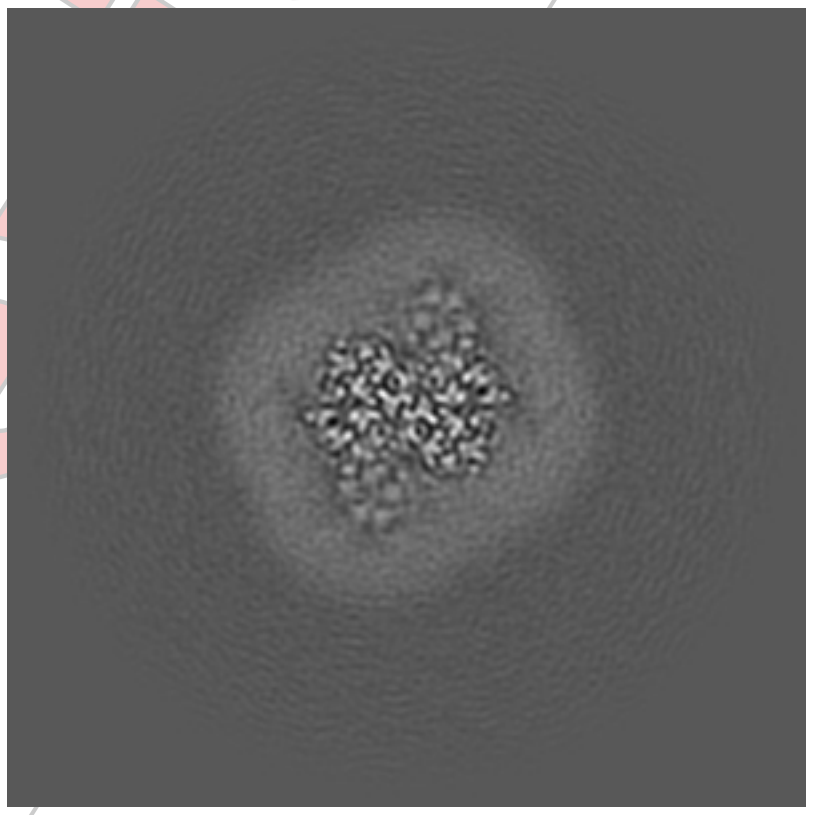
Z Index: 236

6.3.2 Raw map

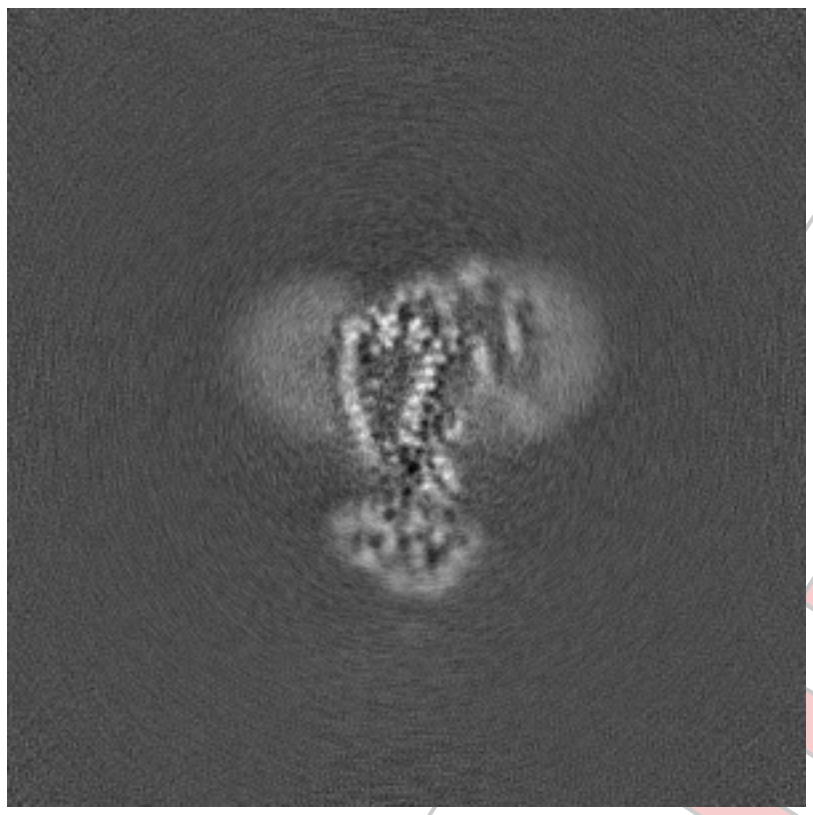
X Index: 222

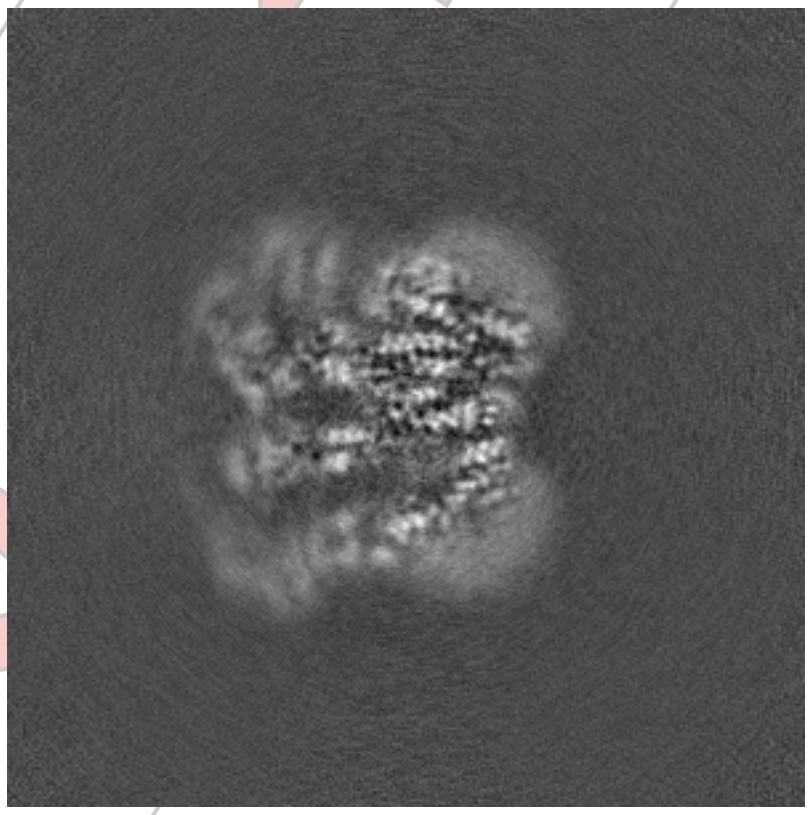
Y Index: 209

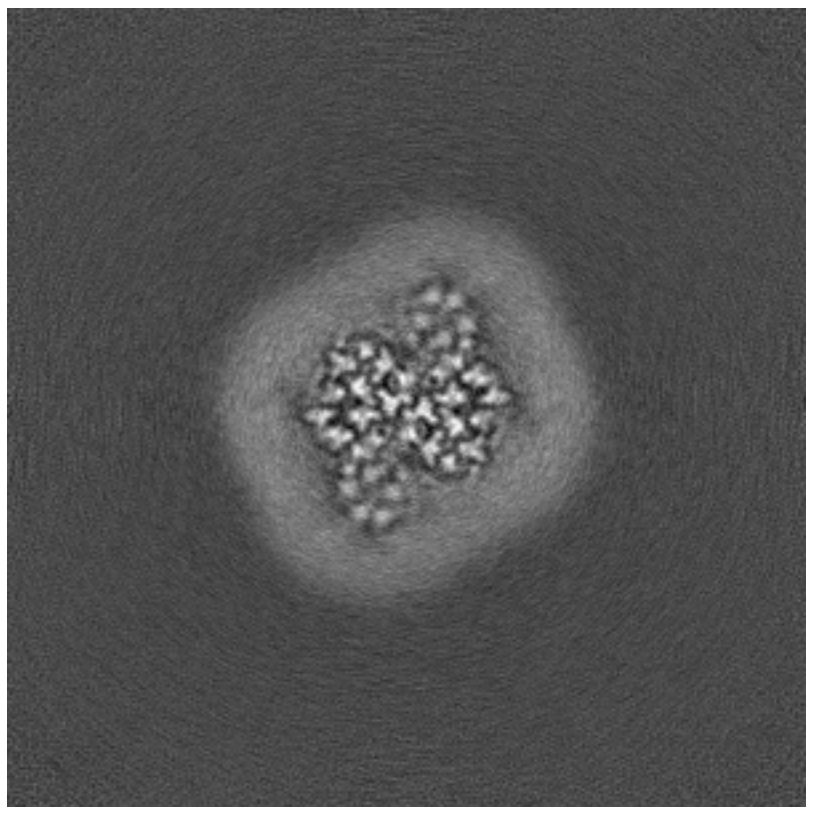
Z Index: 236

The images above show the largest variance slices of the map in three orthogonal directions.

For Manuscript Review

6.4 Orthogonal standard-deviation projections (False-color) ⓘ

6.4.1 Primary map

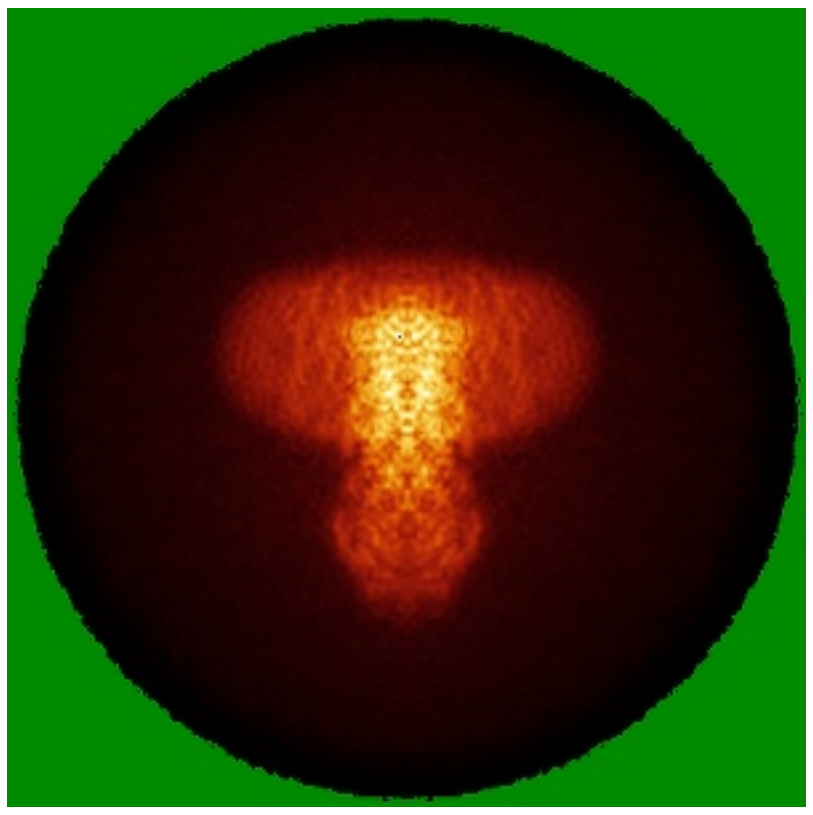
X

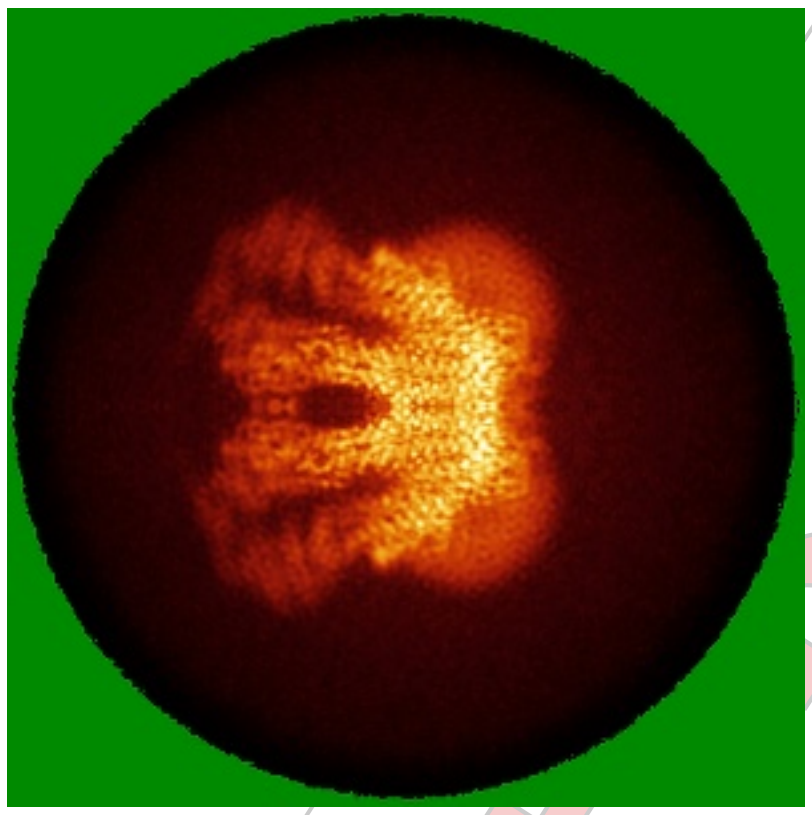
Y

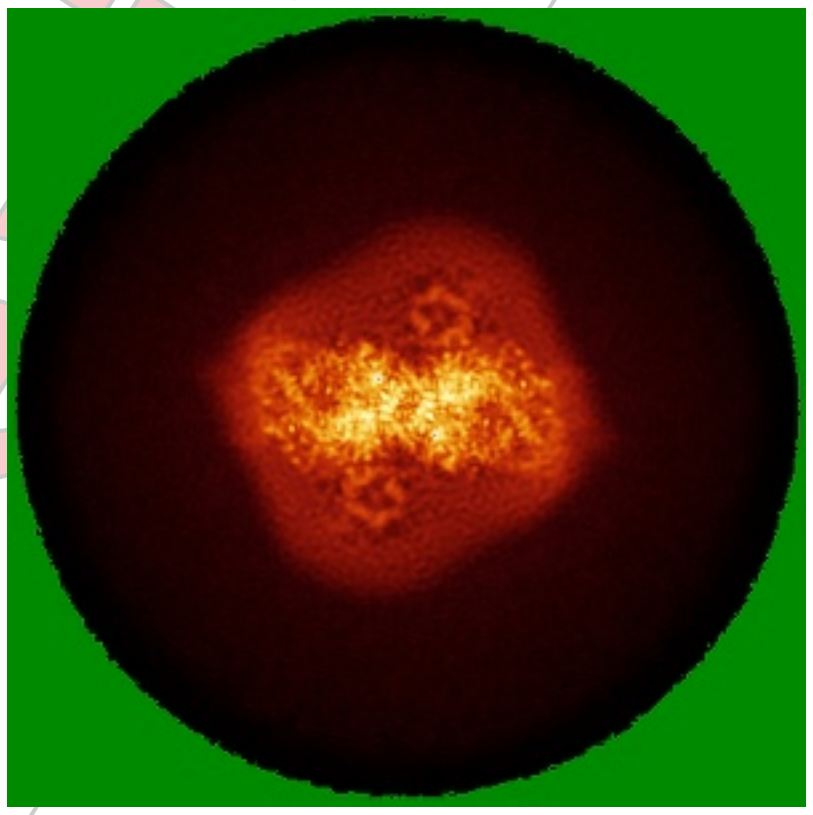
Z

6.4.2 Raw map

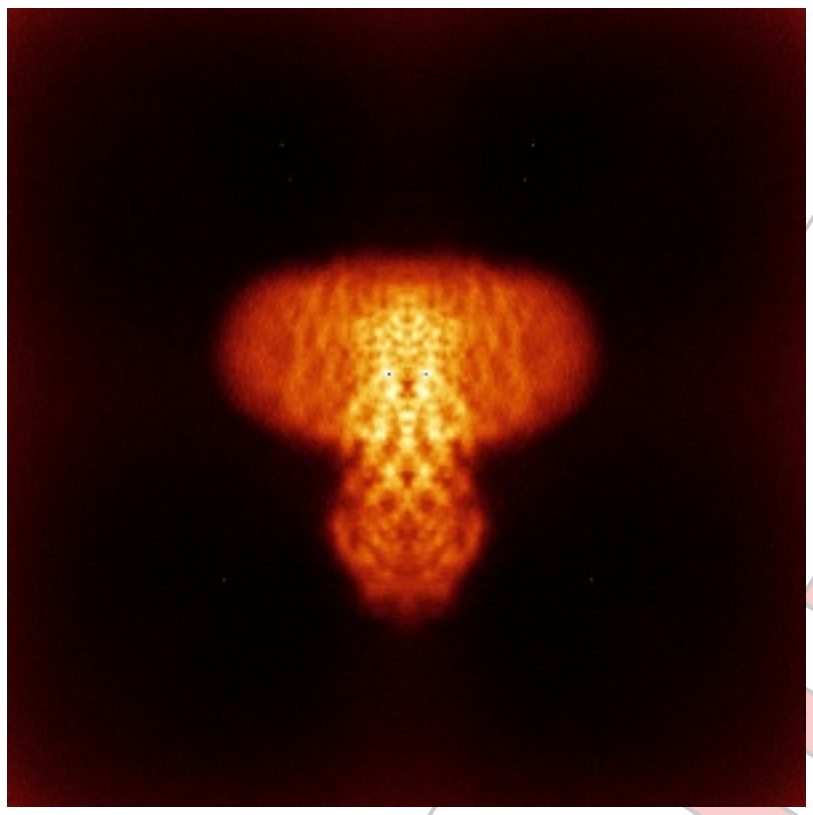
X

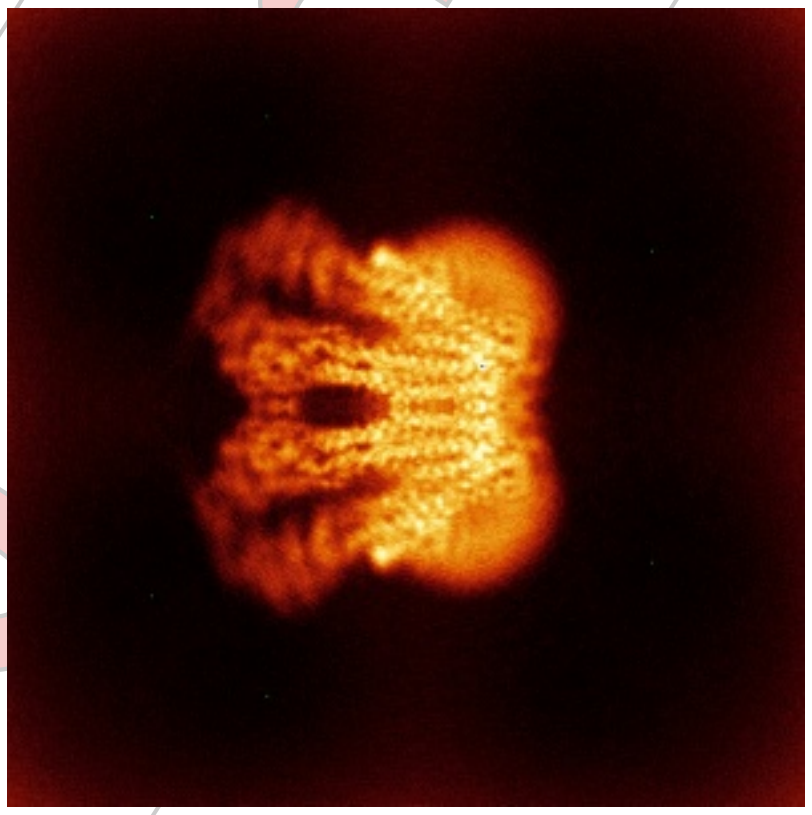
Y

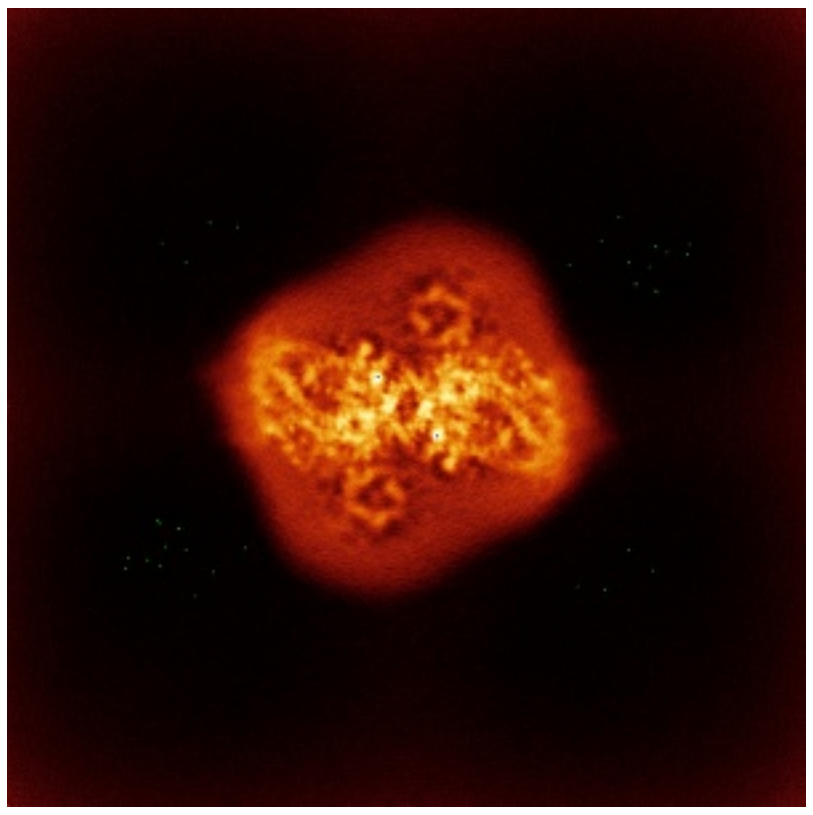
Z

The images above show the map standard deviation projections with false color in three orthogonal directions. Minimum values are shown in green, max in blue, and dark to light orange shades represent small to large values respectively.

For Manuscript Review

6.5 Orthogonal surface views (i)

6.5.1 Primary map

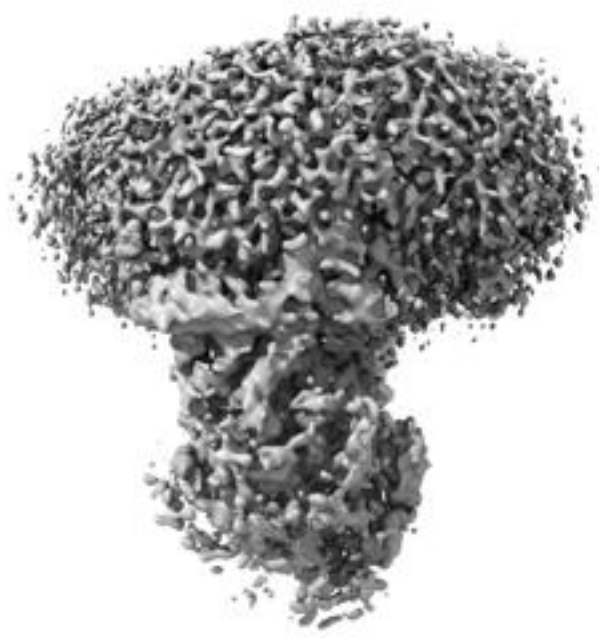

X

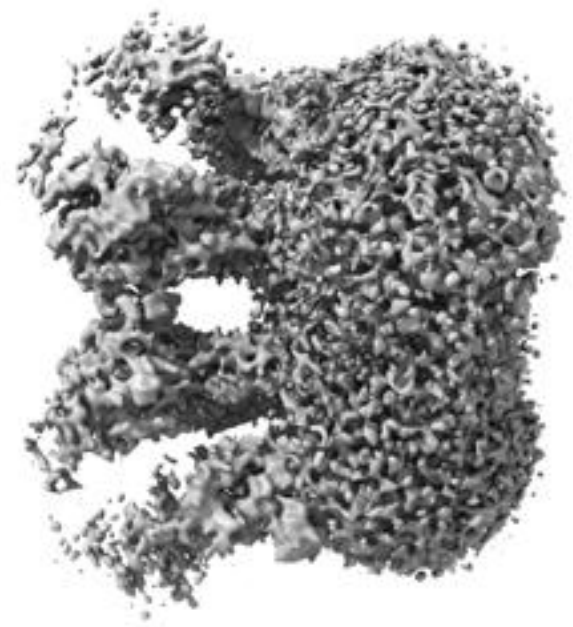

Y

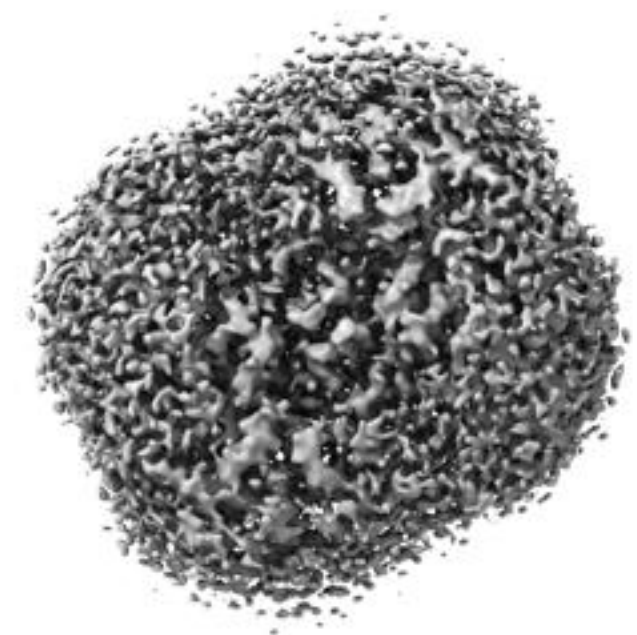

Z

The images above show the 3D surface view of the map at the recommended contour level 0.15. These images, in conjunction with the slice images, may facilitate assessment of whether an appropriate contour level has been provided.

6.5.2 Raw map

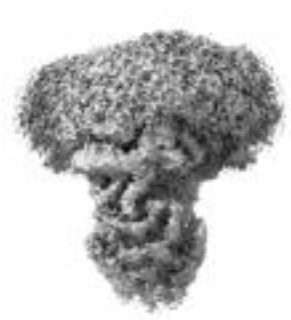

X

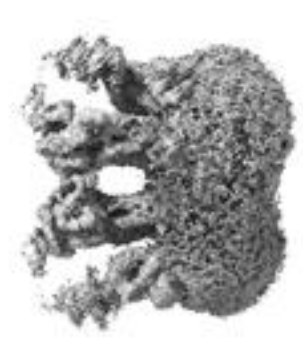

Y

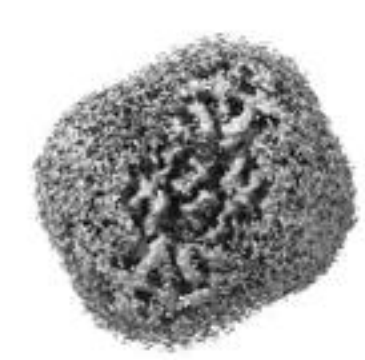

Z

These images show the 3D surface of the raw map. The raw map's contour level was selected so that its surface encloses the same volume as the primary map does at its recommended contour level.

6.6 Mask visualisation (i)

This section was not generated. No masks/segmentation were deposited.

For Manuscript Review

7 Map analysis (i)

This section contains the results of statistical analysis of the map.

7.1 Map-value distribution (i)

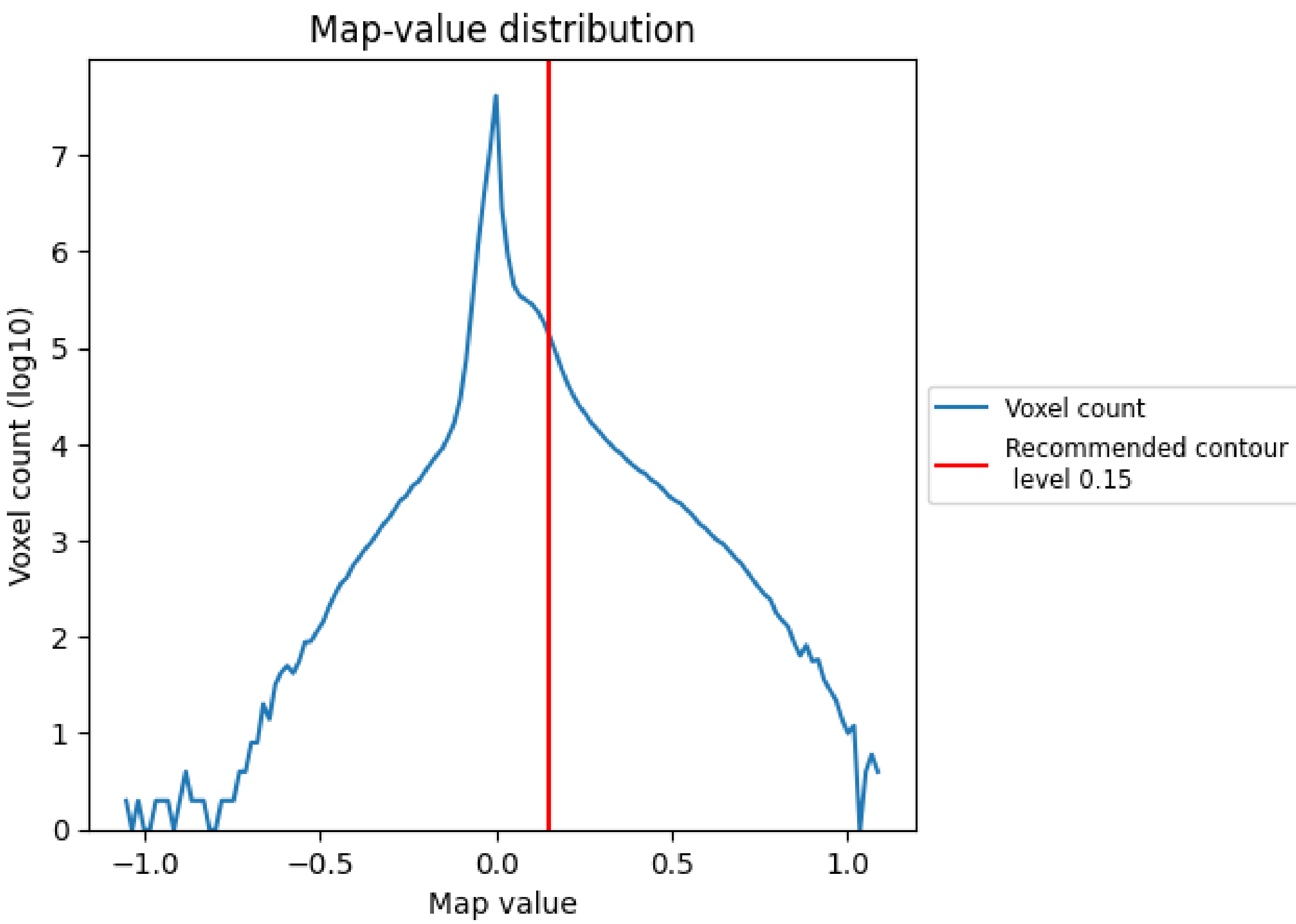

The map-value distribution is plotted in 128 intervals along the x-axis. The y-axis is logarithmic. A spike in this graph at zero usually indicates that the volume has been masked.

For Manuscript Review

7.2 Volume estimate (i)

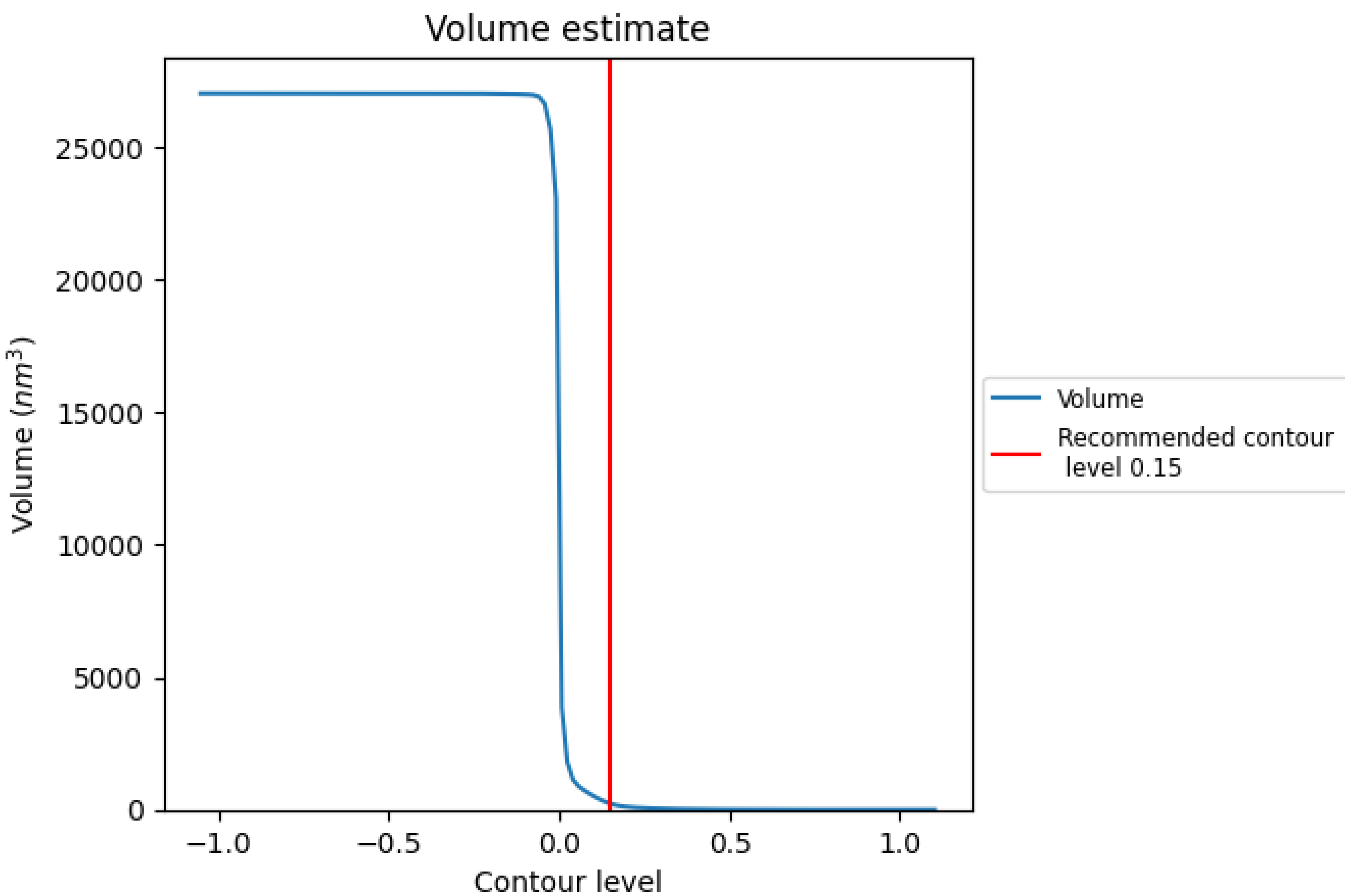

The volume at the recommended contour level is 235 nm^3; this corresponds to an approximate mass of 213 kDa.

The volume estimate graph shows how the enclosed volume varies with the contour level. The recommended contour level is shown as a vertical line and the intersection between the line and the curve gives the volume of the enclosed surface at the given level.

For Manuscript Review

7.3 Rotationally averaged power spectrum (i)

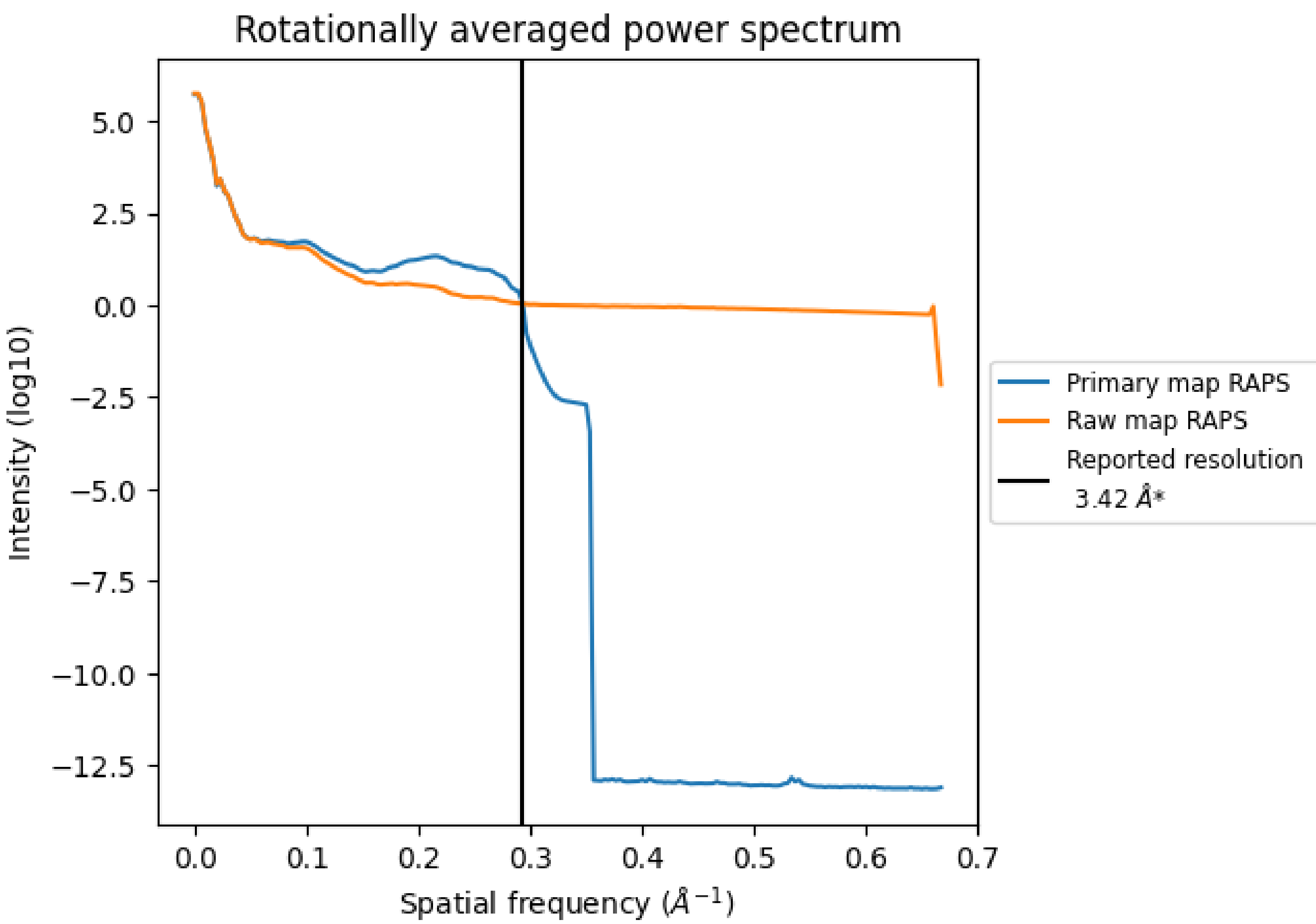

*Reported resolution corresponds to spatial frequency of 0.292 $Å^{-1}$

For Manuscript Review

8 Fourier-Shell correlation (i)

Fourier-Shell Correlation (FSC) is the most commonly used method to estimate the resolution of single-particle and subtomogram-averaged maps. The shape of the curve depends on the imposed symmetry, mask and whether or not the two 3D reconstructions used were processed from a common reference. The reported resolution is shown as a black line. A curve is displayed for the half-bit criterion in addition to lines showing the 0.143 gold standard cut-off and 0.5 cut-off.

8.1 FSC (i)

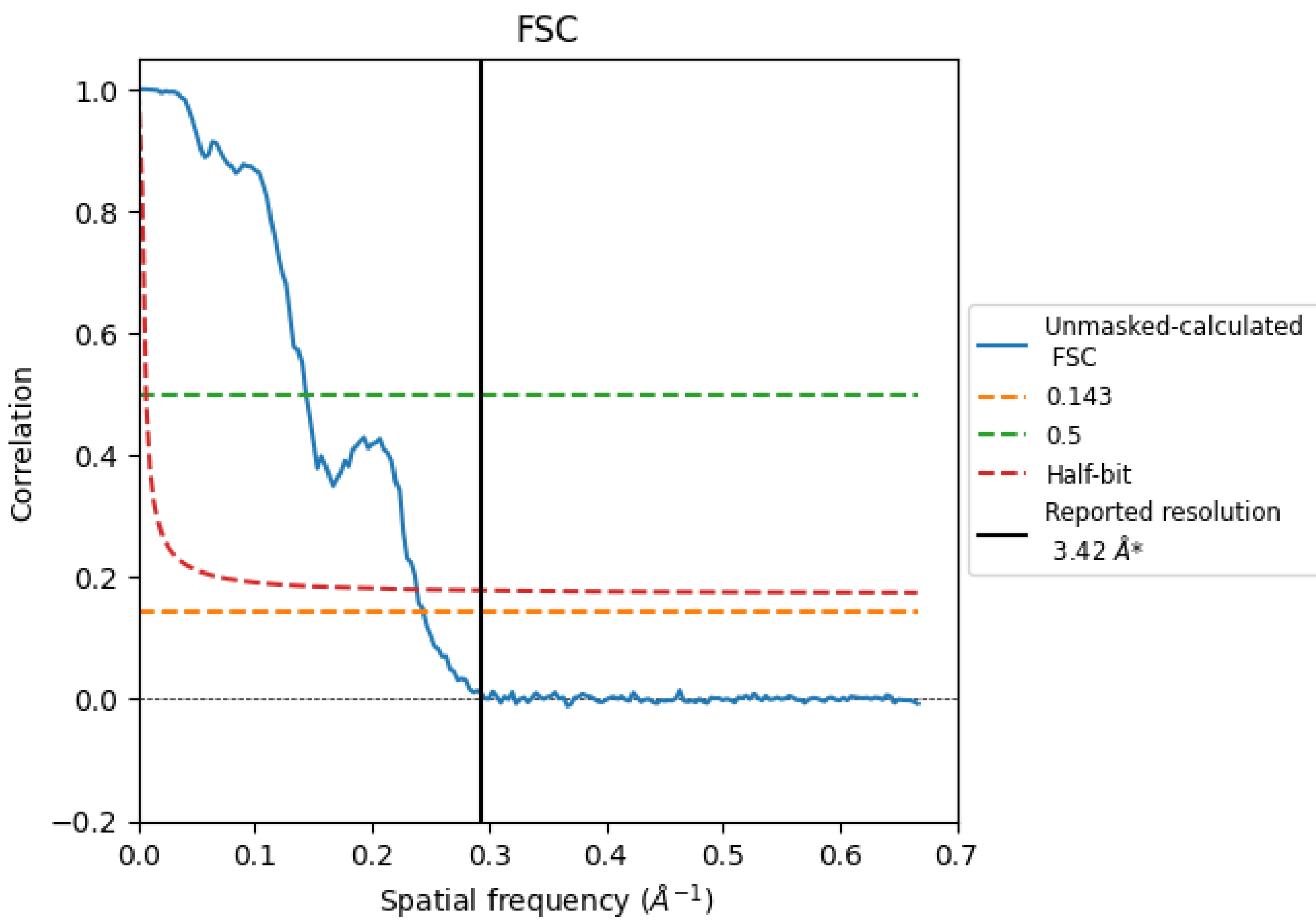

*Reported resolution corresponds to spatial frequency of 0.292 $Å^{-1}$

For Manuscript Review

8.2 Resolution estimates (i)

Resolution estimate (Å)	Estimation criterion (FSC cut-off)		
	0.143	0.5	Half-bit
Reported by author	3.42	-	-
Author-provided FSC curve	-	-	-
Unmasked-calculated*	4.10	6.98	4.19

*Resolution estimate based on FSC curve calculated by comparison of deposited half-maps. The value from deposited half-maps intersecting FSC 0.143 CUT-OFF 4.10 differs from the reported value 3.42 by more than 10 %

For Manuscript Review

9 Map-model fit (i)

This section contains information regarding the fit between EMDB map EMD-63865 and PDB model 9U57. Per-residue inclusion information can be found in section 3 on page 5.

9.1 Map-model overlay (i)

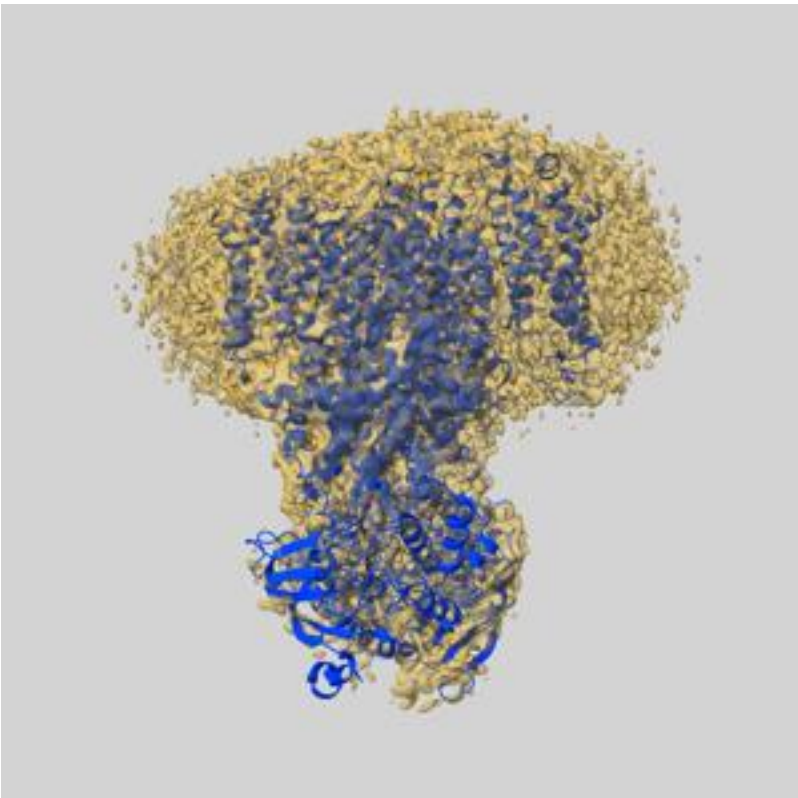

X

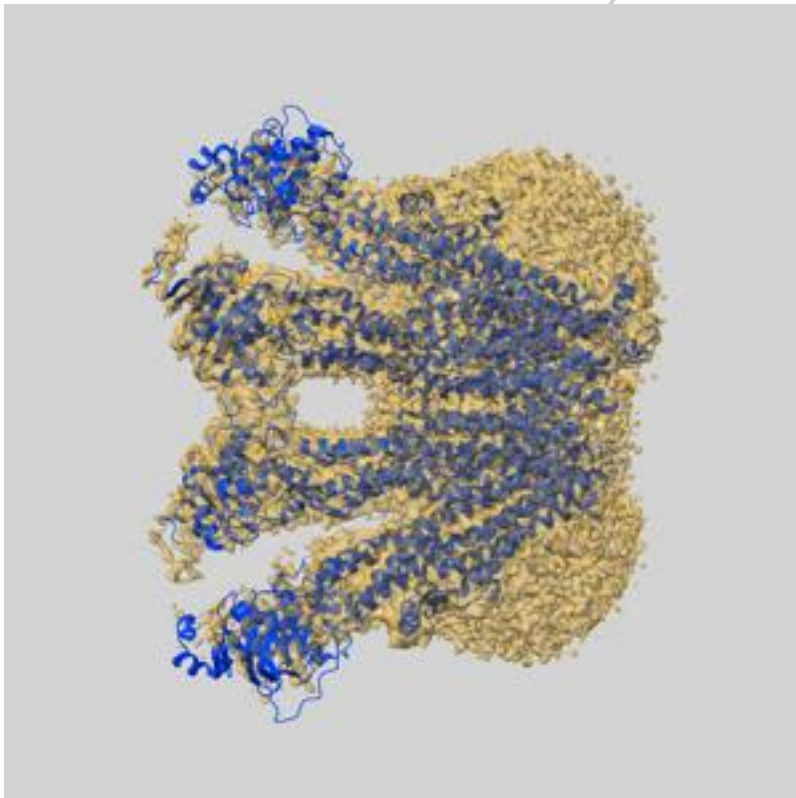

Y

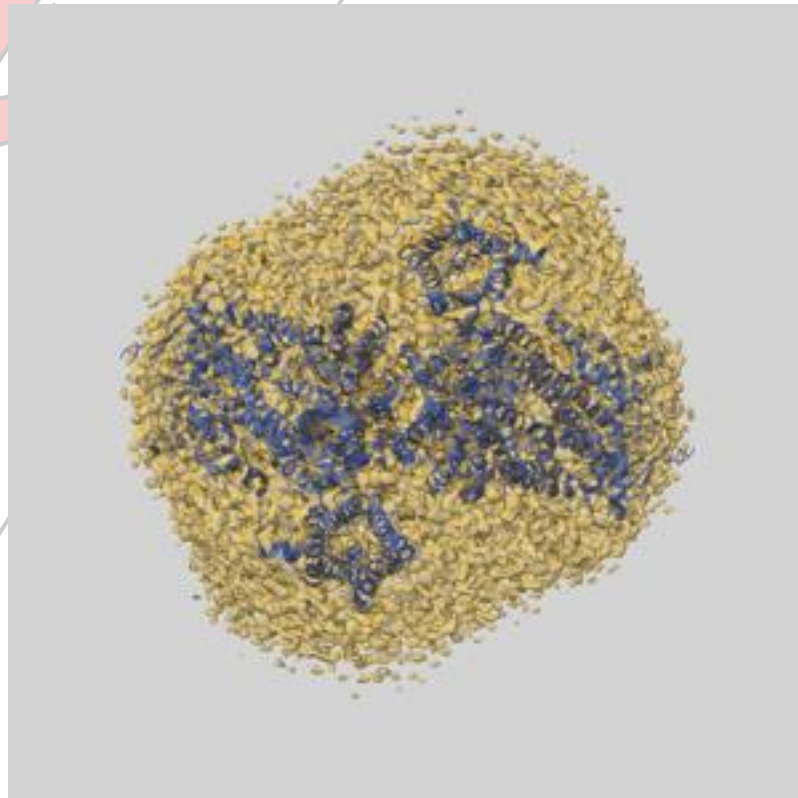

Z

The images above show the 3D surface view of the map at the recommended contour level 0.15 at 50% transparency in yellow overlaid with a ribbon representation of the model coloured in blue. These images allow for the visual assessment of the quality of fit between the atomic model and the map.

For Manuscript Review

9.2 Q-score mapped to coordinate model (i)

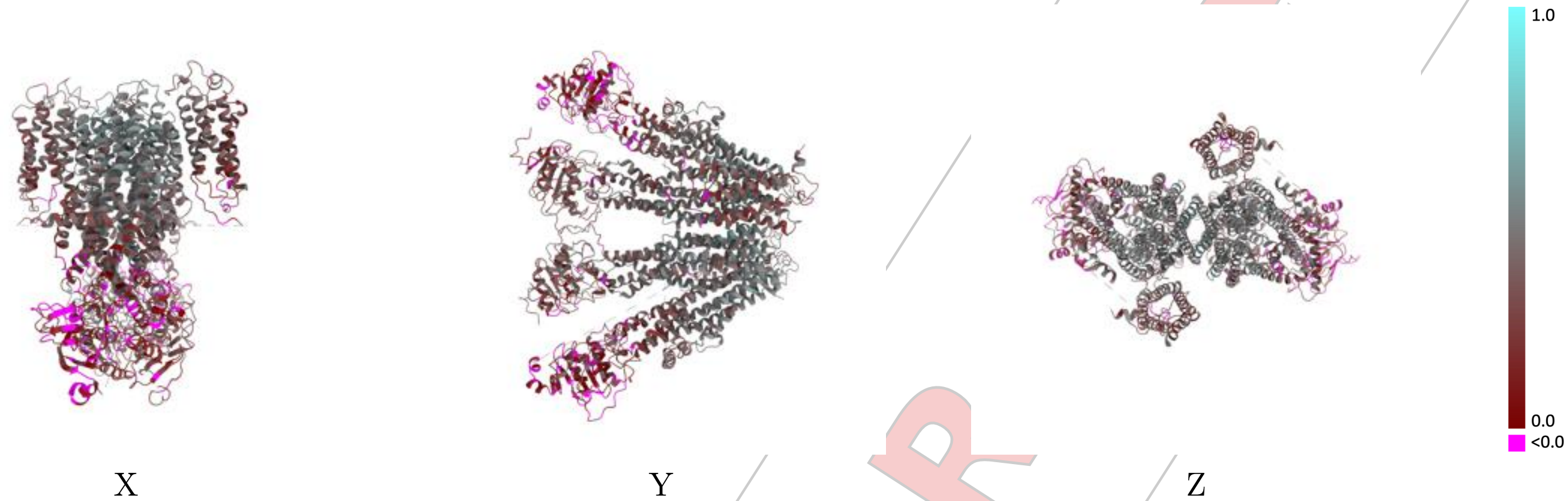

The images above show the model with each residue coloured according its Q-score. This shows their resolvability in the map with higher Q-score values reflecting better resolvability. Please note: Q-score is calculating the resolvability of atoms, and thus high values are only expected at resolutions at which atoms can be resolved. Low Q-score values may therefore be expected for many entries.

9.3 Atom inclusion mapped to coordinate model (i)

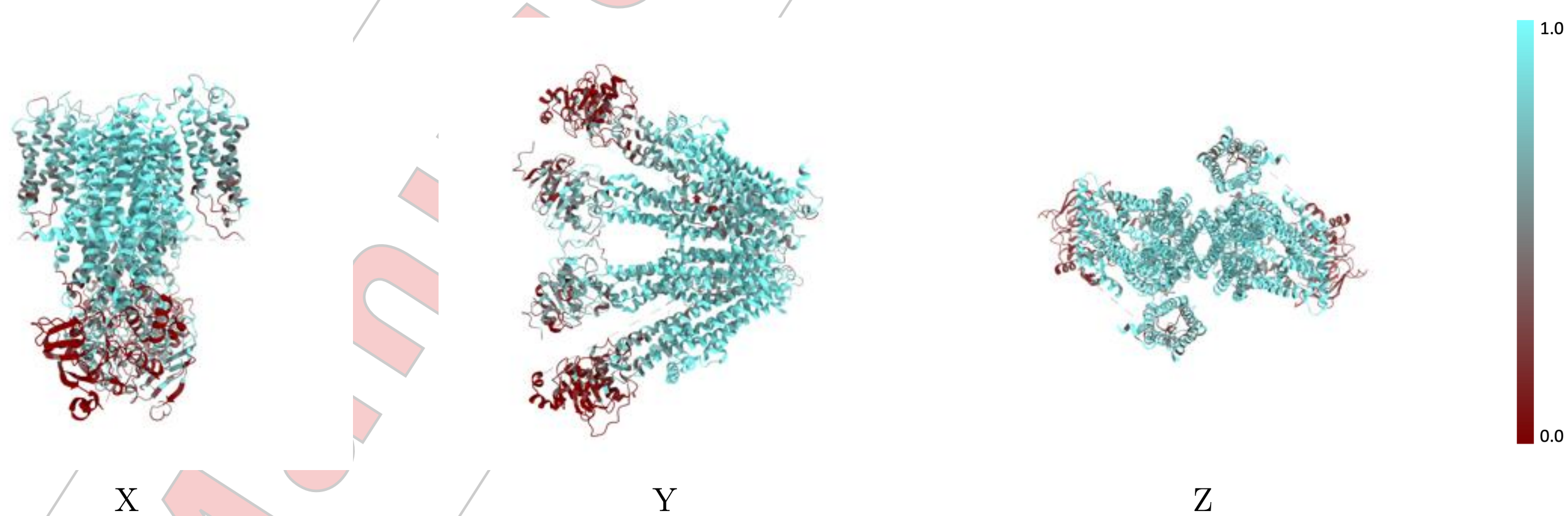

The images above show the model with each residue coloured according to its atom inclusion. This shows to what extent they are inside the map at the recommended contour level (0.15).

For Manuscript Review

9.4 Atom inclusion (i)

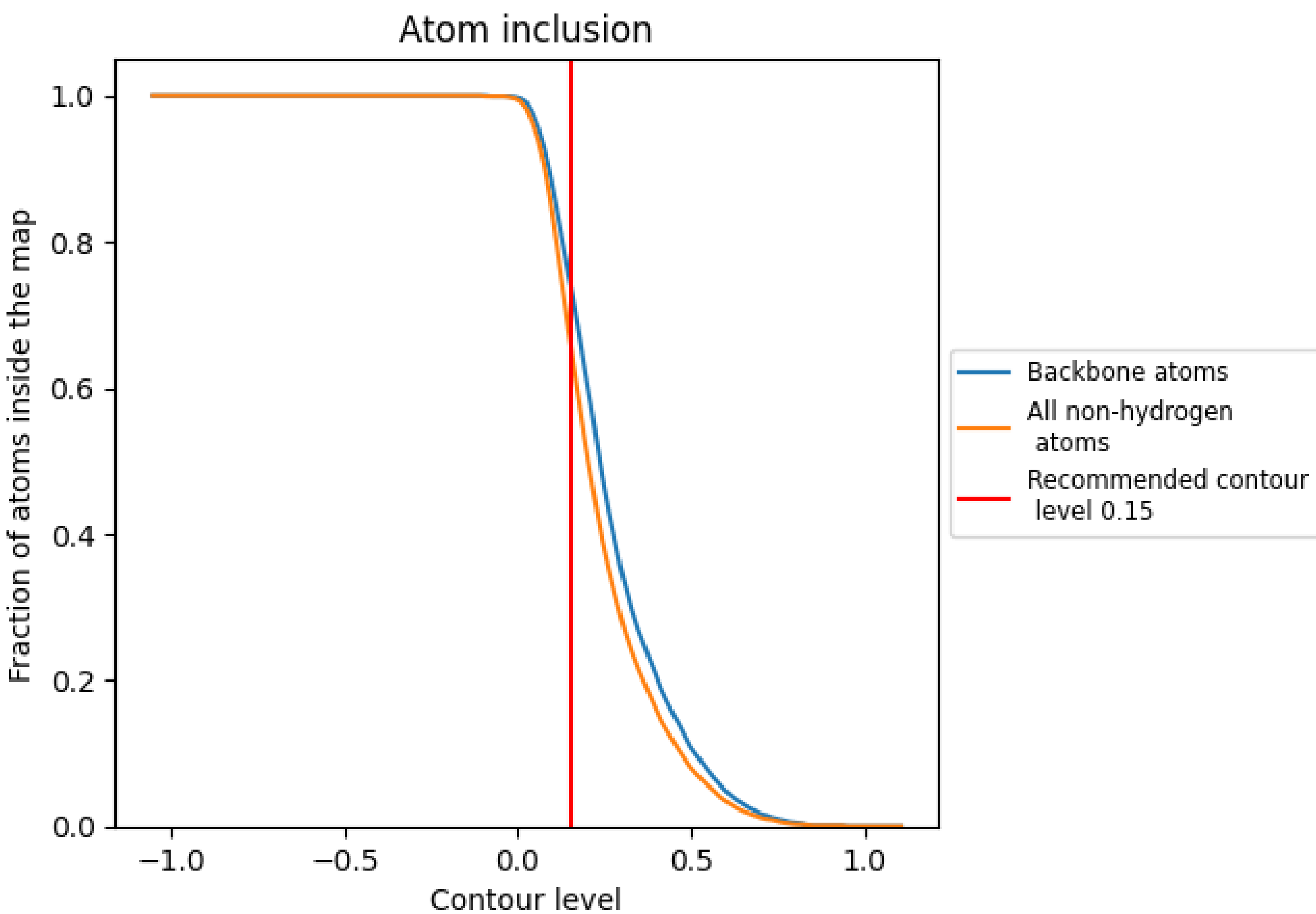

At the recommended contour level, 75% of all backbone atoms, 67% of all non-hydrogen atoms, are inside the map.

For Manuscript Review

9.5 Map-model fit summary (i)

The table lists the average atom inclusion at the recommended contour level (0.15) and Q-score for the entire model and for each chain.

Chain	Atom inclusion	Q-score
All	0.6670	0.3370
A	0.6680	0.3400
B	0.6660	0.3350

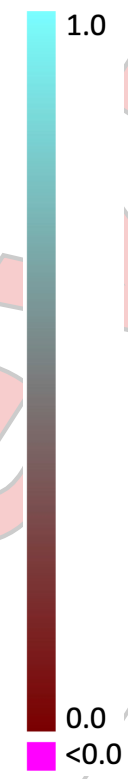

For Manuscript Review
